# Mo_4_FeGa_17.25– *x*_Ge_*x*_: Complementary Point Substitutions, Buffering Frameworks, and Merging of the 18‑*n* and Octet Bonding Schemes

**DOI:** 10.1021/acs.chemmater.5c03434

**Published:** 2026-05-03

**Authors:** Danica G. Gressel, Patrick K. Cross, Jonathan S. Van Buskirk, Daniel C. Fredrickson

**Affiliations:** Department of Chemistry, University of Wisconsin-Madison, 1101 University Avenue, Madison, Wisconsin 53706, United States

## Abstract

We present the discovery of Mo_4_FeGa_17.25–*x*_Ge_*x*_ (*x* ∼ 2.1(4), based on determination of Ge content), a complex gallide featuring a variety of point substitution phenomena. Its crystal structure is derived from the Ti_2_Ni type, in which a diamond network of face-sharing octahedra is interpenetrated by a second diamond network of vertex-sharing stella quadrangula. However, while in the ideal Ti_2_Ni type these two frameworks run uninterrupted through the crystal, Mo_4_FeGa_17.25–*x*_Ge_*x*_ shows three variations. First, the inner tetrahedron of every other stella quadrangula is replaced with a main group atom, creating tetrahedra reminiscent of the Zintl phase NaTl. Next, a selection of Ga_6_ octahedra are filled with Fe atoms, in a manner analogous to the stuffed AuCu_3_-type phases. Finally, the refined crystal structure shows that in each unit cell a single Ga2 atom in the octahedral network is substituted with a dumbbell of Ga/Ge atoms. Electronic structure calculations on ordered models of Mo_4_FeGa_17.25–*x*_Ge_*x*_ reveal a narrow band near the Fermi energy, which is explained in terms of the 18-*n* and octet bonding schemes using reversed approximation Molecular Orbital analysis. A DFT-chemical pressure analysis connects the tetrahedron/atom and atom/dumbbell substitutions to the relief of atomic packing tensions and highlights soft atomic motions within the octahedral framework and driving forces for atom/dumbbell substitution. The combination of soft vibrational modes, disorder, and a narrow band gap could make this phase of interest for potential thermoelectric properties.

## Graphical abstract

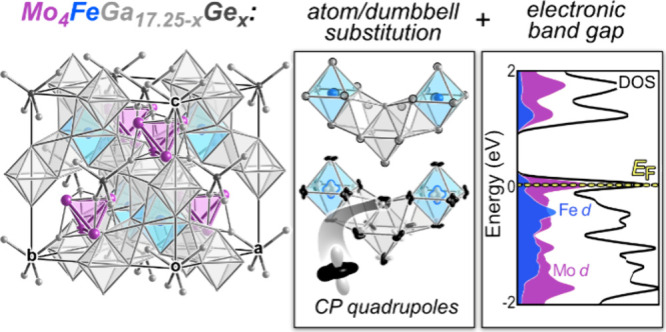

## 1 Introduction

Elemental substitution provides a crucial lever by which the properties of a material may be optimized. The structural chemistry of intermetallic phases offers additional substitutional variations that could be leveraged in modifying materials behavior. In some cases, pairs of atoms are replaceable by single atoms at their average position, as in the formation of the La_3_Al_11_ type^1^ or more complicated variations on the BaAl_4_ type.^2^ In some other structures, including Tsai-type quasicrystal approximants^3–5^ or Laves phases,^6,7^ entire tetrahedra of atoms can be replaced with a single, larger atom. Still other structures are able to incorporate guest atoms, reflecting the replacement of an interstitial space with an atom, as the stuffing of AuCu_3_- or CoSn-type frameworks in the Y_4_PdGa_12_ type^8–11^ or HfFe_6_Ge_6_-/ScFe_6_Ga_6_-type intergrowth structures.^12^ Any one of these phenomena opens a significant dimension for the optimization of the properties of a material. In this Article, we present the discovery of a compound which exhibits all three of these substitution types simultaneously, Mo_4_FeGa_17.25–*x*_Ge_*x*_.

This compound was encountered in the course of synthetic explorations of our recently proposed *interface nucleus approach* to the creation of new modular structures.^13–16^ In this model, different structures containing a shared motif may experience a driving force for intergrowth, particularly when that motif is subject to complementary packing strains in the two structures. After highlighting the σ-phase structure as a prototypical example, in which columns of the Cr_3_Si and Al_3_Zr_4_ types join at shared columns of icosahedra, we were intrigued to realize that the significantly more complex structure of Fe_18_Mo_26_Ge_9_ (Cr_0.16_Mo_0.38_Co_0.46_ type)^17^ can be interpreted in terms of the same structural themes, but with a more complex intergrowth topology.^18^ We were thus inspired to pursue its synthesis and a closer examination of its geometrical features.

Based on an analysis of the phase diagrams of binary systems combining Mo, Fe, and Ge with Ga, we concluded that it could be possible to crystallize Fe_18_Mo_26_Ge_9_ from a Ga flux. Instead, however, we obtained crystals of Mo_4_FeGa_17.25–*x*_Ge_*x*_. Its structure is based on the Ti_2_Ni structure type, in which a diamond framework of face-sharing octahedra is interpenetrated by a second diamond network built from vertex-sharing stella quadrangula.^19–21^ Mo_4_FeGa_17.25–*x*_Ge_*x*_, however, exhibits three distinct structural variations on this classic intermetallic prototype. First, a selection of the octahedra is occupied by Fe atoms. Second, half of the central tetrahedra in the stella quadrangula are replaced by single atoms, creating octet-like configurations. Finally, at other positions in the structure, some atoms within the framework of face-sharing octahedra are partially substituted by Ga/Ge dumbbells.

Through a bonding analysis of Mo_4_FeGa_17.25–*x*_Ge_*x*_, we will see that these features have consequences for its potential properties. Electronic structure calculations on an ordered model indicate that the Fermi energy lies very close to a band gap. A reversed approximation Molecular Orbital (raMO) analysis^22,23^ will connect this band gap to the satisfaction of the 18-*n* ^24–29^ and filled octet^30,31^ bonding schemes in different regions of the structure. In addition, the Fe atom incorporation and atom/dumbbell substitution will be traced to the typical DFT-Chemical Pressure (CP)^32–35^ features of octahedron-based buffering frameworks^36^ and their paths for soft atomic motion. Altogether these analyses hint at Mo_4_FeGa_17.25–*x*_Ge_*x*_ exhibiting semiconductor-like electrical conductivity with disorder and rattling units associated with low thermal conductivity, features that in combination could set the stage, within the phonon-glass/electron-crystal picture, for thermoelectric properties.^37–40^

## 2 Experimental Section

### 2.1 Synthesis of Mo_4_FeGa_17.25–*x*_Ge_*x*_

In the initial synthesis of the title compound, Mo powder (Strem, 99.95%), Fe powder (Strem, 99.9%), Ga droplets (Beantown Chemicals, 99.99%), and Ge powder (Alfa Aesar, −100 mesh, 99.99%) were combined in a molar ratio of 3:5:20:2 with the aim of synthesizing Fe_18_Mo_26_Ge_9_ from a Ga flux. To achieve this loading, elemental Mo, Fe, and Ge powders were first weighed out, ground together with a mortar and pestle, and pressed into pellets. The pellets were then melted with an arc melter under Ar, loaded into an alumina crucible (fashioned from an alumina tube and a cement base), and topped with a Ga droplet. The samples were next sealed in evacuated fused silica ampules, heated in a muffle furnace at 1,100 °C for 5 days, and finally quenched in ice water. The tubes were opened, and the samples were immersed in 3 M HCl for several minutes to remove excess Ga metal, revealing a dark, shiny crystalline powder with small gray crystals of the new compound Mo_4_FeGa_17.25–*x*_Ge_*x*_, along with crystals of FeGa_3_.

For the preparation of high yield samples of Mo_4_FeGa_17.25–*x*_Ge_*x*_, samples were loaded with a Mo:Fe:Ga:Ge molar ratio of 3.80:1.00:25.15:2.23 into a frit-disc crucible designed by Canfield and co-workers.^41^ The crucible assembly was then sealed in a fused silica tube under vacuum. The sample was heated at a rate of 2.8 °C/min to 700 °C and annealed at that temperature for 1 week. The sample was then inverted and centrifuged at 3,000 rpm to separate the products from the excess molten flux. Subjecting the products to an acid wash results in fewer impurities, but is not necessary for obtaining crystals of the title compound.

### 2.2 Powder X-ray Diffraction

Portions of the shiny gray samples were ground using a mortar and pestle, spread onto a zero-background Si plate, and pressed flat with a glass slide. Diffraction intensities were measured on a Bruker D8 Advance powder X-ray diffractometer equipped with a Cu Kα (λ = 1.5418 Å) X-ray source. The measurements were collected at room temperature with an exposure time of 0.9s per 0.021° increment over a 2θ range of 10–90°. The diffraction patterns were analyzed with the *Match!* Software.^42^

### 2.3 Wavelength Dispersive X-ray Spectroscopy

To determine the elemental composition of the title compound, wavelength-dispersive X-ray spectroscopy (WDS) measurements were performed on a sample for which it was the major phase. Small pieces of material were suspended in epoxy at the end of a short aluminum tube and cured for 12 h at 65 °C. A smooth surface exposing the sample was created by polishing with diamond lapping films (grits 9–0.5 μm). To ensure that the sample had a conductive surface, it was coated in a thin layer of carbon. WDS measurements were made with a Cameca SX100/SX-Five electron microprobe using as standards Mo metal, Fe metal, GaP, Ge metal for the Mo *L*α, Fe *K*α, Ga *K*α, Ge *K*α transitions, respectively, with a beam energy of 20 kV, a current of 20 nA, and a takeoff angle of 40°. The use of an LLIF crystal in the WDS measurements allows clear separation of the Ge *K*α and Ge *K*β transition peaks. Individual WDS data points are provided in the Supporting Information.

### 2.4 Single Crystal X-ray Diffraction

A dark gray crystal was fixed to the top of a thin glass fiber using epoxy, and diffraction intensities were measured using a Bruker D8 Venture Photon III four-circle diffractometer with a Diamond II IμS Mo Kα (λ = 0.71073 Å) radiation source. Data sets were collected at room temperature and at 100 K. The run lists were generated and frame data processed using the APEX6 software.^43^ The resulting diffraction patterns were indexed with a cubic cell with *a* = 11.53 Å (room temperature) and *a* = 11.51 Å (T = 100 K).

An evaluation of the Laue symmetry and the reciprocal lattice reconstructions indicated *Fm* 3̅ *m* (No. 225) as the highest possible space group assignment. However, when performing the charge-flipping algorithm^44,45^ with the SUPERFLIP program,^46^ the subgroup *F* 4̅3 *m* (No. 216) was recommended. Structure solution in this space group yielded 6 symmetry-distinct atomic positions. The structure was refined on F^2^ in JANA2006,^47^ resulting in a structure with a refined composition of Mo_4_Fe­(Ga/Ge)_17.25(3)_ (where Ge and Ga are combined in recognition that, as neighbors on the periodic table differing by only one in their electron count, X-ray diffraction with laboratory sources does not provide a meaningful distinction between them). Table 1 provides details regarding the refinement using the data collected at room temperature. Additional crystallographic details are provided in the Supporting Information.

**Table 1:** Crystal Data for Mo_4_FeGa_17.25–*x*_Ge_*x*_

refined composition	Mo_4_Fe(Ga/Ge)17.25(3)
WDS composition	Mo4.0(4)Fe1.0(1)Ga15.4(9)Ge2.1(4)
crystal dimension (mm^3^)	0.020 × 0.035 × 0.020
crystal color	dark gray, metallic sheen
data collection temperature	ambient
radiation source, λ (Å)	Mo Kα, 0.71073
absorption correction	multiscan
space group	*F* 4̅ *3m* (No. 216)
*a* (Å)	11.5272(15)
Cell volume (Å^3^)	1,531.7(3)
*Z*	4
absorption coefficient (mm^–1^)	33.979
θ_min_, θ_max_	3.06, 35.97
refinement method	F^2^
R_int_ (obs., all)	0.0343, 0.0344
number of reflections	10,323
number of parameters	25
unique reflections (I > 3σ, all)	409, 412
R(I > 3σ), R_w_(I > 3σ)	0.0159, 0.0160
R(all), R_w_(all)	0.0389, 0.0389
S(I > 3σ), S(all)	1.51, 1.52
Δρ_max_, Δρ_min_ (e^–^/Å^3^)	0.96, −0.64

### 2.5 Electronic Structure Calculations

For the analysis of the bonding and electronic structure features of Mo_4_FeGa_17.25–*x*_Ge_*x*_, density functional theory (DFT) calculations were performed on ordered models with the Vienna ab initio Simulation Package (VASP),^48–51^ using the PW91 ^52,53^ functional and projector augmented wave potentials.^54,55^ Two models were explored, one in which the occupancies were simply rounded to 1 or 0 (i.e., no dumbbell substitution), and one in which a single Ga2 atom is substituted with a Ga4 dumbbell. All Ga/Ge positions were modeled with Ga atoms except that Ge is placed at the Ge1 site (where the small multiplicity allows full occupancy by Ge without leading to a significant excess of Ge in the overall structure; the problem of identifying an optimal placement of Ge on a subset of symmetry-equivalent sites, with a necessary lowering of symmetry, is thus avoided). The structures were geometrically optimized, beginning with a relaxation of the atomic positions within a fixed unit cell and then proceeding to the release of all structural parameters. All k-point mesh dimensions and energy cutoff values were selected such that the total energy was converged to within 5 meV/atom. Further computational details are listed in the Supporting Information.

DFT-reversed approximated Molecular Orbital (raMO) analysis^22,23^ was carried out with the DFTraMO.jl program^56,57^ on the PW91 electronic structure of the dumbbell-free model. As input for the method, wavefunctions distributed over a 2 × 2 × 2 Γ-centered k-point mesh were used. *r*_sphere_ values were set to 2.53, 2.33, and 2.66 Å for the *P*_sphere_ analysis^58^ of the Ge-, Fe-, and Mo-atom-centered raMO functions, respectively. An *r*_sphere_ value of 3.50 Å was employed for the Mo–Mo isolobal bonds.

For DFT-Chemical Pressure (CP) analysis,^32^ electronic structure calculations on the dumbbell-free model were performed with the ABINIT program^59,60^ using the standard platform: the LDA functional^61^ and the Hartwigsen-Goedecker-Hutter (HGH) norm-conserving pseudopotentials.^62^ A 3 × 3 × 3 Γ-centered k-point mesh was employed with the energy cutoff being set to 70 Ha. Following the optimization of the crystal structure, single point calculations were made on the equilibrium geometry as well as slightly expanded and contracted versions (linear scale 1.00 ± 0.005) for the generation of the wavefunctions, Kohn–Sham potential components, and kinetic energy and electron densities used for the creation of CP maps.

The CP maps were constructed with the CPpackage software,^63^ using core unwarping,^64^ the mapping of nonlocal energy terms,^34^ and the self-consistent partitioning of the Ewald + α energies.^35^ For all needed free ion calculations, the Atomic Pseudopotentials Engine (APE)^65^ was called. Interpretation of the CP maps in terms of interatomic interactions was accomplished with the iterative binary Hirshfeld-contact volume scheme.^35^ Pressures within the contact volumes were averaged and projected onto atom-centered spherical harmonics for visualization.

The ABINIT software was also used for the calculation of the phonon band structure of the dumbbell free model. A 3 × 3 × 3 Γ-centered q-point grid was sampled using the density functional perturbation theory (DFPT) approach as implemented in the ABINIT software suite.^66^ After constructing the dynamical matrices via DFPT,^66^ the band structure was assembled by interpolating along the k-space path: Γ–X–W–K–Γ–L–U–W–L–K with 40 points between the high symmetry points. This calculation was performed using the PBE functional^67,68^ and PAW potentials provided with ABINIT. The choice of functional is observed to have very little impact on the general features of the compound. There is a small change in lattice parameters when compared to the LDA-optimized structure (<5%), while the electronic density of states (DOS) distribution is essentially unchanged compared to the LDA electronic structure used for the CP analysis.

For the analysis of the energetics of dumbbell incorporation, total energy calculations were also performed on a dumbbell-free model and dumbbell-containing model (both using the conventional cell), as well as elemental Ga, using the VASP software package. A 3 × 3 × 3 Γ-centered k-point mesh and energy cutoff of 225 eV were used for both models of the title compound. A 7 × 7 × 7 Γ-centered k-point mesh and 260 eV energy cutoff were used for the calculation on elemental Ga. All calculations used the PW91 functional and PAW potentials provided with the package.

Electronic DOS distributions were generated for the PAW–PW91, PAW–PBE, and HGH-LDA electronic structures obtained respectively with VASP, ABINIT, and ABINIT. These calculations employed 15 × 15 × 15 Γ-centered k-point meshes and the corresponding energy cutoff values previously detailed. Projected DOS distributions were made for the PAW calculations using the PAW sphere radii for the projections. For the PW91 case, the radii are 1.46, 1.22, 1.40, and 1.30 Å for the Mo, Ge, Ga, and Fe potentials, respectively. For the PBE case, the corresponding radii are 1.16, 1.22, 1.11, and 1.12 Å.

## 3 Results and Discussion

### 3.1 Synthetic Results

In our pursuit of structures related to the interface nucleus approach, we identified the Mo–Fe–Ge system as fertile grounds for discovery. Its phase diagram contains binary compounds with structure types we noted earlier to have a potential for CP-driven intergrowth: MoFe_2_ ^69^ in the MgZn_2_ type and Mo_3_Ge in the Cr_3_Si type. One of its few known ternary phases, Fe_18_Mo_26_Ge_9_ (Cr_0.16_Mo_0.38_Co_0.46_ type),^18^ has a curious crystal structure whose local features recall the CP-based matching rules between the Cr_3_Si and Al_3_Zr_4_/Laves phase types derived from the σ-phase structure,^70^ but the published model exhibits significant disorder.

Intrigued about the connections between this compound and the interface nucleus approach, we set out to reinvestigate its structure. After a few attempts to synthesize Fe_18_Mo_26_Ge_9_ from its component elements, we concluded that a flux synthesis may provide higher quality crystals. From an examination of the Fe–Ga, Mo–Ga, and Mo–Ge phase diagrams, we determined that above 1100 °C no binary gallide phases would form, suggesting that Ga would be a good flux medium. However, our attempts at flux syntheses resulted in a minor unknown phase, several Ga-containing side products, such as FeGa_3_ (CoGa_3_ type),^71^ Mo­(Ga/Ge)_2_ (CrSi_2_ type),^72,73^ and Mo_8_Ga_41_ (V_8_Ga_41_ type), and excess Ga metal.^74,75^

We pursued the unknown compound through an adaptation of the synthetic procedure, in which we heated at lower temperatures (700 °C) and used Canfield crucibles to filter off the excess Ga-rich liquid. The reaction product obtained contained higher amounts of the unknown phase, as revealed by powder X-ray diffraction (Figure 1a).

**Figure 1:**
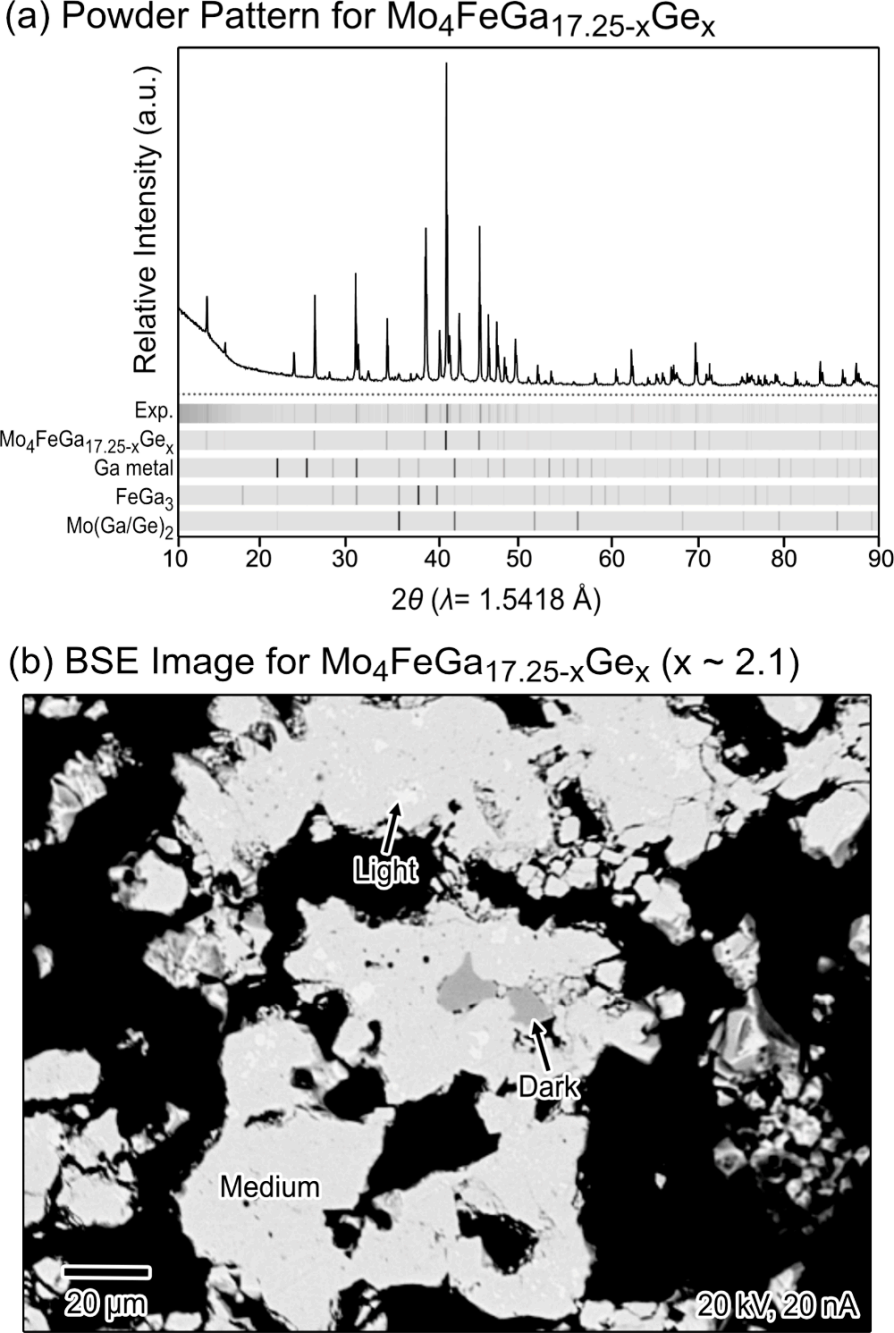
Phase analysis of a Mo_4_FeGa_17.25–*x*_Ge_*x*_ sample. (a) Powder X-ray diffraction pattern, with the calculated intensities for the refined structure of Mo_4_FeGa_17.25–*x*_Ge_*x*_ and other compounds plotted for comparison. (b) Scanning electron microscope (SEM)-backscattered electron (BSE) image of a polished portion of the sample.

Metallographic analysis confirmed this overall picture. SEM-BSE imaging of a polished surface (Figure 1b) revealed a majority phase appearing as medium gray, embedded with minor phases in lighter and darker gray. WDS measurements on the major phase yielded the composition Mo_4.0(4)_Fe_1.0(1)_Ga_15.4(9)_Ge_2.1(4)_, which is within the standard deviation of the Mo:Fe:(Ga/Ge) ratio obtained from single crystal refinement of the title compound. Based on the WDS analysis, the minor phases were assigned as FeGa_3_ (dark phase) and Mo­(Ga/Ge)_2_ (light phase).

Following the structure solution of the title compound, its similarities to the previously described gallide Cr_4_PtGa_17_ ^76^ prompted us to pursue a Ge-free analogue. Despite several attempts, however, we were unable to obtain such a phase, even in trace amounts, without including Ge in the reaction.

### 3.2 Structure Solution and Refinement

Several small, dark gray crystals selected from the samples exhibited X-ray diffraction peaks corresponding to a cubic unit cell with *a* = 11.53 Å. Based on the Laue symmetry, systematic absences, and symmetry of the electron density maps from the charge-flipping algorithm, we assigned the space group *F* 4̅3 *m* to the structure. The structure solution yielded six symmetry-distinct atomic positions, one each for Mo (Mo1) and Fe (Fe1), and four that can be assignable to either Ga or Ge. We labeled one of these as Ge1 due to the similarity of its tetrahedral coordination environment to that in elemental Ge, and the others as Ga2, Ga3, and Ga4.^77^ The WDS measurements suggest that the average Ga:Ge ratio across these sites is about 88:12, but the difference in the form factors for these two elements is not sufficient to evaluate potential site preferences. These assignments are thus only tentative.

In the refinement of this model, curious residual density peaks of approximately 3 electrons/Å^3^ appear on opposite sides of each Ga2 atom, on the way to the nearest Fe1 sites. These positions are too close to the Ga2 site for simultaneous occupancy. The refinement is drastically improved by modeling the Ga2 atom as partially replaced by a dumbbell of Ga atoms (Ga4) with each of the dumbbell atoms occurring at one of these peaks. The occupancies of the Ga2 and Ga4 sites converged to 0.9587(18) and 0.0413(18), respectively, under the restraint that they add up to 1. With the inclusion of this disorder, reasonable refinement statistics were obtained (Table 1), with no significant features in the Fourier difference map. The partially occupied Ga4 site exhibits unusually short contacts with neighboring Ga1 positions, but this is somewhat accounted for by the elongated atomic displacement parameters on the latter.

Interested in the relative role of vibrational and static displacements in this disorder, we collected a second data set at T = 100 K. The Ga2 and Ga4 site occupancies are largely unchanged, consistent with a static single atom/dumbbell substitution pattern. However, the Ga1 position now exhibits a clear splitting into a majority site near the original (Ga1a) and one further from the Ga4 position (Ga1b). The occupancy of Ga1b is essentially equal to that of Ga4, suggesting that it represents alternative Ga1 position that is used when an atom is present at the nearby Ga4 site. We refer to the resulting structure with the formula Mo_4_FeGa_17.25–*x*_Ge_*x*_, where 17.25 gives the total Ga/Ge content of the refined structure. *x* is shown as a variable to denote the uncertainty in the Ge content, rather than to imply an established phase width; its value is approximately 2.1(4) according to the WDS data.

For both the room temperature and T = 100 K refinements, the interatomic distances largely lie within the expected ranges, as judged from histograms generated by the Inorganic Crystal Structure Database.^78,79^ The chief exception is the Fe–Ga1 distance. These contacts define Fe@(Ga/Ge)_6_ octahedra with an Fe-(Ga/Ge) distance of 2.336 Å at room temperature. This distance is somewhat shorter than that observed for a similar unit in the compound Y_4_FeGa_12_, 2.439 Å,^80^ and may point to partial occupation of the site by Ge. Overall, the distances in low temperature model are contracted relative to their counterparts at room temperature, with decreases of several thousandths of an Å or increases that are within the standard uncertainties.

### 3.3 The Crystal Structure of Mo_4_FeGa_17.25–*x*_Ge_*x*_

In describing the crystal structure of Mo_4_FeGa_17.25–*x*_Ge_*x*_, it is useful to begin with a simpler parent structure, the Ti_2_Ni type (Figure 2a). Ti_2_Ni exhibits a double-diamond architecture, in which a framework of face-sharing Ti octahedra tracing out a diamond network is interpenetrated by a second diamond network, this time of corner-sharing stella quadrangula. Here each stella quadrangula consists of a Ni_4_ tetrahedron whose triangular faces are capped by Ti atoms, which are shared between neighboring units. The Ti_2_Ni type on its own is known for structural variations. For example, its octahedral holes can host interstitial atoms, such as of C, N, or O.^81–85^

**Figure 2:**
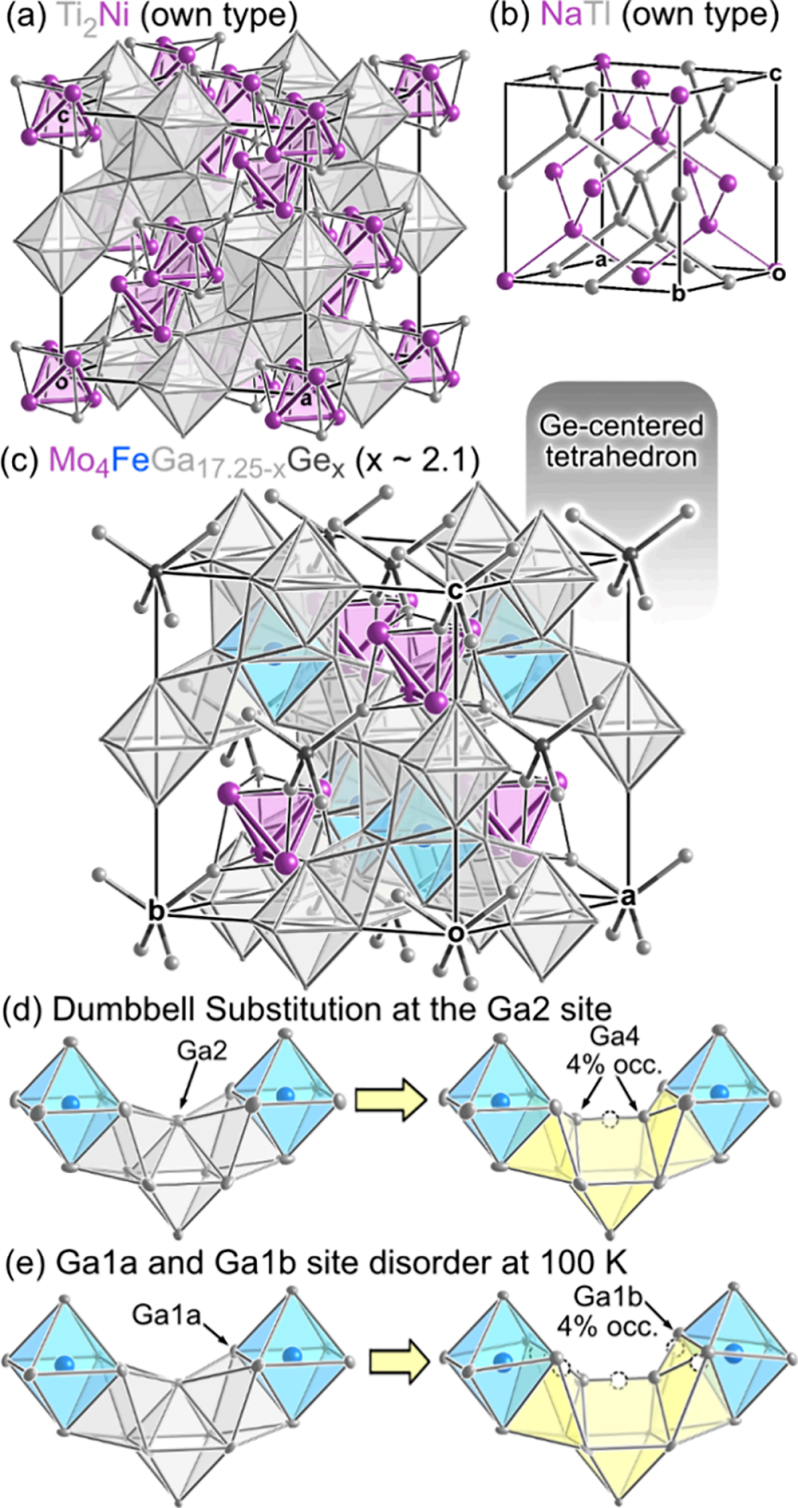
The crystal structure of Mo_4_FeGa_17.25–*x*_Ge_*x*_ (*x* ∼ 2.1). (a) The Ti_2_Ni type, the parent structure. (b) The structure of the Zintl compound NaTl, a second structure related to Mo_4_FeGa_17.25–*x*_Ge_*x*_. (c) The refined structure of Mo_4_FeGa_17.25–*x*_Ge_*x,*_ with the Ge/Ga atoms substituting for tetrahedra highlighted. (d) A piece of the octahedral network for the room temperature model with 75% probability ellipsoids, showing scenarios with and without the substitution of a Ga2 with a Ga/Ge dumbbell. (e) Corresponding images for the T = 100 K refinement.

In Mo_4_FeGa_17.25–*x*_Ge_*x*_, these variations are taken to an extreme. The roles of Ti and Ni in the basic structure are taken by Mo and Ge/Ga, respectively, but this is just the beginning. Whereas in the parent Ti_2_Ni structure all the octahedra are unfilled, here every other octahedron with tetrahedral site symmetry is stuffed with an Fe atom (Figure 2c). As a consequence, the contacts along the edges of these octahedra are significantly longer (3.303 Å) than those in their unstuffed counterparts (2.976 Å). This distinction between filled and empty 4-fold-connected octahedra in the framework lowers the symmetry from the original *Fd* 3̅ *m* of the Ti_2_Ni type to *F* 4̅3 *m*.

Another key variation is within the network of stella quadrangula. In Mo_4_FeGa_17.25–*x*_Ge_*x*_, every other tetrahedron of Ni-type atoms in the Ti_2_Ni type is replaced with a single Ga/Ge atom, labeled as Ge1 site (Figure 2c). This Ga/Ge atom occupies a tetrahedron of Ga3 atoms, taking on a tetrahedral coordination similar to that seen in elemental Ge or the anions in NaTl-type Zintl phases (Figure 2b),^86^ in contrast to the denser clustering exhibited by the remaining main group sites. This variation in every other stella quadrangula also forces a lowering of the space group from *Fd* 3̅ *m* to *F* 4̅3 *m.* With these variations alone, Mo_4_FeGa_17.25–*x*_Ge_*x*_ would be essentially isostructural with the compound Cr_4_PtGa_17_ reported by Gui and co-workers.^76^ The chief difference is that Cr_4_PtGa_17_ is presented in a rhombohedral subgroup of *F* 4̅3 *m*, whereas in the case of Mo_4_FeGa_17.25–*x*_Ge_*x*_ we did not detect any indications for a lower space group symmetry.

Mo_4_FeGa_17.25–*x*_Ge_*x*_, however, has one additional structural complication (Figures 2d-e). The Ga2 site, at the corners of the vacant octahedra with tetrahedral site symmetry, are partially replaced by a dumbbell (Ga4 site) that places Ga/Ge atoms closer to the nearby Fe atoms within the octahedral framework. At 4% substitution for 24 Ga2 sites/cell, there is approximately 1 such dumbbell per unit cell, corresponding to one of the four (Ga2)_6_ octahedra per cell in fact being a monocapped trigonal prism. The placement of the Ga4 atoms into the structure leads to tight contacts with the Ga1 site, making its elongated thermal ellipsoid in the room temperature refinement (Figure 2d) and formation of split position at T = 100 K (Figure 2e) understandable.

### 3.4 Band Gap and Electron Counting in Mo_4_FeGa_17.25–*x*_Ge_*x*_

The structure of Mo_4_FeGa_17.25–*x*_Ge_*x*_ poses an interesting challenge from the point of view of bonding. Its composition gives it a transition metal (T = Mo and Fe) to main group (E = Ga and Ge) atom ratio of approximately 1:3.4, placing the compound within the range of validity for the 18-*n* rule for T–E intermetallics.^25^ At the same time, the E@E_4_ tetrahedra centered by the Ge1 site are reminiscent of elemental Ge or the Zintl phase NaTl,^86^ where the simple octet rule is more applicable. How do these two bonding pictures come together in this compound?

The electronic density of states (DOS) distribution for a dumbbell-free version of the structure gives important context for this discussion (Figure 3). The DOS distribution shows a familiar organization for T–E intermetallics,^87^ beginning with a parabolic curve dominated by Ga/Ge valence s orbital character at lower energies. At somewhat higher energies, a block of Mo/Fe-rich bands appears with contributions from the Ga/Ge p orbitals (see the Supporting Information). Also, as in many other T–E phases, the Fermi Energy (*E*_F_) lies near a DOS minimum corresponding to a closed shell configuration. In this case, it is a full band gap of approximately 0.6 eV, as found previously for Cr_4_PtGa_17_,^76^ corresponding to a valence electron count of 86 electrons per formula unit. With the ordered model having a composition of Mo_4_FeGa_16_Ge (83 electrons per formula unit), the *E*_F_ lies somewhat short of the band gap. This difference could be decreased, of course, by further replacement of Ga with Ge; going to Mo_4_FeGa_14_Ge_3_ would achieve the 86 electron count.

**Figure 3:**
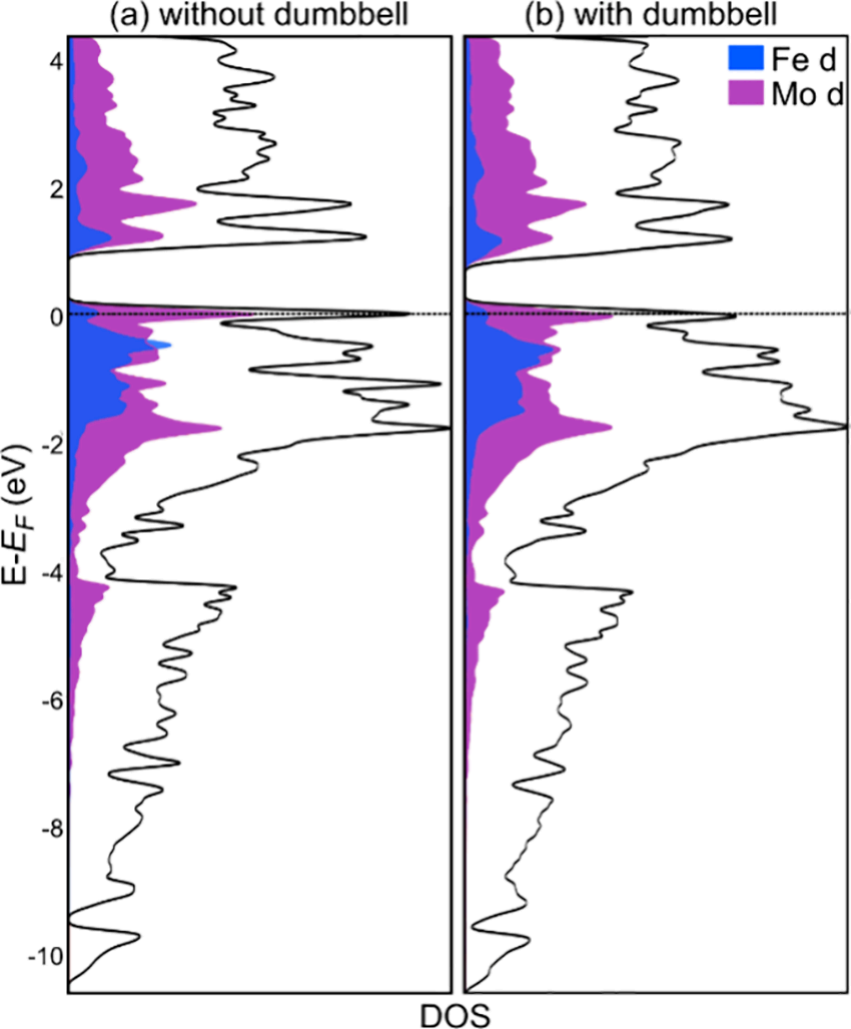
Electronic density of states (DOS) distributions calculated for (a) a dumbbell-free model of Mo_4_FeGa_17.25–*x*_Ge_*x*_ with composition Mo_4_FeGa_16_Ge_1_, and (b) a model with one Ga2 site per conventional unit cell replaced with a Ga dumbbell and composition Mo_4_FeGa_16.25_Ge_1_. The projected DOS contributions from the Mo and Fe d orbitals are shaded in purple and blue, respectively. Gaussian broadening (using a normalized Gaussian with a standard deviation of 0.05 eV) has been applied to bring out the general features of the distributions.

The presence of the band gap in the electronic structure signals that the electron count of 86/formula unit coincides with a closed shell configuration. Drawing on the 18-*n* and octet bonding schemes, a simple hypothesis for this configuration can be devised. First, for the Ge1 site with its tetrahedral configuration, one would follow the octet rule, with an electron pair associated with each of its s and p valence orbitals. The remaining Ga/Ge atoms are closely associated with the Mo and Fe atoms.

In the case of these transition metals, the 18-*n* rule states that they will each need 18-*n* electrons to optimize their bonding, where *n* is the number of electron pairs they share with other transition metal atoms. The Fe atoms are isolated in (Ga/Ge)_6_ octahedra (*n* = 0), they are predicted to need 18 electrons each. The Mo atoms, on the other hand, are arranged in Mo_4_ tetrahedra with the bridging of the Mo–Mo contacts by Ga/Ge atoms setting up the formation of isolobal bonds.^88^ For the Mo atoms, then, *n* = 3. From these considerations, we arrive at an ideal electron count for the phase of (15 electrons/Mo atom) × (4 Mo atoms) + (18 electrons/Fe atom) × (1 Fe atoms) + (8 electrons/Ge1 site) × (1 Ge1 site) = 86 electrons per formula unit.

In this way, the application of the 18-*n* and octet schemes appears to account for the structure’s band gap. The reversed approximation Molecular Orbital (raMO) approach^22,23^ allows us to test this proposal against the wavefunctions of the compound. In this method, the occupied wavefunctions of a system are used as a basis set for the reproduction of a series of target functions, with each reconstructed raMO function, corresponding to one electron pair in the system, being removed from the basis set used for the subsequent steps.

The application of this process to the Mo_4_FeGa_16_Ge model (with the Fermi energy adjusted to lie in the band gap) is presented in Figure 4. The wavefunctions derived from the 2 × 2 × 2 k-point mesh corresponds to periodic boundary conditions with a 2 × 2 × 2 supercell containing 32 formula units. We begin the raMO procedure by reconstructing the Ge 4s orbitals for the 32 Ge atoms in the supercell, followed by the 4p_*x*_, 4p_*y*_, and 4p_*z*_ orbitals of these atoms to recreate filled octets on these atoms. The relative localization of these functions to the corresponding Ge atoms is tracked with the *P*_sphere_ values^58^ for the raMOs (bottom panel), which are defined as the fraction of the electrons within each raMO that is contained within a sphere of a specified radius (*r*_sphere_; see the Section 2.5 for specific values) around the target function’s central point. We also show isosurface plots for representative raMO functions.

**Figure 4:**
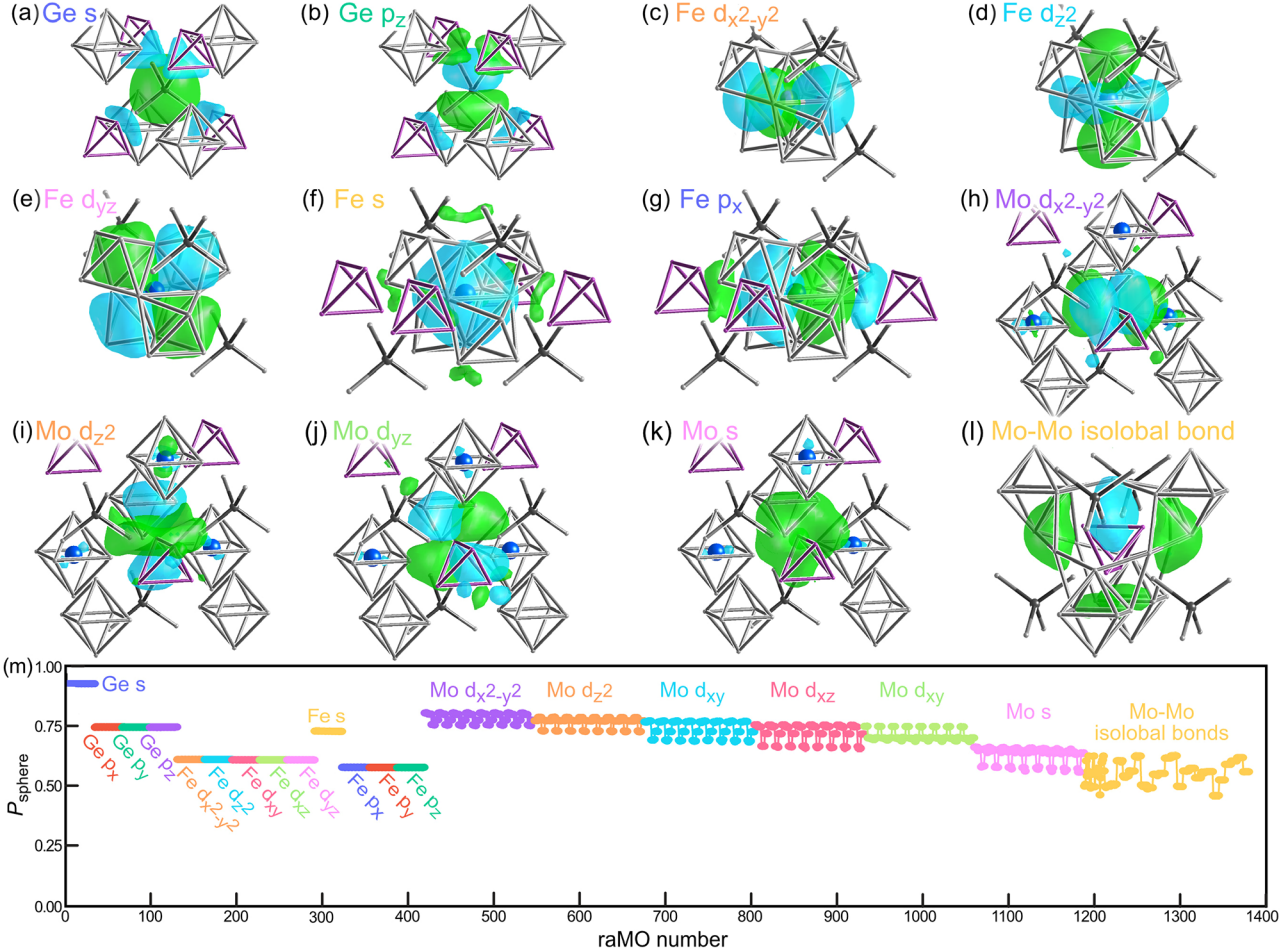
DFT-raMO analysis of a dumbbell-free model of Mo_4_FeGa_17.25–*x*_Ge_*x*_, drawing on all wavefunctions below the 86 electron band gap. (a–l) Isosurfaces for representative raMO constructions of the various functions hypothesized to host electron pairs in the structure from the application of the 18-*n* and octet bonding schemes. (m) *P*_sphere_ values for the sequence of raMO reconstructions of the 1376 electron pairs expected for a 2 × 2 × 2 supercell. The consistent values *P*_sphere_ across each orbital type, as opposed to a degradation from the beginning to the end, points to each of the proposed functions being fully occupied.

For both the Ge 4s and Ge 4p functions, the *P*_sphere_ values show highly consistent values across the sequences, with *P*_sphere_ > 0.9 for all 32 Ge 4s-centered raMOs in the supercell, and *P*_sphere_ > 0.7 for all 96 Ge 4p-centered raMOs. An examination of their orbital character shows that the functions are centered by the appropriate Ge s or p orbital, with covalent contributions from the Ga p orbitals in the tetrahedral coordination environment. These results point to complete octets on the Ge1 sites.

We next proceed through the Fe d, s, and p orbitals (following the order of the atomic orbital energies). Here, the *P*_sphere_ values again show strong consistency. The raMO plots also exhibit the expected nodal character, as well as a shift from predominately Fe character for the d-based functions to more notable bonding contributions from the neighboring Ga atoms in the Fe p-centered functions. Overall, the reconstruction of the Fe 18-electron configuration appears to be successful.

The remaining electrons in our bonding scheme are all on the Mo atoms. At this point, the procedure becomes more complicated as we expect isolobal bonds to arise along the edges of the Mo tetrahedra. Here, we assumed that electron pairs associated with individual Mo atoms can be constructed for the Mo 4d and 5s orbitals, while the Mo 5p would be most poised for interactions between the Mo centers. The Mo 4d and 5s reconstructions proceed smoothly, with *P*_sphere_ values consistently above those of the Fe d raMOs and the expected nodal character being evident in the isosurface plots. Some minor out-of-phase contributions from the Fe atoms may be seen in some of these functions, which we attribute to orthogonalization effects rather than true chemical interactions.

Finally, for each isolobal Mo–Mo bond, we attempt the reconstruction of a function consisting of in-phase sp hybrid orbitals from all atoms within 1.6 Å of the midpoint of the Mo pair. Here the *P*_sphere_ values show a little fluctuation, as may be expected from the decreasing size of the basis set of available wavefunctions, but never drop far below 0.5. A representative of these isolobal bond functions is presented in Figure 4l. While these functions are more diffuse than the earlier members of the sequence, the presence of a lobe along the Mo–Mo contact with similarly sized lobes of opposite phase on the back sides of the interaction is suggestive of an in-phase Mo p-Mo p combination. The lobes encompass the bridging Ga atoms and those on the other sides of the Mo centers, highlighting the multicenter character of the interaction. This set of Mo–Mo isolobal bonds completes the 18 electron configurations on the Mo atoms.

Altogether, the raMO analysis presented in Figure 4 accounts for all of the crystal orbitals below the band gap for the dumbbell-free model of Mo_4_FeGa_17.25–*x*_Ge_*x*_. This band gap simply arises from the filling of octadecets on the Mo and Fe atoms, with the support of Mo–Mo isolobal bonds, as well as a classical octet on the Ge1 site, whose orbitals chiefly interact with other E atoms. These conclusions can be summarized with the 18 - *n* + *m* bonding scheme for T-E intermetallics for the valence electron count per T atom, where *n* is the average number of isolobal bonds the T atoms participate in and *m* is the number of electrons per T atom on purely E-based orbitals. The 5 T atoms (Mo and Fe) in the formula unit have an average of *n* = 12/5 isolobal bonds. Meanwhile, the 8 electrons on Ge1 for 5 T atoms per formula unit gives *m* = 8/5. The ideal electron concentration is then 18–12/5 + 8/5 = 17.2 electrons per T atom.

### 3.5 Atom/Dumbbell Substitution in Mo_4_FeGa_17.25–*x*_Ge_*x*_

So far, we have focused in our theoretical analysis on a dumbbell-free model. To see how the incorporation of (Ga/Ge)_2_ dumbbells affects the bonding, we performed calculations on a model (using VASP with PAW–PW91 atomic potentials) in which one Ga2 site per unit cell is replaced with a Ga_2_ dumbbell, leading to the formula Mo_4_FeGa_16.25_Ge_1_.

The geometrical optimization of dumbbell-containing structures confirms features of our structure model refined using single crystal X-ray diffraction data (Figure 5). In the optimized structure, the Ga4–Ga4 dumbbell distance is 2.555 Å, compared to 2.592 Å in our refined crystal structure. Additionally, the Fe atoms at the centers of nearby (Ga1)_6_ octahedra are drawn toward these Ga4 atoms, essentially expanding their coordination environment. The Fe1–Ga4 distance now sits at 2.7336 Å, vs 2.8760 Å in the experimental model. In addition, the nearby Ga1 sites exhibit displacements to accommodate the addition of the Ga4 atoms to the Fe1 coordination environments, with the final positions resembling closely the Ga1b positions in the experimental model.

**Figure 5:**
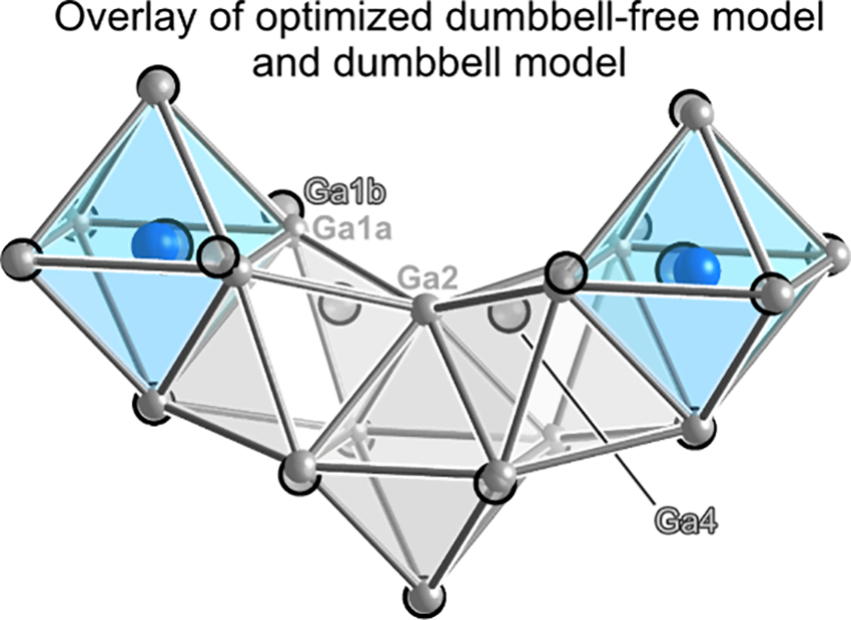
Comparison of the geometrical details of the PW91-DFT optimized atomic positions of the dumbbell-free and dumbbell-containing models of Mo_4_FeGa_17.25–*x*_Ge_*x*_. Solid spheres: positions for a dumbbell-free model with composition Mo_4_FeGa_16_Ge. Partially transparent spheres with black outlines: the corresponding positions in a model containing one dumbbell per conventional unit cell, with composition Mo_4_FeGa_16.25_Ge.

These geometrical correspondences support the physical realism of the proposed structural model. Additional confirmation can be found in the energetics from the DFT calculations. Using the total energies for dumbbell-free and dumbbell containing structures, as well as that of elemental Ga, we determine the energy for the incorporation of a Ga atom to form a dumbbell, following the reaction 4Mo_4_FeGa_16_Ge_1_(s) + Ga(s) → 4Mo_4_FeGa_16.25_Ge_1_(s), to be −0.52 eV. In other words, at T = 0 K there is an energetic driving force for the incorporation of one dumbbell per cell, when elemental Ga is used as a Ga source (the degree of stabilization would likely go down were the Ga to be drawn from a binary or ternary gallide). Above this temperature, the configurational entropy provided by the large number substitution patterns would further lower the free energy of the compound.

One rationale for the favorability of the dumbbell insertion may be derived from the DOS distribution for the dumbbell containing model (Figure 3b). Its overall shape, including the presence of the band gap near the *E*_F_, is nearly indistinguishable from that of the dumbbell-free model. However, the placement of the *E*_F_ relative to the bandgap has changed slightly. Whereas for the dumbbell-free model the *E*_F_ lies right on a peak just below the bandgap, for the model containing the dumbbell, the *E*_F_ has moved to the upper shoulder of this peak, closer to the bandgap. The addition of one Ga atom per unit cell, with its 3 valence electrons appears to have contributed to reducing the electron deficiency of the compound. Indeed, combining dumbbell substitution and Ge/Ga substitution, a formula that meets the ideal 86 electron count can be devised: Mo_4_FeGa_15_Ge_2.25_. However, the potential phase width of this compound remains to be determined, and experimental electric transport or band gap measurements are needed to confirm whether such a composition can be reached.

### 3.6 Chemical Pressures and Potential Properties of Mo_4_FeGa_17.25–*x*_Ge_*x*_

The bonding analysis in the previous sections highlighted Mo_4_FeGa_17.25–*x*_Ge_*x*_’s electronic band gap, the origin of this gap in simple bonding schemes, and the roles that Ge incorporation and atom/dumbbell substitution may play in placing the *E*_F_ relative to the gap. The overall architecture of the structure also sets the stage for intriguing atomic packing effects. Frameworks of face-sharing octahedra, a prominent feature of the Ti_2_Ni parent structure, have previously been explored using DFT-Chemical Pressure (CP) analysis.^36^ This theoretical approach provides a window into the atomic packing frustration that can arise within solid state structures, resolving the overall internal pressure of phase into a sum of competing interatomic pressures pushing toward the expansion (positive CP) and contraction (negative CP) of the structure, which represent overly short or overly long interatomic contacts, respectively.^32–35,64^ Such analysis has revealed that networks of face-sharing octahedra can relieve CPs associated with difficult-to-pack polyhedra by serving as structural buffers. Does this structural feature play a similar role in Mo_4_FeGa_17.25–*x*_Ge_*x*_?

In Figure 6a, we present the CP scheme calculated for a dumbbell-free model with composition Mo_4_FeGa_16_Ge. While the arrangement of positive and negative pressure features (indicated with white and black lobes, respectively) is complicated, it can be largely understood in terms of loci of positive CPs acting against a background of negative CP. For example, the most striking features here are positive pressures along the Ga–Ge contacts in the Ge@Ga_4_ tetrahedra, which push for longer Ge–Ga distances. Expansion here, however, is opposed by a matrix of negative pressure in Ga–Ga interactions that wrap around the exterior of the tetrahedron. Similarly, the Fe atoms at the centers of the (Ga1)_6_ octahedra represent another focal point of positive pressure, exhibiting an octahedral distribution of white CP lobes toward their Ga neighbors. This desired expansion is again resisted by negative pressures within the Ga sublattice, particularly along the very edges of those octahedra.

**Figure 6:**
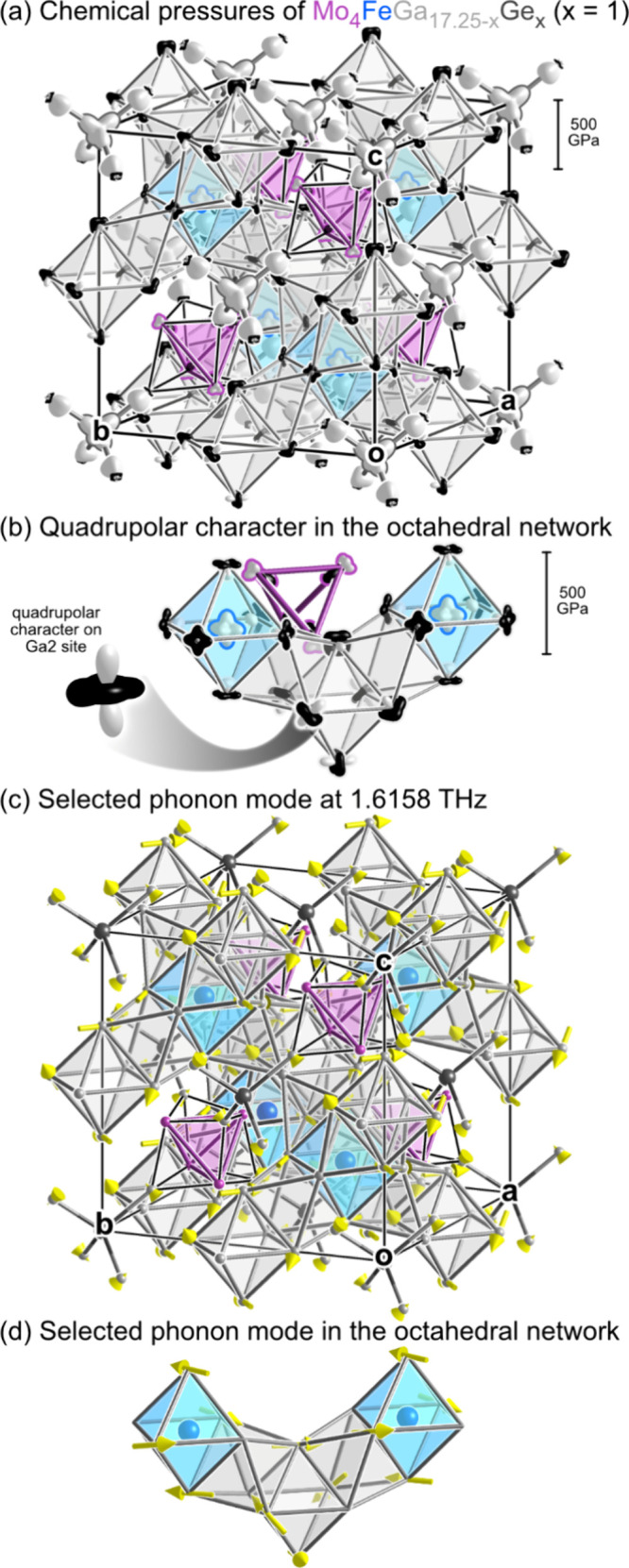
DFT-Chemical pressure analysis of a dumbbell-free model of (a) the unit cell of Mo_4_FeGa_17.25–*x*_Ge_*x*_ (modeled with *x* = 1) with (b) close up view of the quadrupolar character of the CP distributions of atoms making up the octahedral network. (c, d) The negative CP features of these quadrupoles correlate with the atomic displacements in low frequency phonon modes at Γ, as depicted here with one example.

Finally, the only remaining positive CPs within the structure are associated with the Mo_4_ tetrahedra, which are compressed by contacts from the surrounding Ga atoms in the octahedral framework. The Mo atoms exhibit Mo–Mo negative pressures on their opposite sides, creating a CP dipole which would seem to indicate that the Mo_4_ tetrahedron as whole would be stabilized by contraction. As the structure has, in fact, been fully geometrically optimized, this effect likely stems from some of the positive CPs associated with the Mo–Mo interactions being mapped to the Mo–Ga interactions. The existence of Mo–Mo positive pressures is supported by the high frequency phonon mode (6.44 THz) at the Γ-point associated with the compression of the Mo tetrahedron (see mode 51 in the Supporting Information).

Aside from these CPs emanating from the Ge@Ga_4_, Fe@Ga_6_, and Mo_4_ units, the major features in the CP scheme are negative CPs that are mainly distributed diffusely among the Ga–Ga interactions, including the Ga octahedral framework. Indeed, no noticeable positive CP arises between atoms of these octahedra. This result is consistent with the octahedral framework playing the role of a host matrix that stretches or contracts as needed to accommodate its occupants.

This organization of the CP scheme into positive pressures oriented radially from distinct centers has consequences for the vibrational modes of the structure (Figures 6b-d). Negative CPs tend to arise in the perpendicular directions, running tangentially to the Ga atoms’ contacts to the Ge, Fe, or Mo atoms. In the process, the CP distributions of these Ga atoms acquire quadrupolar (d-orbital-shaped) character. Such features are associated with anisotropic vibrations, in which displacements along the directions of positive CP face significantly more resistance than those along negative CPs (which would both lengthen interactions with positive CP and contract others with negative CP).^89^ These expectations are reflected in the phonon band structure. For example, the lowest frequency optical modes at Γ (Figure 6c,d) involve exclusively Ga atoms, which move along directions perpendicular to their directions of positive CP. The soft motions associated with these CP quadrupoles hint that vibrations play a part in the anisotropic shapes of the thermal ellipsoids on the Ga sites in the refined crystal structure.

The CP quadrupoles, however, also offer an account of more conspicuous aspects of our structure models for this compound, its single atom/dumbbell disorder and (at T = 100 K) split sites. The Ga2 site has the most well-developed CP quadrupole, as it experiences positive CP from interactions with Mo atoms on opposite sides (rather than positive CP from predominantly one side). This arrangement sets the stage for soft motion in the perpendicular directions. One of these motions is represented with high amplitudes in the lowest optical mode at Γ, with the Ga2 atoms moving back and forth toward Fe atoms in nearby octahedra (Figure 6d).

The softness of this mode can be attributed to the combined effect of (1) the squeezing of the Ga atom between the two Mo atoms at the average positions and (2) the sparse atomic packing in the interstitial spaces adjacent to the Fe atoms. The replacement of an atom at a Ga2 site with a dumbbell whose atoms occupy a pair of Ga4 positions has advantages for both aspects of the CP scheme. The removal of a Ga2 atom is poised to relieve the positive Mo–Ga CPs, while the inclusion of atoms at the Ga4 positions increases the packing density in a region with Ga–Ga negative CPs. The occupation of the Ga4 site has an added benefit to the nearby Fe atoms. The positive CPs of the Fe atoms indicate that they are somewhat too large for their coordination environments. These environments are expanded by the inclusion of the Ga4 atom, with the displacement of the Ga1 sites already connected to the Fe being supported by their CP quadrupoles. Indeed, in the optimization of the dumbbell containing structure model (Figure 5), the Fe atoms show a significant displacement toward the nascent Ga4 atoms, illustrating their inclusion in the Fe coordination environment.

In this way, the CP features of Mo_4_FeGa_17.25–*x*_Ge_*x*_ highlight a complementarity between atomic packing and electronic effects in the single atom/dumbbell substitution at the Ga2 sites. In terms of the electronic factor, the substitution offers a mechanism to add electrons to the band structure, moving the *E*_F_ closer to the band gap corresponding to completion of the 18-*n* and octet bonding schemes. From the packing perspective, the Ga sublattice has the flexibility to accept the new Ga atom, while the Fe and Mo atoms benefit from the expanded coordination environments.

## 4 Conclusions

The exploration of intermetallic materials involves a tension between a desire for useful guiding principles and the surety of unexpected discoveries from the endless variety of competing structures in any given system. Our discovery of Mo_4_FeGa_17.25–*x*_Ge_*x*_ captures this tension, as the compound emerged from a fortuitous combination of these four elements under conditions where a gallide was unexpected (and indeed no gallide appears to be present at the temperatures used without the inclusion of Ge). Whereas our original synthesis aimed at the formation of a structure with intergrowth motifs, Fe_18_Mo_26_Ge_9_, the result was an intergrowth of a different sort, in which tetrahedral units familiar from Zintl phases are combined with stella quadrangula often associated with polar intermetallics. The integration of these units into a single structure appears to be facilitated by a framework of face-sharing octahedra, which offers a flexible matrix that accommodates the atomic packing needs of its contents.

The structure of Mo_4_FeGa_17.25–*x*_Ge_*x*_ also yields lessons on the driving forces shaping point substitutions and insertions in solid state structures. The Fe atoms lie in Ga octahedra, resembling the transition metal (T) atoms in stuffed AuCu_3_-type phases of the form R_4_TGa_12_ (R = lanthanide or similarly electropositive element).^8,9^ The ability of Ga octahedra to host T atoms is potentially supported in Mo_4_FeGa_17.25–*x*_Ge_*x*_ by the flexibility created by arranging the octahedra into a face-sharing network.

The structure also differs from the Ti_2_Ni-type prototype by the replacement of a transition metal tetrahedron with a single atom, creating a pattern of Mo_4_-centered stella quadrangula and Ge@Ga_4_ tetrahedra linked through vertex sharing. The strong positive CPs acting on the Ge atoms hint that atomic packing tensions influence this substitution; the placement of a larger atom here would tend to destabilize the structure. It is also notable that the CP features around this Ge site recall the original tetrahedron in the Ti_2_Ni type: one simply imagines placing at atom opposite to each of the Ge’s positive CP lobes, in a position that would soothe negative CPs on the neighboring Ga atoms. This notion of substitution templated by CP features extends to the single atom/dumbbell substitution at the Ga2 site, where the substitution depopulates a position with positive CP and populates positions of negative CP. Combining these observations with similar substitutions in the BaAl_4_,^2,34^ CaCu_5_,^90^ and Heusler structure^7^ types suggests a principle of complementary cluster substitution, in which the replacement of one group of atoms with another can be facile when the atoms removed are subject to positive CP and the atoms added serve to satisfy negative CPs.

While our focus in this Article has been the structural features and bonding in Mo_4_FeGa_17.25–*x*_Ge_*x*_, we note that the results build a case for the exploration of Mo_4_FeGa_17.25–*x*_Ge_*x*_’s physical properties. The band structure calculations reveal a band gap near the *E*_F_, with the placement of the *E*_F_ being sensitive to the Ga/Ge ratio and the degree of dumbbell incorporation. As such, Mo_4_FeGa_17.25–*x*_Ge_*x*_ has the potential to be a tunable semiconductor if it exhibits a phase width. At the same time, the CP scheme foretells relatively rigid units, such as FeGa_6_ octahedra and Ge@Ga_4_ tetrahedra, embedded in a flexible medium provided by the structure’s framework of face-sharing tetrahedra. Qualitatively, this arrangement would seem well suited for low thermal conductivity, which could be augmented by the single atom/dumbbell disorder. Together, these electronic and atomic packing aspects align with the phonon-glass/electron-crystal scheme for thermoelectric materials,^37^ calling for the investigation of its physical properties.

## Supplementary Material



## Notes

Additional crystallographic details, including refined atomic coordinates, atomic displacement parameters, and selected interatomic distances; SEM-BSE images; WDS data points; parameters regarding the DFT calculations; DFT-optimized geometries and total energies, additional electronic DOS curves; phonon band structure and Γ point optical phonon modes for the dumbbell-free model of the title compound (PDF)

## †

Institut für Materialwissenschaft, Technische Universität Darmstadt, Darmstadt 64287, Germany

## ‡

Yusuf Hamied Department of Chemistry, University of Cambridge, Cambridge CB2 1EW, United Kingdom.
