# Supplementary material for: Mo4FeGa17.25– x Ge x : Complementary Point Substitutions, Buffering Frameworks, and Merging of the 18‑n and Octet Bonding Schemes
Source: Chem Mater. 2026 May 3;38(10):4975–87. doi: 10.1021/acs.chemmater.5c03434 (PMC13217558; doi:10.1021/acs.chemmater.5c03434)

# Supporting Information

## $Mo_4FeGa_{17.25-x}Ge_x$: Complementary Point Substitutions, Buffering Frameworks, and Merging of the 18-*n* and Octet Bonding Schemes

**Danica G. Gressel, Patrick K. Cross, Jonathan S. Van Buskirk, and Daniel C. Fredrickson***

*Department of Chemistry, University of Wisconsin-Madison,*

*1101 University Avenue, Madison, Wisconsin 53706, United States.*

* Corresponding Author, e-mail: danny@chem.wisc.edu

## S1. Crystallographic Data Tables for $Mo_4FeGa_{17.25-x}Ge_x$ (T = 298 K)

**Table S1. Refined Atomic Coordinates for $Mo_4FeGa_{17.25-x}Ge_x$ at T = 298 K.**

| Site | Wyckoff | *x* | *y* | *z* | $U_{equiv}$ (Å$^2$) | Occupancy |
|---|---|---|---|---|---|---|
| Mo1 | 16*e* | 0.654201(19) | 0.845799(19) | 0.845799(19) | 0.00636(5) | 1 |
| Ge1 | 4*a* | 0 | 0 | 0 | 0.00790(10) | 1 |
| Ga3 | 16e | 0.87867(3) | 0.87867(3) | 0.87867(3) | 0.00907(6) | 1 |
| Ga1 | 24*g* | 0.45263(5) | ¾ | ¾ | 0.02002(12) | 1 |
| Fe3 | 4*c* | ¼ | ¾ | ¾ | 0.01142(16) | 1 |
| Ga2 | 24*f* | 0.31747(5) | 0 | 0 | 0.01625(11) | 0.9587(18) |
| Ga4 | 48*h* | 0.3141(8) | 0.0795(6) | 0.0795(6) | 0.0142(18) | 0.0413(18) |

**Table S2. Refined Atomic Displacement Parameters for $Mo_4FeGa_{17.25-x}Ge_x$ at 298 K.**

| Site | $U_{11}$ (Å$^2$) | $U_{22}$ (Å$^2$) | $U_{33}$ (Å$^2$) | $U_{12}$ (Å$^2$) | $U_{13}$ (Å$^2$) | $U_{23}$ (Å$^2$) |
|---|---|---|---|---|---|---|
| Mo1 | 0.00636(8) | 0.00636(8) | 0.00636(8) | 0.00019(7) | 0.00019(7) | -0.00019(7) |
| Ge1 | 0.00790(17) | 0.00790(17) | 0.00790(17) | 0 | 0 | 0 |
| Ga3 | 0.00907(11) | 0.00907(11) | 0.00907(11) | -0.00102(10) | -0.00102(10) | -0.00102(10) |
| Ga1 | 0.0102(2) | 0.02495(19) | 0.02495(19) | 0 | 0 | 0.0071(2) |
| Fe3 | 0.0114(3) | 0.0114(3) | 0.0114(3) | 0 | 0 | 0 |
| Ga2 | 0.0143(2) | 0.01723(16) | 0.01723(16) | 0 | 0 | 0.0103(2) |
| Ga4 | 0.015(4) | 0.014(3) | 0.014(3) | 0.008(2) | 0.008(2) | 0.005(3) |

**Table S3. Selected Interatomic Distances for $Mo_4FeGa_{17.25-x}Ge_x$ at 298 K.**

| Site | Neighbor | Distance (Å) |
|---|---|---|
| Mo1 | Ga3 (×3) | 2.6424(8) |
| | Ga1 (× 3) | 2.7996(8) |
| | Ga2 (×3) | 2.5349(5) |
| | Ga4 (×6) | 2.852(7) |
| Ge1 | Ga3 (×4) | 2.4225(5) |
| Ga3 | Ga1 (×3) | 2.8603(7) |
| | Ga2 (×3) | 3.0040(7) |
| Ga1 | Ga2 (×4) | 3.0344(7) |
| | Fe1 | 2.3358(9) |
| | Ga4 (×4) | 2.132(7) |
| | Ga4 (×2) | 3.206(7) |
| Ga2 | Ga2 (×4) | 2.9755(8) |
| | Ga4 (×4) | 2.616(9) |
| Fe1 | Ga4 (×12) | 2.876(7) |
| Ga4 | Ga4 | 2.591(9) |
| | Ga4 (×2) | 1.736(11) |

## S2. Crystallographic Data Tables for $Mo_4FeGa_{17.25-x}Ge_x$ (T = 100 K)

**Table S4. Crystal Data for $Mo_4FeGa_{17.25-x}Ge_x$ at 100 K.**

| | |
|---|---|
| Refined Composition | $Mo_4Fe(Ga/Ge)_{17.48(7)}$ |
| WDS Composition | $Mo_{4.0(4)}Fe_{1.0(1)}Ga_{15.4(9)}Ge_{2.1(4)}$ |
| Crystal Dimension (mm$^3$) | 0.020 x 0.035 x 0.020 |
| Crystal Color | Dark gray, metallic sheen |
| Data Collection Temperature | 100 K |
| Radiation Source, λ (Å) | Mo Kα, 0.71073 |
| Absorption Correction | Multi-scan |
| Space Group | $F\bar{4}3m$ (No. 216) |
| *a* (Å) | 11.5131(19) |
| Cell volume (Å$^3$) | 1,526.1(4) |
| *Z* | 4 |
| Absorption Coefficient (mm$^{-1}$) | 34.511 |
| $\theta_{min}$, $\theta_{max}$ | 3.06, 45.17 |
| Refinement Method | $F^2$ |
| $R_{int}$ (obs., all) | 0.0444, 0.0446 |
| Number of Reflections | 10,160 |
| Number of Parameters | 28 |
| Unique Reflections (I>3σ, all) | 667, 682 |
| R(I>3σ), $R_w$(I>3σ) | 0.0207, 0.0214 |
| R(all), $R_w$(all) | 0.0509, 0.0511 |
| S(I>3σ), S(all) | 1.51, 1.53 |
| $\Delta\rho_{max}$, $\Delta\rho_{min}$ ($e^-$/Å$^3$) | 1.24, -1.60 |

**Table S5. Refined Atomic Coordinates for $Mo_4FeGa_{17.25-x}Ge_x$ at 100 K.**

| Site | Wyckoff | *x* | *y* | *z* | $U_{equiv}$ (Å$^2$) | Occupancy |
|---|---|---|---|---|---|---|
| Mo1 | 16*e* | 0.34581(2) | 0.15419(2) | 0.15419(2) | 0.00385(4) | 1 |
| Ge1 | 4*a* | 0 | 0 | 0 | 0.00402(15) | 1 |
| Ga3 | 16e | 0.12130(3) | 0.12130(3) | 0.12130(3) | 0.00475(5) | 1 |
| Fe1 | 4*c* | ¾ | ¼ | ¼ | 0.00944(15) | 1 |
| Ga2 | 24*f* | 0.31751(5) | 0 | 0 | 0.00981(9) | 0.957(2) |
| Ga4 | 48*h* | 0.3132(7) | 0.9206(5) | 0.0794(5) | 0.0058(13) | 0.043(2) |
| Ga1a | 24*g* | 0.54761(6) | ¼ | ¼ | 0.01124(12) | 0.962(2) |
| Ga1b | 48*h* | 0.5430(10) | 0.2852(11) | 0.2852(11) | 0.01124(12) | 0.038(2) |

**Table S6. Refined Atomic Displacement Parameters for $Mo_4FeGa_{17.25-x}Ge_x$ at 100 K.**

| Site | $U_{11}$ (Å$^2$) | $U_{22}$ (Å$^2$) | $U_{33}$ (Å$^2$) | $U_{12}$ (Å$^2$) | $U_{13}$ (Å$^2$) | $U_{23}$ (Å$^2$) |
|---|---|---|---|---|---|---|
| Mo1 | 0.00385(6) | 0.00385(6) | 0.00385(6) | -0.00001(6) | -0.00001(6) | 0.00001(6) |
| Ga3 | 0.00475(9) | 0.00475(9) | 0.00475(9) | -0.00040(9) | -0.00040(9) | -0.00040(9) |
| Fe1 | 0.0094(3) | 0.0094(3) | 0.0094(3) | 0 | 0 | 0 |
| Ga2 | 0.00670(19) | 0.01136(15) | 0.01136(15) | 0 | 0 | -0.00740(18) |
| Ga4 | 0.005(3) | 0.006(2) | 0.006(2) | 0.0004(15) | -0.0004(15) | -0.003(2) |
| Ga1a | 0.0066(2) | 0.0135(2) | 0.0135(2) | 0 | 0 | 0.0042(3) |
| Ga1b | 0.0066(2) | 0.0135(2) | 0.0135(2) | 0 | 0 | 0.0042(3) |

**Table S7. Selected Interatomic Distances for $Mo_4FeGa_{17.25-x}Ge_x$ at 100 K.**

| Site | Neighbor | Distance (Å) |
|---|---|---|
| Mo1 | Ga3 (×3) | 2.6397(9) |
| | Ga1a (× 3) | 2.7984(9) |
| | Ga2 (×3) | 2.5315(6) |
| | Ga4 (×6) | 2.849(6) |
| | Ga1b (×3) | 2.475(11) |
| Ge1 | Ga3 (×4) | 2.4189(6) |
| Ga3 | Ga1a (×3) | 2.8587(8) |
| | Ga2 (×3) | 3.0006(8) |
| | Ga5 (×6) | 2.881(12) |
| Ga1a | Ga2 (×4) | 3.0313(9) |
| | Fe1 | 2.3302(10) |
| | Ga4 (×4) | 2.126(6) |
| | Ga4 (×2) | 3.207(6) |
| | Ga1b (×4) | 3.089(12) |
| Ga2 | Ga2 (×4) | 2.9713(9) |
| | Ga4 (×4) | 2.621(7) |
| | Ga4 (×2) | 1.294(6) |
| | Ga1b (×4) | 2.545(12) |
| Fe1 | Ga4 (×12) | 2.872(6) |
| | Ga1b (×12) | 2.453(11) |
| Ga4 | Ga4 | 2.585(8) |
| | Ga4 (×2) | 1.749(10) |
| | Ga1b | 1.643(13) |
| | Ga1b | 2.664(14) |
| | Ga1b | 2.754(14) |
| | Ga1b | 1.643(13) |
| | Ga1b | 2.664(14) |
| Ga1b | Ga1b | 1.156(17) |
| | Ga1b (×2) | 2.794(16) |

## S3. WDS Data for $Mo_4FeGa_{17.25-x}Ge_x$

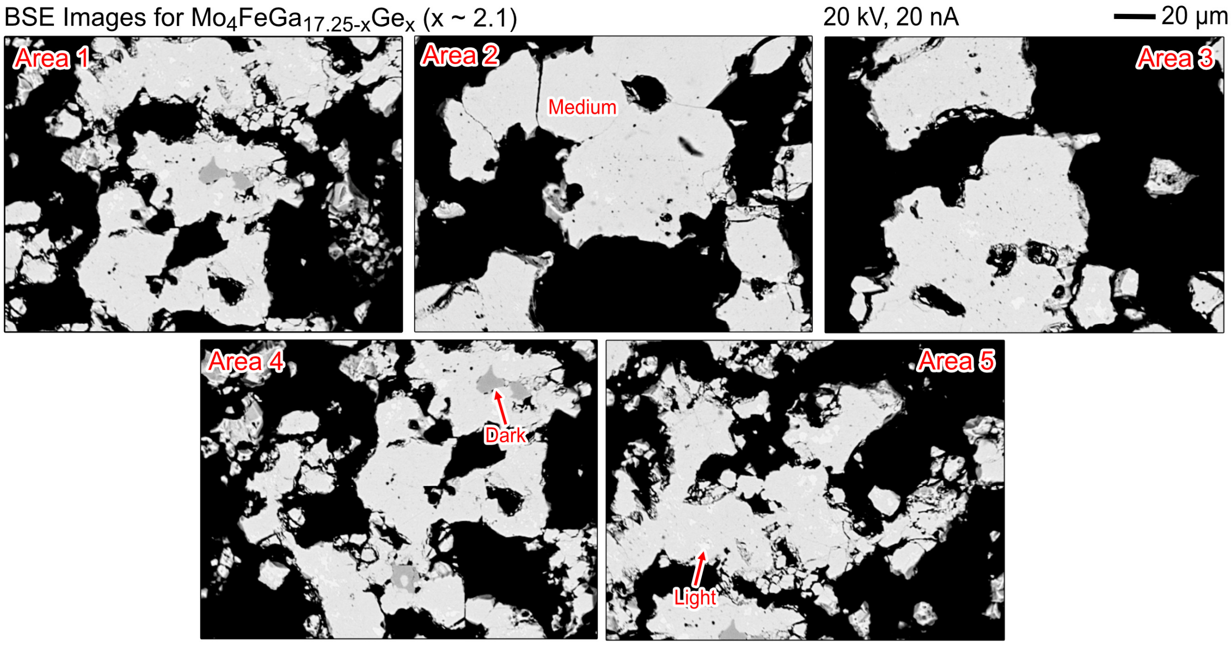


**Figure S1.** Scanning Electron Microscope-Back-Scattered Electron (SEM-BSE) images of polished sections of a sample containing $Mo_4FeGa_{17.25-x}Ge_x$.

**Table S8. WDS Compositions for Different Points on the Main Phase of the $Mo_4FeGa_{17.25-x}Ge_x$ Sample.**

| Area | Point Number | Mo AT% | Fe AT% | Ga AT% | Ge AT% | TOTAL |
|---|---|---|---|---|---|---|
| area1 mediumBSE phase | 436 | 17.4819 | 4.83724 | 68.9272 | 8.75373 | 100 |
| area1 mediumBSE phase | 437 | 17.3565 | 4.74536 | 69.08 | 8.81818 | 100 |
| area1 mediumBSE phase | 438 | 17.6679 | 4.66382 | 68.8348 | 8.83346 | 100 |
| area1 mediumBSE phase | 439 | 17.2547 | 4.84781 | 69.3571 | 8.54044 | 100 |
| area1 mediumBSE phase | 440 | 17.6401 | 4.70554 | 68.8262 | 8.82812 | 100 |
| area1 mediumBSE phase | 441 | 17.3012 | 4.58946 | 69.0371 | 9.07222 | 100 |
| area1 mediumBSE phase | 442 | 17.5102 | 4.58149 | 69.0948 | 8.81351 | 100 |
| area1 mediumBSE phase | 443 | 17.3472 | 4.70311 | 69.1316 | 8.81807 | 100 |
| area1 mediumBSE phase | 444 | 17.8245 | 4.53406 | 68.6192 | 9.02227 | 100 |
| area1 mediumBSE phase | 445 | 16.5035 | 4.55751 | 70.7574 | 8.18156 | 100 |
| area2 mediumBSE phase | 446 | 16.5436 | 4.445 | 70.7961 | 8.21536 | 100 |
| area2 mediumBSE phase | 447 | 16.9846 | 4.62491 | 69.7573 | 8.63322 | 100 |
| area2 mediumBSE phase | 448 | 19.3439 | 4.13325 | 65.8877 | 10.6351 | 100 |
| area2 mediumBSE phase | 449 | 16.9684 | 4.67216 | 69.6558 | 8.70369 | 100 |
| area2 mediumBSE phase | 450 | 17.4205 | 4.70316 | 68.973 | 8.90339 | 100 |
| area2 mediumBSE phase | 451 | 17.2025 | 4.75474 | 69.3476 | 8.6952 | 100 |
| area2 mediumBSE phase | 452 | 18.0362 | 4.36486 | 67.8721 | 9.72678 | 100 |
| area2 mediumBSE phase | 453 | 17.1025 | 4.78028 | 69.3916 | 8.72568 | 100 |
| area2 mediumBSE phase | 454 | 16.0883 | 4.57858 | 71.0756 | 8.25761 | 100 |
| area2 mediumBSE phase | 455 | 18.4771 | 4.35337 | 67.3322 | 9.8373 | 100 |
| area3 mediumBSE phase | 456 | 17.1825 | 4.58933 | 69.7382 | 8.48995 | 100 |
| area3 mediumBSE phase | 457 | 17.0195 | 4.6338 | 69.7352 | 8.61149 | 100 |
| area3 mediumBSE phase | 458 | 17.5543 | 4.71656 | 68.9197 | 8.80936 | 100 |
| area3 mediumBSE phase | 459 | 16.3718 | 4.38592 | 71.294 | 7.9483 | 100 |

| | | | | | | |
|---|---|---|---|---|---|---|
| area3 mediumBSE phase | 460 | 16.2585 | 4.61881 | 70.7453 | 8.37744 | 100 |
| area3 mediumBSE phase | 461 | 17.9547 | 4.48077 | 68.3511 | 9.21346 | 100 |
| area3 mediumBSE phase | 462 | 17.3475 | 4.63066 | 69.1565 | 8.8653 | 100 |
| area3 mediumBSE phase | 463 | 17.1936 | 4.62168 | 69.4261 | 8.75865 | 100 |
| area3 mediumBSE phase | 464 | 17.257 | 4.68487 | 69.2569 | 8.80123 | 100 |
| area3 mediumBSE phase | 465 | 17.7351 | 4.63536 | 68.825 | 8.80461 | 100 |
| area4 mediumBSE phase | 466 | 17.189 | 4.71747 | 69.1671 | 8.92646 | 100 |
| area4 mediumBSE phase | 467 | 16.9535 | 4.59219 | 69.9112 | 8.54313 | 100 |
| area4 mediumBSE phase | 468 | 17.1174 | 4.67178 | 69.1376 | 9.07322 | 100 |
| area4 mediumBSE phase | 469 | 16.3732 | 4.79781 | 68.7187 | 10.1103 | 100 |
| area4 mediumBSE phase | 470 | 21.5357 | 3.55665 | 62.0688 | 12.8388 | 100 |
| area4 mediumBSE phase | 471 | 17.8349 | 4.61439 | 68.5387 | 9.01203 | 100 |
| area4 mediumBSE phase | 472 | 17.4666 | 4.56138 | 68.7446 | 9.22746 | 100 |
| area4 mediumBSE phase | 473 | 24.8806 | 2.09974 | 55.6045 | 17.4152 | 100 |
| area4 mediumBSE phase | 474 | 17.2646 | 4.69358 | 69.1705 | 8.87127 | 100 |
| area4 mediumBSE phase | 475 | 17.9371 | 4.43547 | 68.3868 | 9.24065 | 100 |
| area5 mediumBSE phase | 476 | 27.2597 | 1.72569 | 52.0557 | 18.9589 | 100 |
| area5 mediumBSE phase | 477 | 17.3634 | 4.61365 | 69.0294 | 8.99352 | 100 |
| area5 mediumBSE phase | 478 | 17.6099 | 4.63822 | 68.7947 | 8.95718 | 100 |
| area5 mediumBSE phase | 479 | 17.404 | 4.76611 | 68.9463 | 8.88357 | 100 |
| area5 mediumBSE phase | 480 | 16.7811 | 4.50763 | 70.3325 | 8.37874 | 100 |
| area5 mediumBSE phase | 481 | 17.0301 | 4.61831 | 69.608 | 8.74355 | 100 |
| area5 mediumBSE phase | 482 | 16.7759 | 4.55638 | 70.1868 | 8.48092 | 100 |
| area5 mediumBSE phase | 483 | 23.5775 | 2.63337 | 58.8093 | 14.9799 | 100 |
| area5 mediumBSE phase | 484 | 20.3216 | 3.47183 | 64.1125 | 12.0941 | 100 |
| area5 mediumBSE phase | 485 | 17.4515 | 4.55684 | 68.9859 | 9.00581 | 100 |
| Average of 50 points | | 17.92114 | 4.420039 | 68.15024 | 9.508588 | |
| Standard Deviation | | 2.11286 | 0.638044 | 3.63542 | 2.157863 | |

**Table S9. WDS Compositions for Different Points of the Bright BSE Phase in the $Mo_4FeGa_{17.25-x}Ge_x$ Sample.**

| Area | Line Number | Mo AT% | Fe AT% | Ga AT% | Ge AT% | TOTAL |
|---|---|---|---|---|---|---|
| area1 brightBSE phase | 426 | 30.2586 | 1.19406 | 47.5268 | 21.0205 | 100 |
| area1 brightBSE phase | 427 | 19.1144 | 4.0687 | 66.2423 | 10.5746 | 100 |
| area1 brightBSE phase | 428 | 32.0737 | 0.546597 | 44.7641 | 22.6156 | 100 |
| area1 brightBSE phase | 429 | 21.7678 | 3.20609 | 62.6668 | 12.3593 | 100 |
| area1 brightBSE phase | 430 | 30.1571 | 1.26954 | 48.2185 | 20.3548 | 100 |
| Average of 5 points | | 26.67432 | 2.056997 | 53.8837 | 17.38496 | |
| Standard Deviation | | 5.81714 | 1.501118 | 9.817709 | 5.500794 | |

**Table S10. WDS Compositions for Different Points of the Dark BSE Phase in the $Mo_4FeGa_{17.25-x}Ge_x$ Sample.**

| Area | Line Number | Mo AT% | Fe AT% | Ga AT% | Ge AT% | TOTAL |
|---|---|---|---|---|---|---|
| area1 darkBSE phase | 431 | 0.204942 | 24.0268 | 74.4905 | 1.27777 | 100 |
| area1 darkBSE phase | 432 | 0.006126 | 24.252 | 74.3006 | 1.4413 | 100 |
| area1 darkBSE phase | 433 | 0.014766 | 24.3909 | 74.2132 | 1.38119 | 100 |
| area1 darkBSE phase | 434 | 0.050897 | 23.9256 | 74.6878 | 1.33564 | 100 |
| area1 darkBSE phase | 435 | 0.297748 | 23.8698 | 74.6587 | 1.17378 | 100 |
| Average of 5 points | | 0.114896 | 24.09302 | 74.47016 | 1.32193 | |
| Standard Deviation | | 0.1299 | 0.2215 | 0.211014 | 0.10228 | |

## S4. Additional Details Regarding Electronic Structure Calculations

**Table S11. Parameters for VASP Calculations.**[a]

| Structure | Converged/Structure Optimization energy cutoff (eV) | Converged k-point grid | DOS k-point grid | FFT grid | Total Energy/cell (eV) |
|---|---|---|---|---|---|
| $Mo_4FeGa_{16}Ge$ | 225/300 | 3×3×3 | 15×15×15 | 120×120×120 | -428.80538 |
| $Mo_4FeGa_{16.25}Ge$ | 225/300 | 3×3×3 | 15×15×15 | 120×120×120 | -432.23058 |
| Ga | 200/260 | 7×7×7 | - | 48×48×48 | -11.64123 |

[a]All calculations were performed using the PAW-PW91 atomic potentials. The k-point grids are Γ centered.

**Table S12. Lattice Parameters for Optimized Structures from VASP.**

| Structure | *a* (Å) | *b* (Å) | *c* (Å) | α (°) | β (°) | γ (°) |
|---|---|---|---|---|---|---|
| $Mo_4FeGa_{16}Ge$ | 11.5785 | 11.5785 | 11.5785 | 90.000 | 90.000 | 90.000 |
| $Mo_4FeGa_{16.25}Ge$ | 11.5747 | 11.6401 | 11.5747 | 90.000 | 89.559 | 90.000 |
| Ga | 4.5022 | 4.5022 | 4.6218 | 120.000 | 90.000 | 90.000 |

**Table S13. Fractional Atomic Coordinates of Structures Optimized from VASP.**

| Structure | Site | *x* | *y* | *z* |
|---|---|---|---|---|
| $Mo_4FeGa_{16}Ge$ | Mo1 | 0.65501 | 0.84498 | 0.84498 |
| | Mo2 | 0.34498 | 0.15501 | 0.84498 |
| | Mo3 | 0.34498 | 0.84498 | 0.15501 |
| | Mo4 | 0.65501 | 0.15501 | 0.15501 |
| | Mo5 | 0.84498 | 0.65501 | 0.84498 |
| | Mo6 | 0.84498 | 0.34498 | 0.15501 |
| | Mo7 | 0.15501 | 0.34498 | 0.84498 |
| | Mo8 | 0.15501 | 0.65501 | 0.15501 |
| | Mo9 | 0.84498 | 0.84498 | 0.65501 |
| | Mo10 | 0.15501 | 0.84498 | 0.34498 |
| | Mo11 | 0.84498 | 0.15501 | 0.34498 |
| | Mo12 | 0.15501 | 0.15501 | 0.65501 |
| | Mo13 | 0.65501 | 0.34498 | 0.34498 |
| | Mo14 | 0.34498 | 0.65501 | 0.34498 |
| | Mo15 | 0.34498 | 0.34498 | 0.65501 |
| | Mo16 | 0.65501 | 0.65501 | 0.65501 |
| | Ge1 | 0.00000 | 0.00000 | 0.00000 |
| | Ge2 | 0.00000 | 0.50000 | 0.50000 |
| | Ge3 | 0.50000 | 0.00000 | 0.50000 |
| | Ge4 | 0.50000 | 0.50000 | 0.00000 |
| | Ga1 | 0.87893 | 0.87893 | 0.87893 |
| | Ga2 | 0.12106 | 0.12106 | 0.87893 |
| | Ga3 | 0.12106 | 0.87893 | 0.12106 |
| | Ga4 | 0.87893 | 0.12106 | 0.12106 |
| | Ga5 | 0.87893 | 0.37893 | 0.37893 |
| | Ga6 | 0.12106 | 0.62106 | 0.37893 |
| | Ga7 | 0.12106 | 0.37893 | 0.62106 |

| | | | |
|---|---|---|---|
| Ga8 | 0.87893 | 0.62106 | 0.62106 |
| Ga9 | 0.37893 | 0.87893 | 0.37893 |
| Ga10 | 0.62106 | 0.12106 | 0.37893 |
| Ga11 | 0.62106 | 0.87893 | 0.62106 |
| Ga12 | 0.37893 | 0.12106 | 0.62106 |
| Ga13 | 0.37893 | 0.37893 | 0.87893 |
| Ga14 | 0.62106 | 0.62106 | 0.87893 |
| Ga15 | 0.62106 | 0.37893 | 0.12106 |
| Ga16 | 0.37893 | 0.62106 | 0.12106 |
| Ga17 | 0.45058 | 0.75000 | 0.75000 |
| Ga18 | 0.54941 | 0.25000 | 0.75000 |
| Ga19 | 0.54941 | 0.75000 | 0.25000 |
| Ga20 | 0.45058 | 0.25000 | 0.25000 |
| Ga21 | 0.75000 | 0.45058 | 0.75000 |
| Ga22 | 0.75000 | 0.54941 | 0.25000 |
| Ga23 | 0.25000 | 0.54941 | 0.75000 |
| Ga24 | 0.25000 | 0.45058 | 0.25000 |
| Ga25 | 0.75000 | 0.75000 | 0.45058 |
| Ga26 | 0.25000 | 0.75000 | 0.54941 |
| Ga27 | 0.75000 | 0.25000 | 0.54941 |
| Ga28 | 0.25000 | 0.25000 | 0.45058 |
| Ga29 | 0.75000 | 0.95058 | 0.25000 |
| Ga30 | 0.75000 | 0.04941 | 0.75000 |
| Ga31 | 0.25000 | 0.04941 | 0.25000 |
| Ga32 | 0.25000 | 0.95058 | 0.75000 |
| Ga33 | 0.75000 | 0.25000 | 0.95058 |
| Ga34 | 0.25000 | 0.25000 | 0.04941 |
| Ga35 | 0.75000 | 0.75000 | 0.04941 |
| Ga36 | 0.25000 | 0.75000 | 0.95058 |
| Ga37 | 0.95058 | 0.75000 | 0.25000 |
| Ga38 | 0.04941 | 0.25000 | 0.25000 |
| Ga39 | 0.04941 | 0.75000 | 0.75000 |
| Ga40 | 0.95058 | 0.25000 | 0.75000 |
| Ga41 | 0.68352 | 0.00000 | 0.00000 |
| Ga42 | 0.31647 | 0.00000 | 0.00000 |
| Ga43 | 0.00000 | 0.68352 | 0.00000 |
| Ga44 | 0.00000 | 0.31647 | 0.00000 |
| Ga45 | 0.00000 | 0.00000 | 0.68352 |
| Ga46 | 0.00000 | 0.00000 | 0.31647 |
| Ga47 | 0.68352 | 0.50000 | 0.50000 |
| Ga48 | 0.31647 | 0.50000 | 0.50000 |
| Ga49 | 0.00000 | 0.18352 | 0.50000 |
| Ga50 | 0.00000 | 0.81647 | 0.50000 |
| Ga51 | 0.00000 | 0.50000 | 0.18352 |
| Ga52 | 0.00000 | 0.50000 | 0.81647 |
| Ga53 | 0.18352 | 0.00000 | 0.50000 |
| Ga54 | 0.81647 | 0.00000 | 0.50000 |
| Ga55 | 0.50000 | 0.68352 | 0.50000 |
| Ga56 | 0.50000 | 0.31647 | 0.50000 |
| Ga57 | 0.50000 | 0.00000 | 0.18352 |
| Ga58 | 0.50000 | 0.00000 | 0.81647 |

| | | | | |
|---|---|---|---|---|
| | Ga59 | 0.18352 | 0.50000 | 0.00000 |
| | Ga60 | 0.81647 | 0.50000 | 0.00000 |
| | Ga61 | 0.50000 | 0.18352 | 0.00000 |
| | Ga62 | 0.50000 | 0.81647 | 0.00000 |
| | Ga63 | 0.50000 | 0.50000 | 0.68352 |
| | Ga64 | 0.50000 | 0.50000 | 0.31647 |
| | Fe1 | 0.25000 | 0.75000 | 0.75000 |
| | Fe2 | 0.75000 | 0.25000 | 0.75000 |
| | Fe3 | 0.75000 | 0.75000 | 0.25000 |
| | Fe4 | 0.25000 | 0.25000 | 0.25000 |
| $Mo_4FeGa_{16.25}Ge$ | Mo1 | 0.65593 | 0.84710 | 0.84701 |
| | Mo2 | 0.34595 | 0.15522 | 0.84499 |
| | Mo3 | 0.34406 | 0.84710 | 0.15298 |
| | Mo4 | 0.65404 | 0.15522 | 0.15500 |
| | Mo5 | 0.84616 | 0.65469 | 0.84616 |
| | Mo6 | 0.84558 | 0.34475 | 0.15441 |
| | Mo7 | 0.15441 | 0.34475 | 0.84558 |
| | Mo8 | 0.15383 | 0.65469 | 0.15383 |
| | Mo9 | 0.84701 | 0.84710 | 0.65593 |
| | Mo10 | 0.15298 | 0.84710 | 0.34406 |
| | Mo11 | 0.84499 | 0.15522 | 0.34595 |
| | Mo12 | 0.15500 | 0.15522 | 0.65404 |
| | Mo13 | 0.65498 | 0.34087 | 0.34501 |
| | Mo14 | 0.35174 | 0.65313 | 0.35174 |
| | Mo15 | 0.34501 | 0.34087 | 0.65498 |
| | Mo16 | 0.64825 | 0.65313 | 0.64825 |
| | Ge1 | 0.00000 | 0.00015 | 0.00000 |
| | Ge2 | 0.00000 | 0.50128 | 0.50000 |
| | Ge3 | 0.50000 | 0.99565 | 0.50000 |
| | Ge4 | 0.50000 | 0.50128 | 0.00000 |
| | Ga1 | 0.88052 | 0.87720 | 0.88052 |
| | Ga2 | 0.12175 | 0.12109 | 0.87824 |
| | Ga3 | 0.11947 | 0.87720 | 0.11947 |
| | Ga4 | 0.87824 | 0.12109 | 0.12175 |
| | Ga5 | 0.87997 | 0.37874 | 0.37809 |
| | Ga6 | 0.12497 | 0.62061 | 0.37792 |
| | Ga7 | 0.12002 | 0.37874 | 0.62190 |
| | Ga8 | 0.87502 | 0.62061 | 0.62207 |
| | Ga9 | 0.37843 | 0.87600 | 0.37843 |
| | Ga10 | 0.62056 | 0.11879 | 0.37943 |
| | Ga11 | 0.62156 | 0.87600 | 0.62156 |
| | Ga12 | 0.37943 | 0.11879 | 0.62056 |
| | Ga13 | 0.37809 | 0.37874 | 0.87997 |
| | Ga14 | 0.62207 | 0.62061 | 0.87502 |
| | Ga15 | 0.62190 | 0.37874 | 0.12002 |
| | Ga16 | 0.37792 | 0.62061 | 0.12497 |
| | Ga17 | 0.45601 | 0.76647 | 0.77812 |
| | Ga18 | 0.54875 | 0.24775 | 0.75084 |
| | Ga19 | 0.54398 | 0.76647 | 0.22187 |
| | Ga20 | 0.45124 | 0.24775 | 0.24915 |

| | | | | |
|---|---|---|---|---|
| | Ga21 | 0.75572 | 0.45093 | 0.75572 |
| | Ga22 | 0.75017 | 0.54379 | 0.24982 |
| | Ga23 | 0.24982 | 0.54379 | 0.75017 |
| | Ga24 | 0.24427 | 0.45093 | 0.24427 |
| | Ga25 | 0.77812 | 0.76647 | 0.45601 |
| | Ga26 | 0.22187 | 0.76647 | 0.54398 |
| | Ga27 | 0.75084 | 0.24775 | 0.54875 |
| | Ga28 | 0.24915 | 0.24775 | 0.45124 |
| | Ga29 | 0.74634 | 0.94969 | 0.25365 |
| | Ga30 | 0.75154 | 0.05066 | 0.75154 |
| | Ga31 | 0.24845 | 0.05066 | 0.24845 |
| | Ga32 | 0.25365 | 0.94969 | 0.74634 |
| | Ga33 | 0.75031 | 0.25036 | 0.95028 |
| | Ga34 | 0.24968 | 0.25036 | 0.04971 |
| | Ga35 | 0.74697 | 0.74701 | 0.05379 |
| | Ga36 | 0.25302 | 0.74701 | 0.94620 |
| | Ga37 | 0.94620 | 0.74701 | 0.25302 |
| | Ga38 | 0.04971 | 0.25036 | 0.24968 |
| | Ga39 | 0.05379 | 0.74701 | 0.74697 |
| | Ga40 | 0.95028 | 0.25036 | 0.75031 |
| | Ga41 | 0.68556 | 0.00084 | 0.00215 |
| | Ga42 | 0.31443 | 0.00084 | 0.99784 |
| | Ga43 | 0.00000 | 0.68273 | 0.00000 |
| | Ga44 | 0.00000 | 0.31647 | 0.00000 |
| | Ga45 | 0.00215 | 0.00084 | 0.68556 |
| | Ga46 | 0.99784 | 0.00084 | 0.31443 |
| | Ga47 | 0.68765 | 0.48789 | 0.50684 |
| | Ga48 | 0.31234 | 0.48789 | 0.49315 |
| | Ga49 | 0.00000 | 0.18358 | 0.50000 |
| | Ga50 | 0.00000 | 0.82641 | 0.50000 |
| | Ga51 | 0.99913 | 0.49984 | 0.18205 |
| | Ga52 | 0.00086 | 0.49984 | 0.81794 |
| | Ga53 | 0.18372 | 0.00757 | 0.49401 |
| | Ga54 | 0.81627 | 0.00757 | 0.50598 |
| | Ga55 | 0.50000 | 0.31247 | 0.50000 |
| | Ga56 | 0.49401 | 0.00757 | 0.18372 |
| | Ga57 | 0.50598 | 0.00757 | 0.81627 |
| | Ga58 | 0.18205 | 0.49984 | 0.99913 |
| | Ga59 | 0.81794 | 0.49984 | 0.00086 |
| | Ga60 | 0.50000 | 0.18358 | 0.00000 |
| | Ga61 | 0.50000 | 0.82641 | 0.00000 |
| | Ga62 | 0.50684 | 0.48789 | 0.68765 |
| | Ga63 | 0.49315 | 0.48789 | 0.31234 |
| | Ga64 | 0.42164 | 0.68234 | 0.57835 |
| | Ga65 | 0.57835 | 0.68234 | 0.42164 |
| | Fe1 | 0.26018 | 0.74555 | 0.73981 |
| | Fe2 | 0.74899 | 0.25107 | 0.74899 |
| | Fe3 | 0.73981 | 0.74555 | 0.26018 |
| | Fe4 | 0.25100 | 0.25107 | 0.25100 |
| Ga | Ga1 | 0.84452 | 0.15547 | 0.08188 |

| | | | | |
|---|---|---|---|---|
| | Ga2 | 0.65547 | 0.34452 | 0.58188 |
| | Ga3 | 0.34452 | 0.65547 | 0.41811 |
| | Ga4 | 0.15547 | 0.84452 | 0.91811 |

**Table S14. Parameters for VASP raMO Calculations.**[a]

| Structure | Energy cutoff (eV) | k-point grid | FFT grid | Total Energy/cell (eV) |
|---|---|---|---|---|
| $Mo_4FeGa_{16}Ge$ | 334.9 | 3×3×3 | 120×120×120 | -429.4280 |

[a]All calculations were performed using the PAW-PW91 atomic potentials. The k-point grid is Γ centered.

**Table S15. Lattice Parameters for VASP Calculations used in raMO Analysis.**

| Structure | *a* (Å) | *b* (Å) | *c* (Å) | α (°) | β (°) | γ (°) |
|---|---|---|---|---|---|---|
| $Mo_4FeGa_{16}Ge$ | 11.5800 | 11.5800 | 11.5800 | 90.000 | 90.000 | 90.000 |

**Table S16. Fractional Coordinates for VASP raMO Calculations.**

| Structure | Site | *x* | *y* | *z* |
|---|---|---|---|---|
| $Mo_4FeGa_{16}Ge$ | Fe1 | 0.75000 | 0.75000 | 0.75000 |
| | Fe2 | 0.25000 | 0.25000 | 0.75000 |
| | Fe3 | 0.25000 | 0.75000 | 0.25000 |
| | Fe4 | 0.75000 | 0.25000 | 0.25000 |
| | Ga1 | 0.87898 | 0.12101 | 0.87898 |
| | Ga2 | 0.37898 | 0.62101 | 0.87898 |
| | Ga3 | 0.37898 | 0.12101 | 0.37898 |
| | Ga4 | 0.37898 | 0.87898 | 0.62101 |
| | Ga5 | 0.37898 | 0.37898 | 0.12101 |
| | Ga6 | 0.87898 | 0.87898 | 0.12101 |
| | Ga7 | 0.87898 | 0.37898 | 0.62101 |
| | Ga8 | 0.12101 | 0.37898 | 0.37898 |
| | Ga9 | 0.12101 | 0.87898 | 0.87898 |
| | Ga10 | 0.62101 | 0.37898 | 0.87898 |
| | Ga11 | 0.62101 | 0.87898 | 0.37898 |
| | Ga12 | 0.62101 | 0.12101 | 0.62101 |
| | Ga13 | 0.62101 | 0.62101 | 0.12101 |
| | Ga14 | 0.12101 | 0.12101 | 0.12101 |
| | Ga15 | 0.87898 | 0.62101 | 0.37898 |
| | Ga16 | 0.12101 | 0.62101 | 0.62101 |
| | Ga17 | 0.75000 | 0.45048 | 0.25000 |
| | Ga18 | 0.25000 | 0.45048 | 0.75000 |
| | Ga19 | 0.25000 | 0.95048 | 0.25000 |
| | Ga20 | 0.75000 | 0.54951 | 0.75000 |
| | Ga21 | 0.75000 | 0.04951 | 0.25000 |
| | Ga22 | 0.25000 | 0.54951 | 0.25000 |
| | Ga23 | 0.25000 | 0.04951 | 0.75000 |
| | Ga24 | 0.54951 | 0.25000 | 0.25000 |
| | Ga25 | 0.54951 | 0.75000 | 0.75000 |
| | Ga26 | 0.04951 | 0.25000 | 0.75000 |
| | Ga27 | 0.04951 | 0.75000 | 0.25000 |
| | Ga28 | 0.45048 | 0.25000 | 0.75000 |
| | Ga29 | 0.45048 | 0.75000 | 0.25000 |

| | | | |
|---|---|---|---|
| Ga30 | 0.95048 | 0.25000 | 0.25000 |
| Ga31 | 0.95048 | 0.75000 | 0.75000 |
| Ga32 | 0.75000 | 0.95048 | 0.75000 |
| Ga33 | 0.25000 | 0.75000 | 0.45048 |
| Ga34 | 0.25000 | 0.25000 | 0.95048 |
| Ga35 | 0.75000 | 0.75000 | 0.95048 |
| Ga36 | 0.75000 | 0.25000 | 0.45048 |
| Ga37 | 0.75000 | 0.75000 | 0.54951 |
| Ga38 | 0.75000 | 0.25000 | 0.04951 |
| Ga39 | 0.25000 | 0.75000 | 0.04951 |
| Ga40 | 0.25000 | 0.25000 | 0.54951 |
| Ga41 | 0.00000 | 0.68351 | 0.00000 |
| Ga42 | 0.18351 | 0.50000 | 0.00000 |
| Ga43 | 0.68351 | 0.00000 | 0.00000 |
| Ga44 | 0.68351 | 0.50000 | 0.50000 |
| Ga45 | 0.00000 | 0.00000 | 0.68351 |
| Ga46 | 0.00000 | 0.50000 | 0.18351 |
| Ga47 | 0.50000 | 0.00000 | 0.18351 |
| Ga48 | 0.50000 | 0.50000 | 0.68351 |
| Ga49 | 0.00000 | 0.00000 | 0.31648 |
| Ga50 | 0.00000 | 0.50000 | 0.81648 |
| Ga51 | 0.50000 | 0.00000 | 0.81648 |
| Ga52 | 0.50000 | 0.50000 | 0.31648 |
| Ga53 | 0.00000 | 0.81648 | 0.50000 |
| Ga54 | 0.00000 | 0.31648 | 0.00000 |
| Ga55 | 0.50000 | 0.81648 | 0.00000 |
| Ga56 | 0.50000 | 0.31648 | 0.50000 |
| Ga57 | 0.31648 | 0.50000 | 0.50000 |
| Ga58 | 0.31648 | 0.00000 | 0.00000 |
| Ga59 | 0.81648 | 0.50000 | 0.00000 |
| Ga60 | 0.81648 | 0.00000 | 0.50000 |
| Ga61 | 0.50000 | 0.68351 | 0.50000 |
| Ga62 | 0.50000 | 0.18351 | 0.00000 |
| Ga63 | 0.00000 | 0.18351 | 0.50000 |
| Ga64 | 0.18351 | 0.00000 | 0.50000 |
| Ge1 | 0.00000 | 0.00000 | 0.00000 |
| Ge2 | 0.50000 | 0.50000 | 0.00000 |
| Ge3 | 0.00000 | 0.50000 | 0.50000 |
| Ge4 | 0.50000 | 0.00000 | 0.50000 |
| Mo1 | 0.65509 | 0.65509 | 0.34490 |
| Mo2 | 0.65509 | 0.15509 | 0.84490 |
| Mo3 | 0.15509 | 0.65509 | 0.84490 |
| Mo4 | 0.15509 | 0.15509 | 0.34490 |
| Mo5 | 0.65509 | 0.34490 | 0.65509 |
| Mo6 | 0.65509 | 0.84490 | 0.15509 |
| Mo7 | 0.15509 | 0.34490 | 0.15509 |
| Mo8 | 0.15509 | 0.84490 | 0.65509 |
| Mo9 | 0.34490 | 0.34490 | 0.34490 |
| Mo10 | 0.34490 | 0.84490 | 0.84490 |
| Mo11 | 0.84490 | 0.34490 | 0.84490 |
| Mo12 | 0.84490 | 0.84490 | 0.34490 |

| | | | | |
|---|---|---|---|---|
| | Mo13 | 0.84490 | 0.15509 | 0.65509 |
| | Mo14 | 0.84490 | 0.65509 | 0.15509 |
| | Mo15 | 0.34490 | 0.15509 | 0.15509 |
| | Mo16 | 0.34490 | 0.65509 | 0.65509 |

**Table S17. Parameters for ABINIT Phonon Calculations.**[a]

| Structure | Energy cutoff (Ha) | q-point grid | FFT grid | Total Energy/cell (Ha) |
|---|---|---|---|---|
| $Mo_4FeGa_{16}Ge$ | 18.3747 | 3×3×3 | 60×60×60 | -47912.7385 |

[a]All calculations were performed using the PAW-PBE atomic potentials. The k-point and q-point grids are Γ centered.

**Table S18. Lattice Parameters for ABINIT Phonon Calculations.**

| Structure | *a* (Å) | *b* (Å) | *c* (Å) | α (°) | β (°) | γ (°) |
|---|---|---|---|---|---|---|
| $Mo_4FeGa_{16}Ge$ | 8.2046 | 8.2046 | 8.2046 | 60.000 | 60.000 | 60.000 |

**Table S19. Fractional Coordinates for ABINIT Phonon Calculations.**

| Structure | Multiplicity | Site | *x* | *y* | *z* |
|---|---|---|---|---|---|
| $Mo_4FeGa_{16}Ge$ | 16 | Mo1 | 0.03305 | 0.65565 | 0.65565 |
| | 4 | Fe1 | 0.25000 | 0.25000 | 0.25000 |
| | 24 | Ga1 | 0.04884 | 0.04884 | 0.45116 |
| | 24 | Ga2 | 0.31656 | 0.31656 | 0.68344 |
| | 16 | Ga3 | 0.87963 | 0.87963 | 0.36112 |
| | 4 | Ge1 | 0.00000 | 0.00000 | 0.00000 |

**Table S20. Parameters for ABINIT Calculations for DFT-Chemical Pressure Analysis.**[a]

| Structure | Energy cutoff (Ha) | k-point grid | FFT grid | Total Energy/cell (eV) |
|---|---|---|---|---|
| $Mo_4FeGa_{16}Ge$ | 70 | 3×3×3 | 120×120×120 | -429.4280 |

[a]Performed using the valence-only LDA HGH norm-conserving pseudopotentials. The k-point and q-point grids are Γ centered.

**Table S21. Optimized Lattice Parameters for Structure Used in DFT-Chemical Pressure Analysis.**

| Structure | *a* (Å) | *b* (Å) | *c* (Å) | α (°) | β (°) | γ (°) |
|---|---|---|---|---|---|---|
| $Mo_4FeGa_{16}Ge$ | 7.9180 | 7.9180 | 7.9180 | 60.000 | 60.000 | 60.000 |

**Table S22. Fractional Coordinates for Structure Used in ABINIT Phonon Calculations.**

| Structure | Multiplicity | Site | *x* | *y* | *z* |
|---|---|---|---|---|---|
| $Mo_4FeGa_{16}Ge$ | 16 | Mo1 | 0.65453 | 0.03642 | 0.65453 |
| | 4 | Fe1 | 0.25000 | 0.25000 | 0.25000 |
| | 24 | Ga1 | 0.04944 | 0.45056 | 0.04944 |
| | 24 | Ga2 | 0.31679 | 0.68321 | 0.31679 |
| | 16 | Ga3 | 0.87967 | 0.36098 | 0.87967 |
| | 4 | Ge1 | 0.00000 | 0.00000 | 0.00000 |

## S5. Electronic DOS Distributions

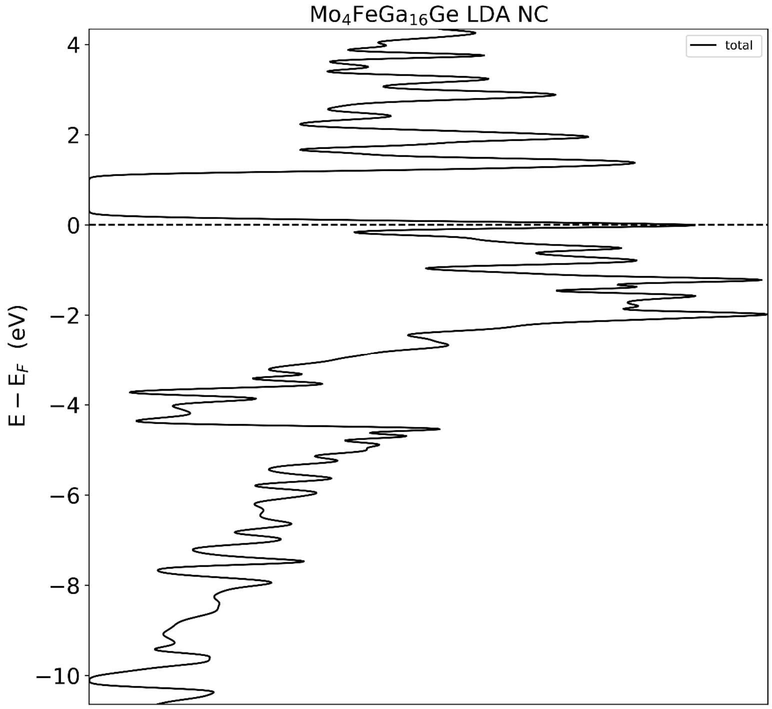


**Figure S2.** DOS for dumbbell-free model generated using the ABINIT package with norm conserving HGH pseudopotentials. A Gaussian broadening with standard deviation of 0.05 eV has been applied.

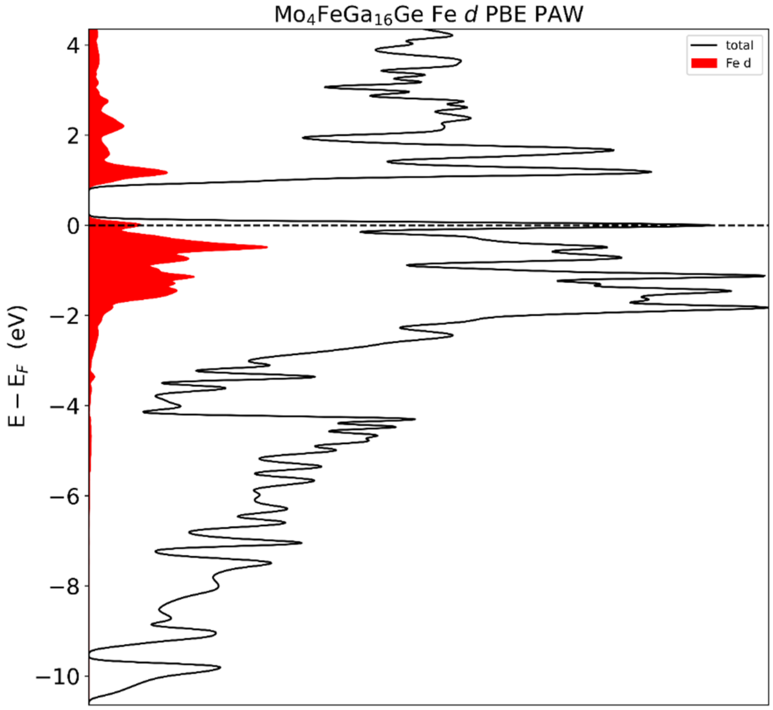


**Figure S3.** Fe *d* projected DOS for dumbbell-free model generated using the ABINIT package with the PAW approach and PBE functional. Gaussian broadening with standard deviation of 0.05 eV has been applied.

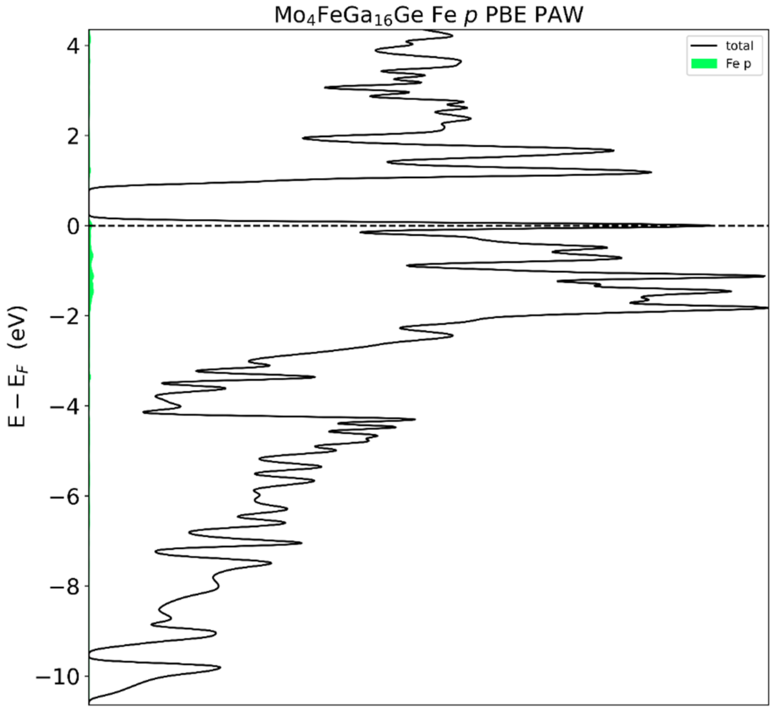


**Figure S4.** Fe *p* projected DOS for dumbbell-free model generated using the ABINIT package with the PAW approach and PBE functional. Gaussian broadening with standard deviation of 0.05 eV has been applied.

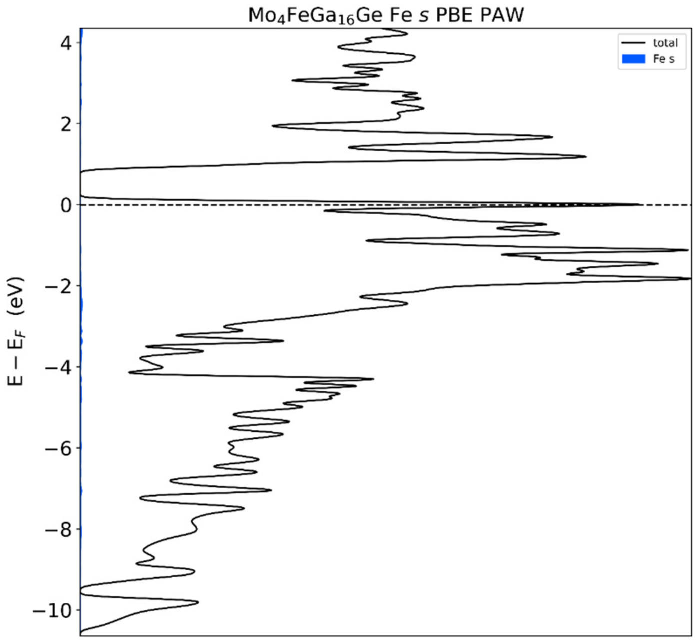


**Figure S5.** Fe *s* projected DOS for dumbbell-free model generated using the ABINIT package with the PAW approach and PBE functional. Gaussian broadening with standard deviation of 0.05 eV has been applied.

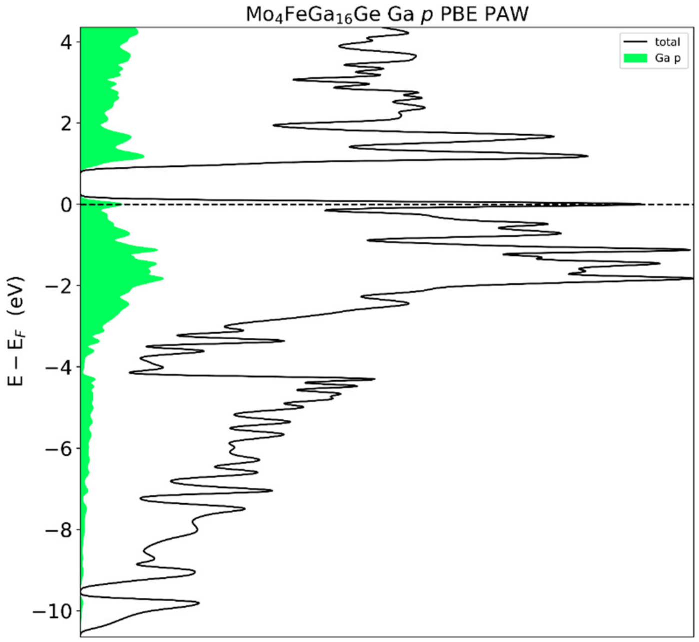


**Figure S6.** Ga *p* projected DOS for dumbbell-free model generated using the ABINIT package with the PAW approach and PBE functional. Gaussian broadening with standard deviation of 0.05 eV has been applied.

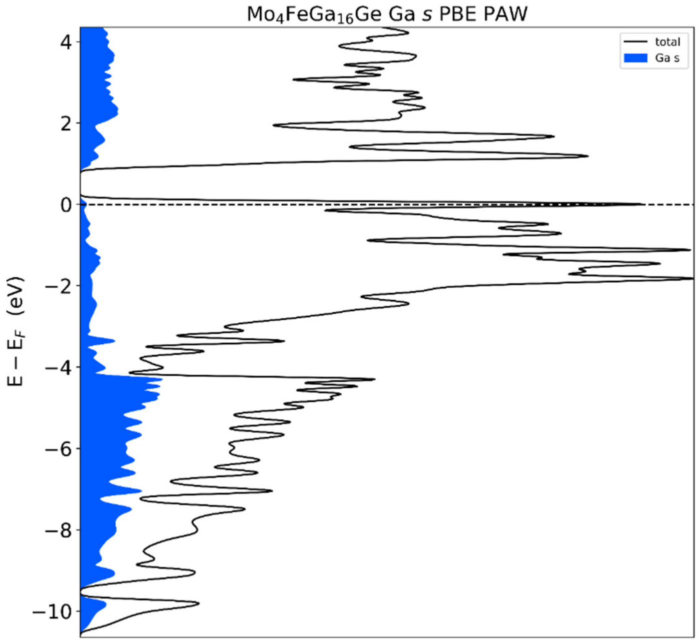


**Figure S7.** Ga *s* projected DOS for dumbbell-free model generated using the ABINIT package with the PAW approach and PBE functional. Gaussian broadening with standard deviation of 0.05 eV has been applied.

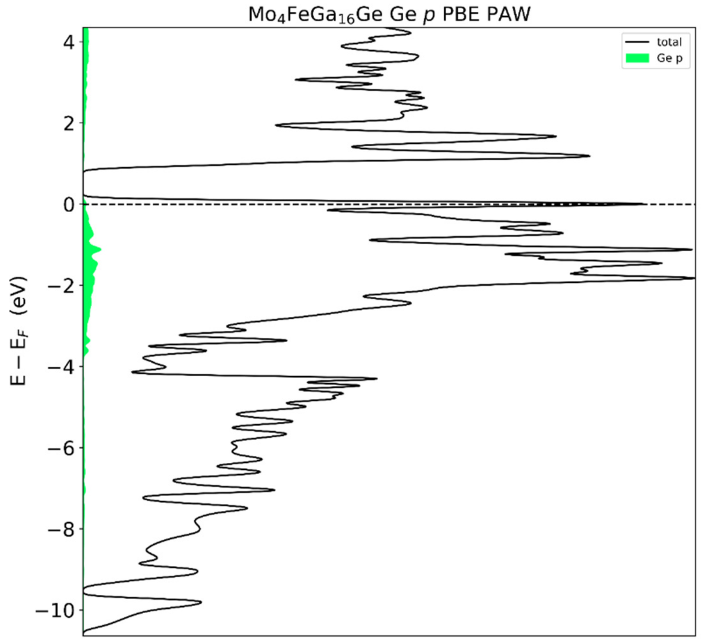


**Figure S8.** Ge *p* projected DOS for dumbbell-free model generated using the ABINIT package with the PAW approach and PBE functional. Gaussian broadening with standard deviation of 0.05 eV has been applied.

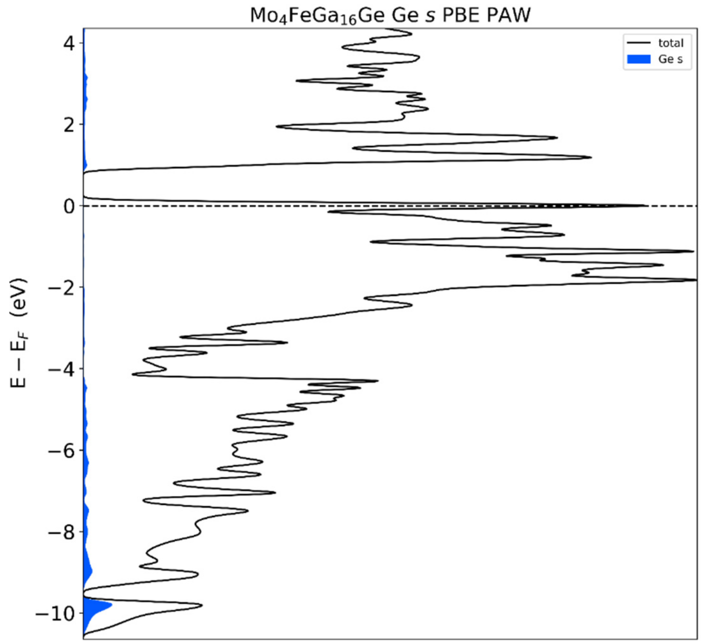


**Figure S9.** Ge *s* projected DOS for dumbbell-free model generated using the ABINIT package with the PAW approach and PBE functional. Gaussian broadening with standard deviation of 0.05 eV has been applied.

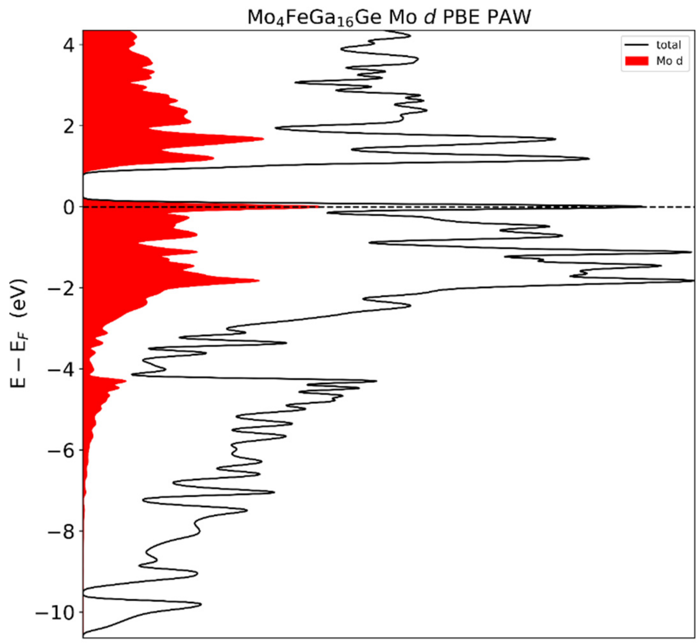


**Figure S10.** Mo *d* projected DOS for dumbbell-free model generated using the ABINIT package with the PAW approach and PBE functional. Gaussian broadening with standard deviation of 0.05 eV has been applied.

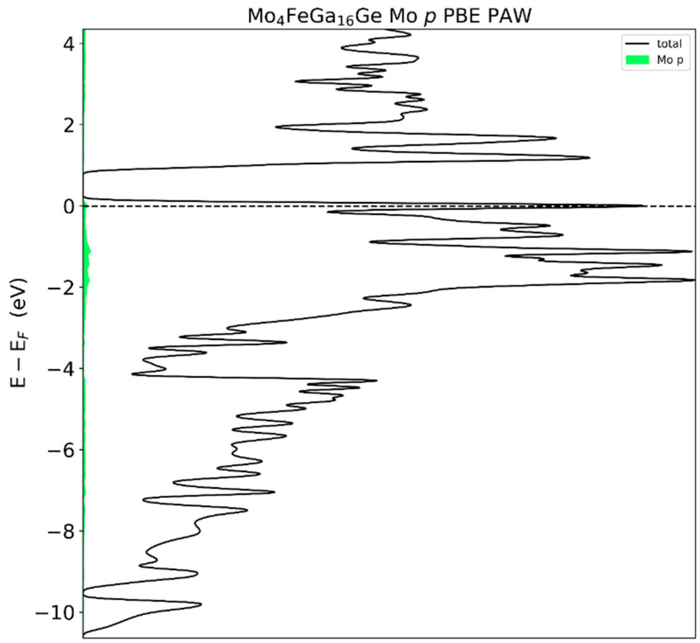


**Figure S11.** Mo *p* projected DOS for dumbbell-free model generated using the ABINIT package with the PAW approach and PBE functional. Gaussian broadening with standard deviation of 0.05 eV has been applied.

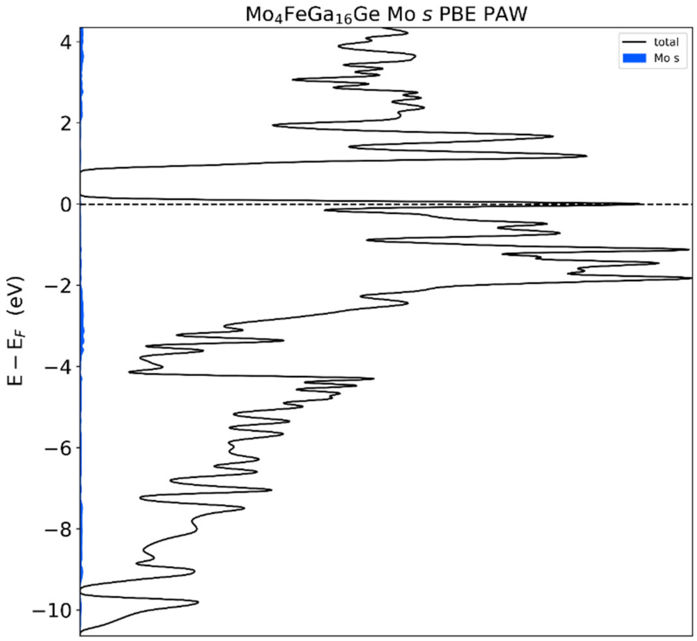


**Figure S12.** Mo *s* projected DOS for dumbbell-free model generated using the ABINIT package with the PAW approach and PBE functional. Gaussian broadening with standard deviation of 0.05 eV has been applied.

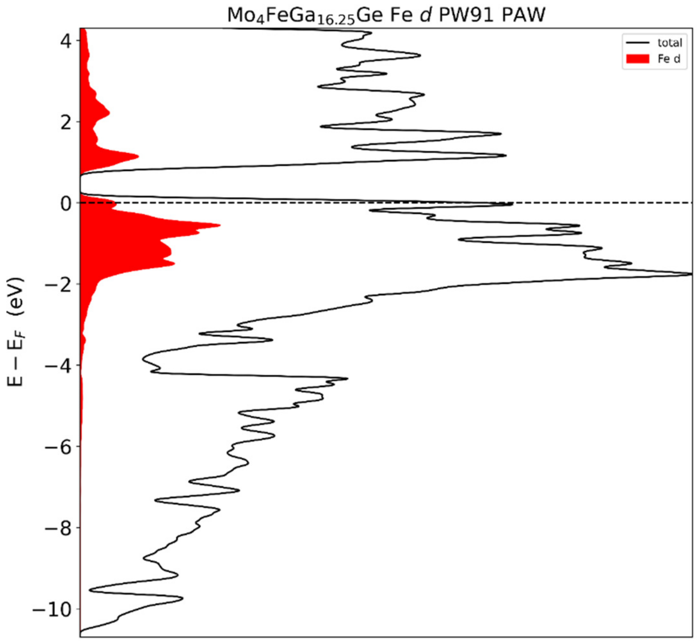


**Figure S13.** Fe *d* projected DOS for dumbbell-containing model generated using the VASP package with the PAW approach and PW91 functional. Gaussian broadening with standard deviation of 0.05 eV has been applied.

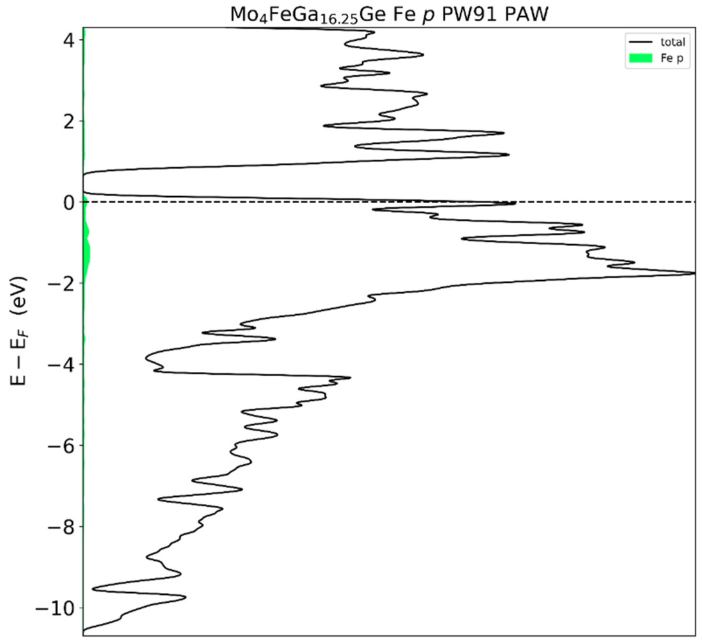


**Figure S14.** Fe *p* projected DOS for dumbbell-containing model generated using the VASP package with the PAW approach and PW91 functional. Gaussian broadening with standard deviation of 0.05 eV has been applied.

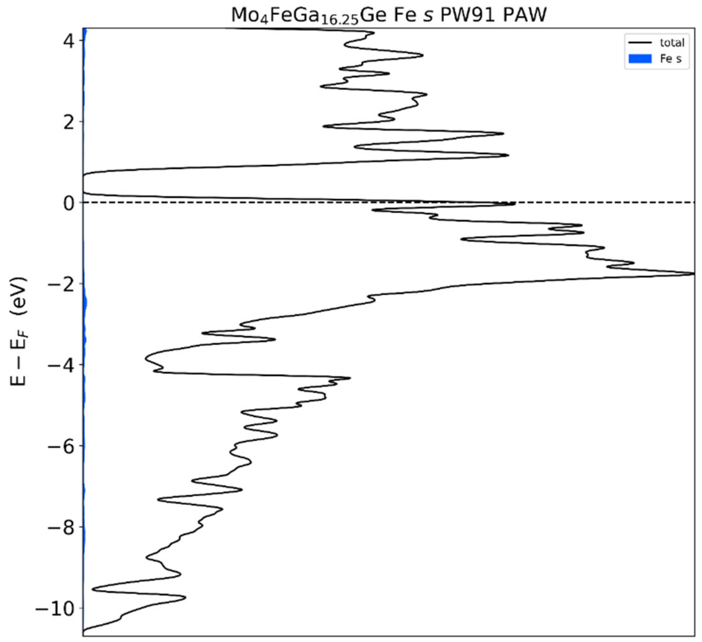


**Figure S15.** Fe *s* projected DOS for dumbbell-containing model generated using the VASP package with the PAW approach and PW91 functional. Gaussian broadening with standard deviation of 0.05 eV has been applied.

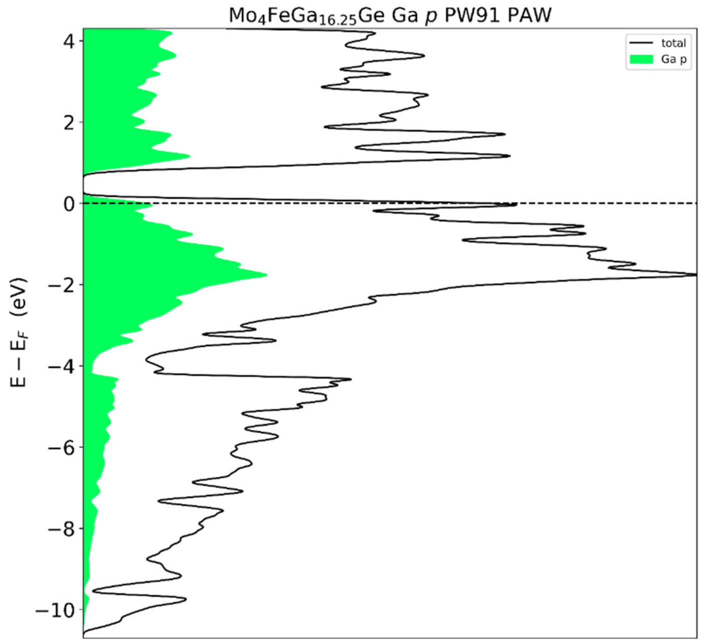


**Figure S16.** Ga *p* projected DOS for dumbbell-containing model generated using the VASP package with the PAW approach and PW91 functional. Gaussian broadening with standard deviation of 0.05 eV has been applied.

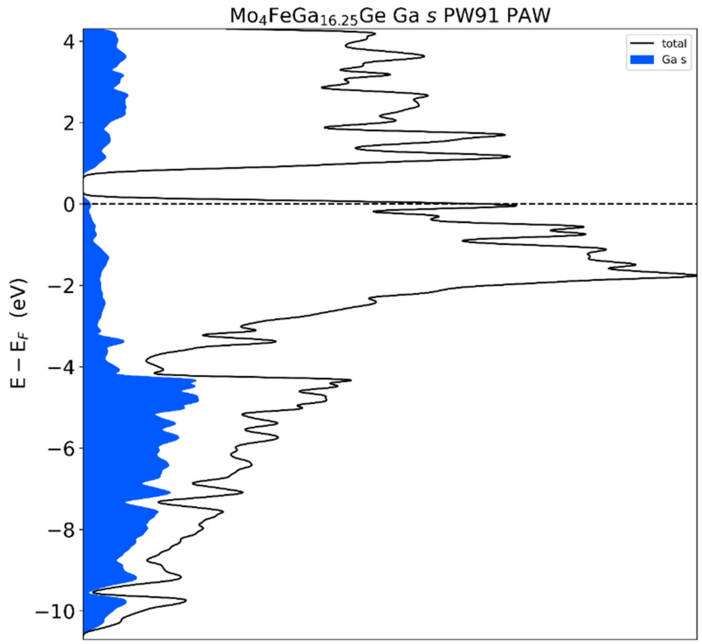


**Figure S17.** Ga *s* projected DOS for dumbbell-containing model generated using the VASP package with the PAW approach and PW91 functional. Gaussian broadening with standard deviation of 0.05 eV has been applied.

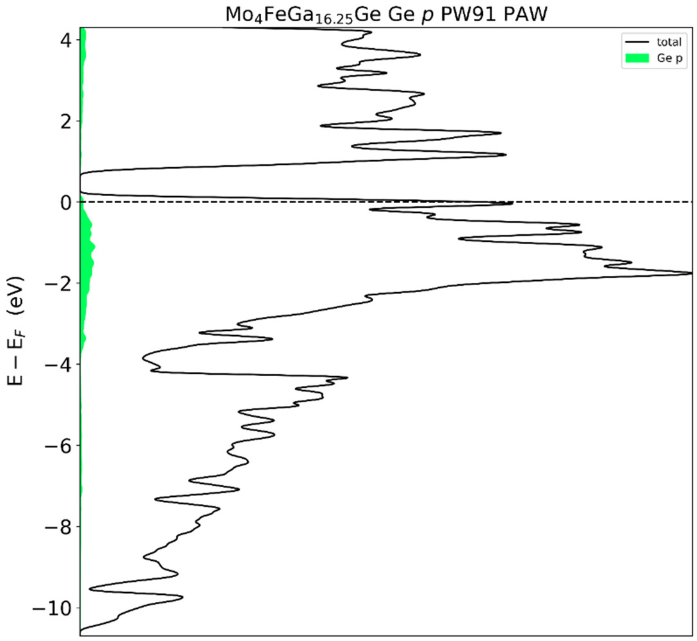


**Figure S18.** Ge *p* projected DOS for dumbbell-containing model generated using the VASP package with the PAW approach and PW91 functional. Gaussian broadening with standard deviation of 0.05 eV has been applied.

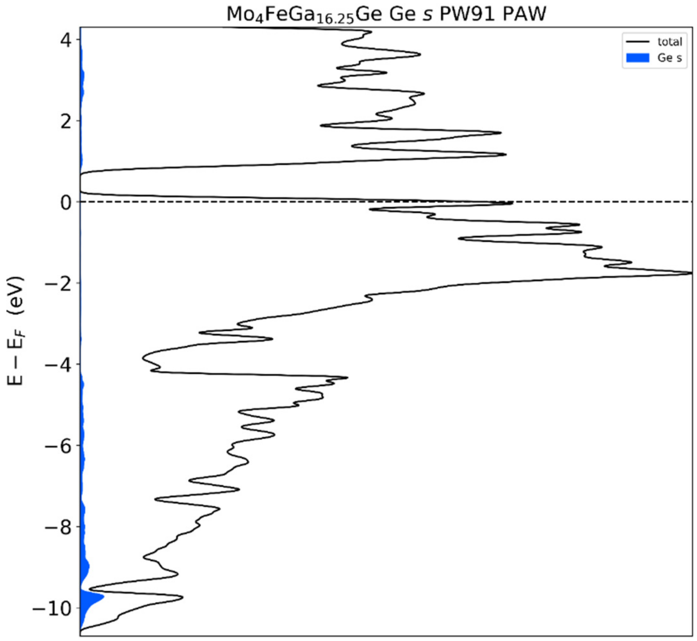


**Figure S19.** Ge *s* projected DOS for dumbbell-containing model generated using the VASP package with the PAW approach and PW91 functional. Gaussian broadening with standard deviation of 0.05 eV has been applied.

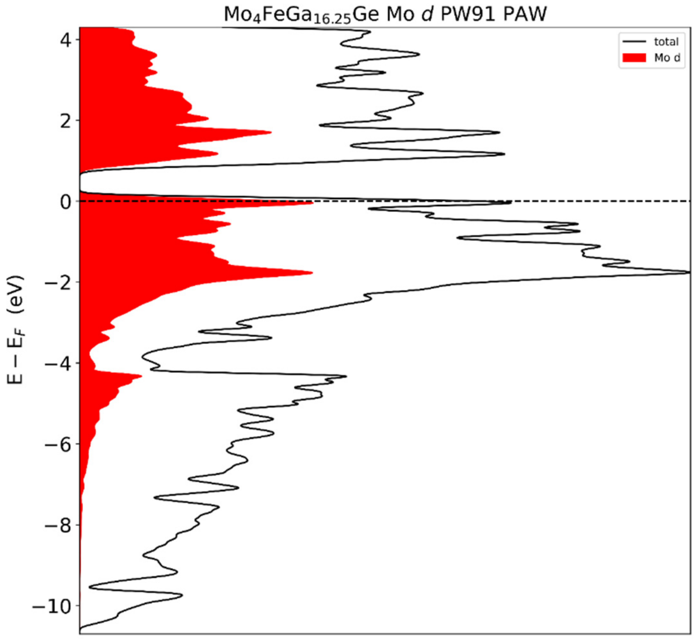


**Figure S20.** Mo *d* projected DOS for dumbbell-containing model generated using the VASP package with the PAW approach and PW91 functional. Gaussian broadening with standard deviation of 0.05 eV has been applied.

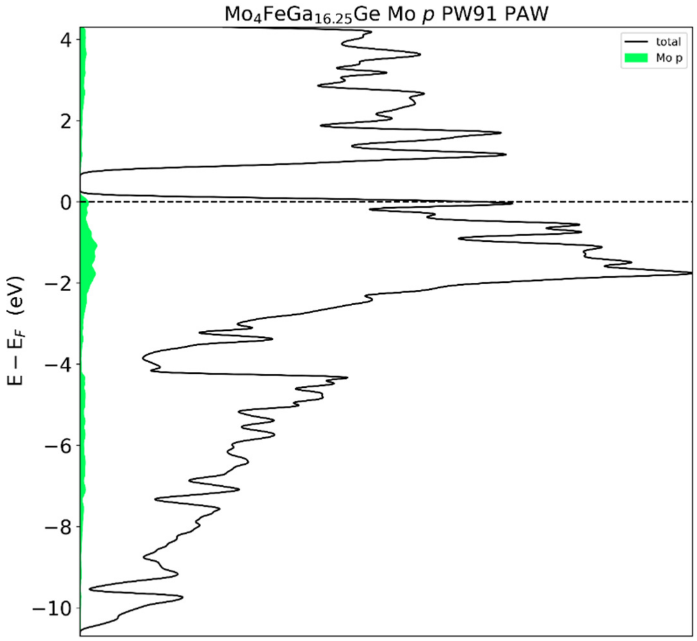


**Figure S21.** Mo *p* projected DOS for dumbbell-containing model generated using the VASP package with the PAW approach and PW91 functional. Gaussian broadening with standard deviation of 0.05 eV has been applied.

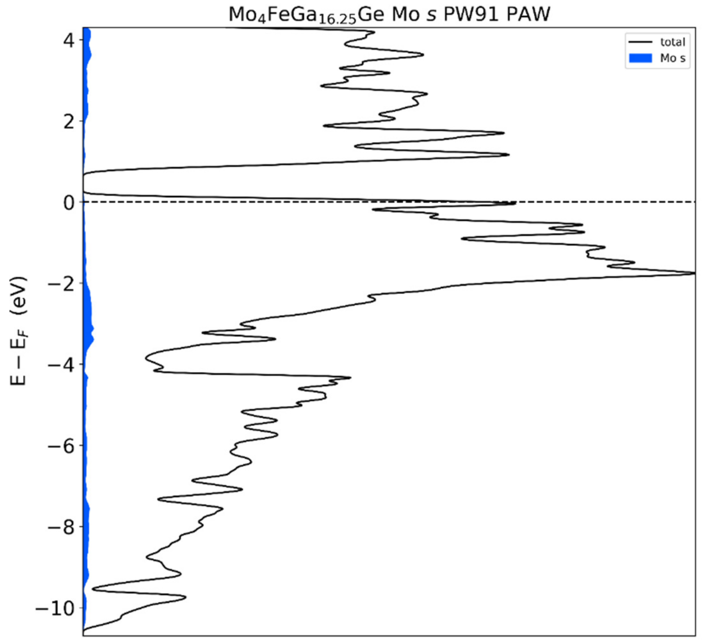


**Figure S22.** Mo *s* projected DOS for dumbbell-containing model generated using the VASP package with the PAW approach and PW91 functional. Gaussian broadening with standard deviation of 0.05 eV has been applied.

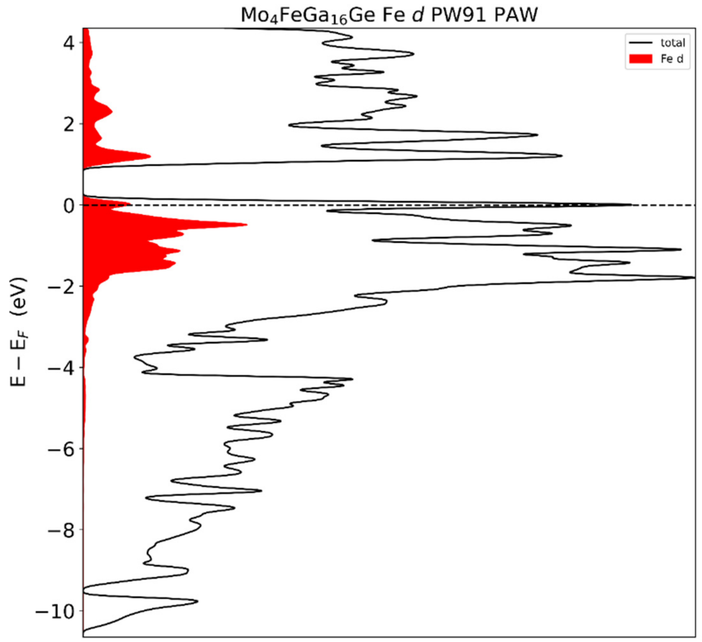


**Figure S23.** Fe *d* projected DOS for dumbbell-free model generated using the VASP package with the PAW approach and PW91 functional. Gaussian broadening with standard deviation of 0.05 eV has been applied.

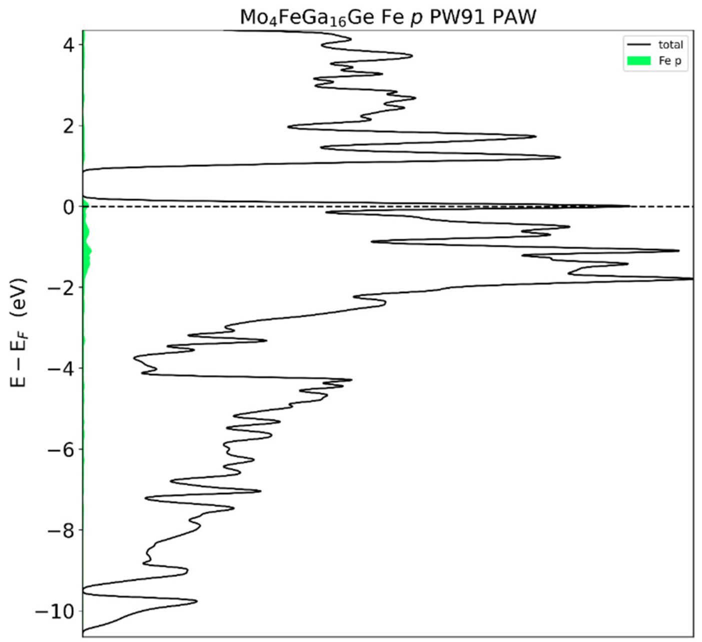


**Figure S24.** Fe *p* projected DOS for dumbbell-free model generated using the VASP package with the PAW approach and PW91 functional. Gaussian broadening with standard deviation of 0.05 eV has been applied.

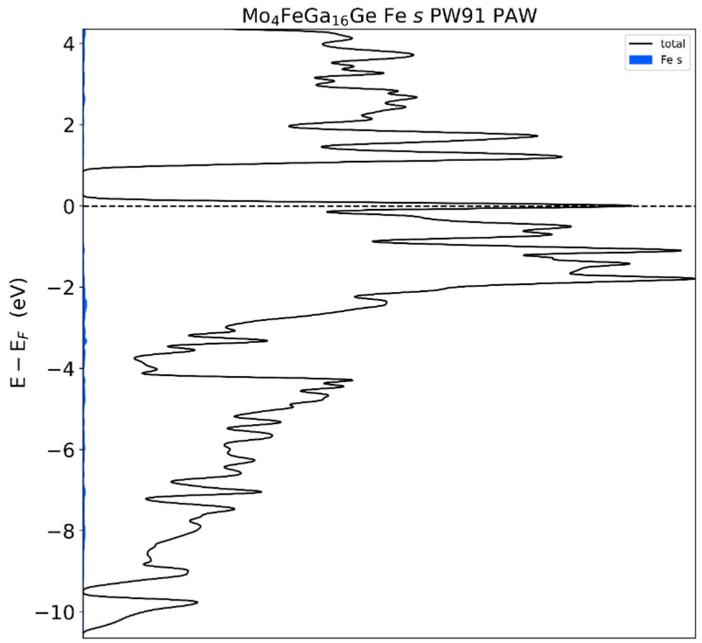


**Figure S25.** Fe *s* projected DOS for dumbbell-free model generated using the VASP package with the PAW approach and PW91 functional. Gaussian broadening with standard deviation of 0.05 eV has been applied.

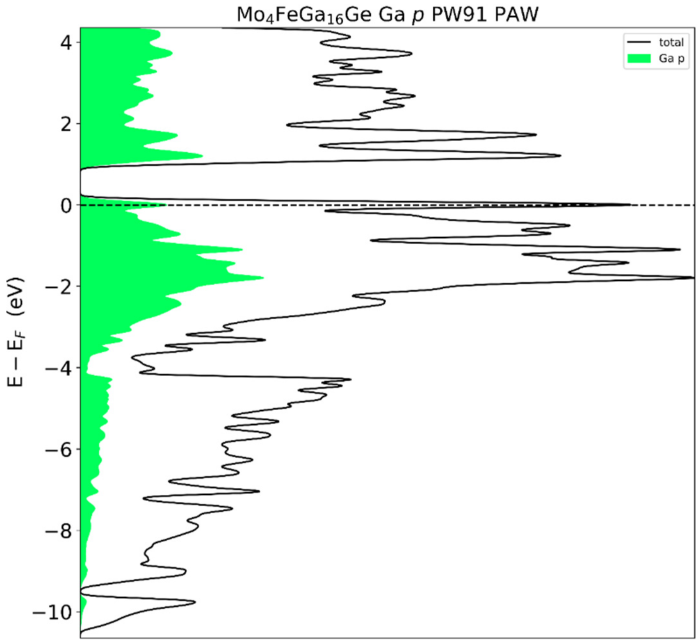


**Figure S26.** Ga *p* projected DOS for dumbbell-free model generated using the VASP package with the PAW approach and PW91 functional. Gaussian broadening with standard deviation of 0.05 eV has been applied.

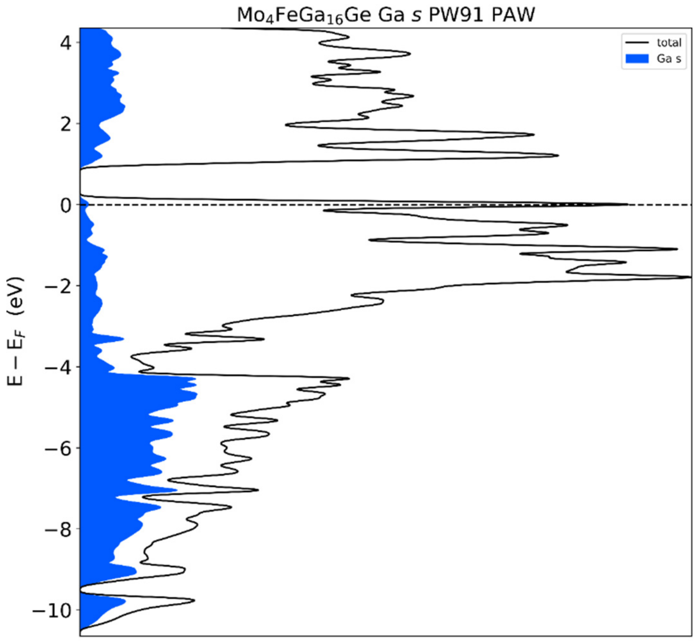


**Figure S27.** Ga *s* projected DOS for dumbbell-free model generated using the VASP package with the PAW approach and PW91 functional. Gaussian broadening with standard deviation of 0.05 eV has been applied.

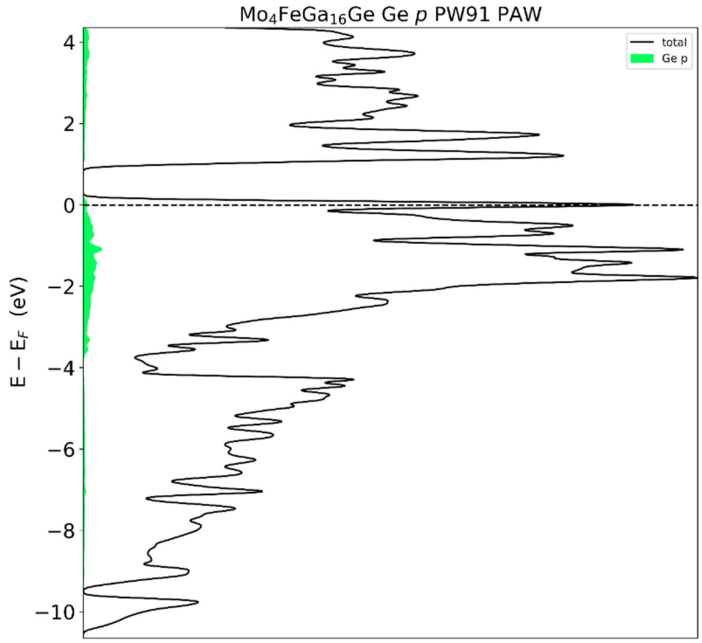


**Figure S28.** Ge *p* projected DOS for dumbbell-free model generated using the VASP package with the PAW approach and PW91 functional. Gaussian broadening with standard deviation of 0.05 eV has been applied.

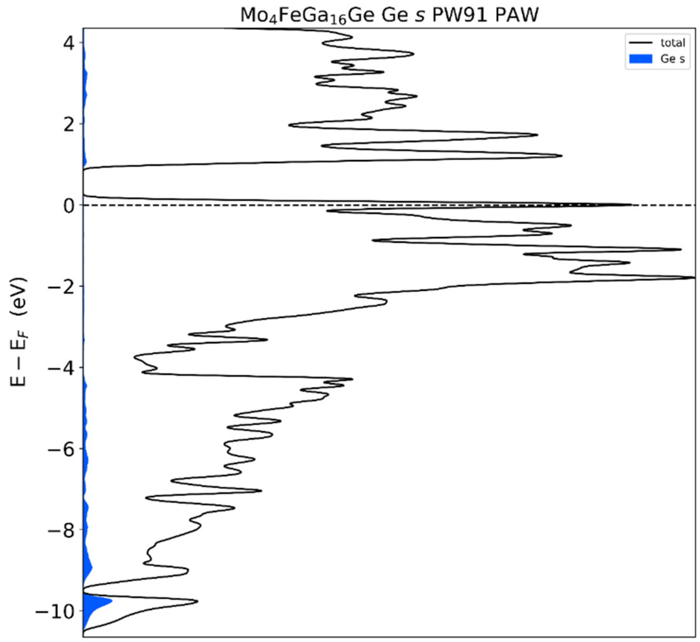


**Figure S29.** Ge *s* projected DOS for dumbbell-free model generated using the VASP package with the PAW approach and PW91 functional. Gaussian broadening with standard deviation of 0.05 eV has been applied.

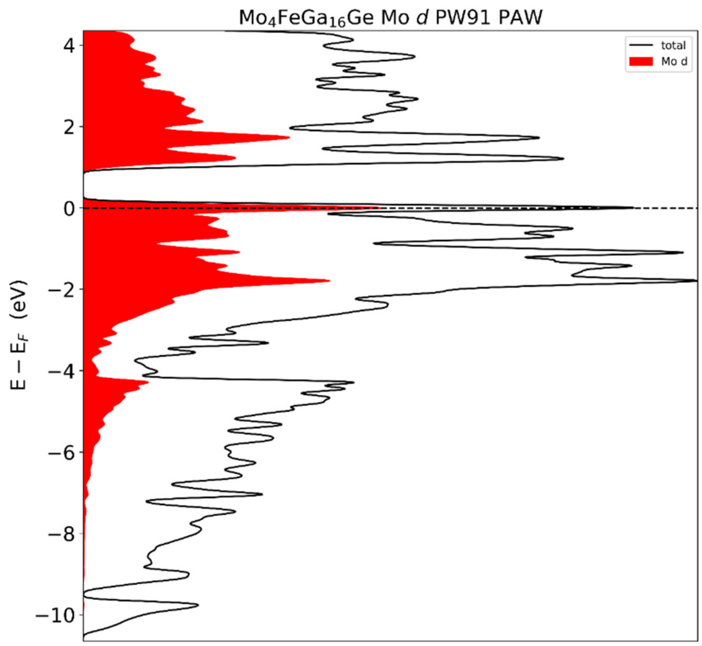


**Figure S30.** Mo *d* projected DOS for dumbbell-free model generated using the VASP package with the PAW approach and PW91 functional. Gaussian broadening with standard deviation of 0.05 eV has been applied.

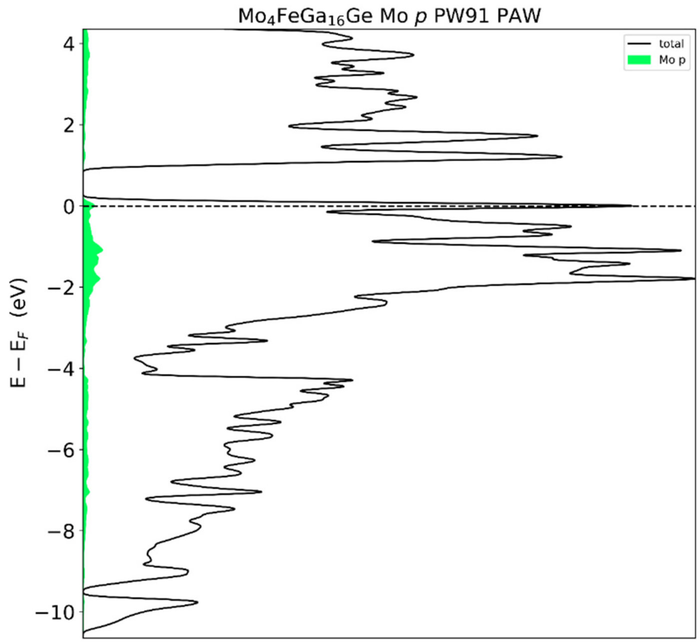


**Figure S31.** Mo *p* projected DOS for dumbbell-free model generated using the VASP package with the PAW approach and PW91 functional. Gaussian broadening with standard deviation of 0.05 eV has been applied.

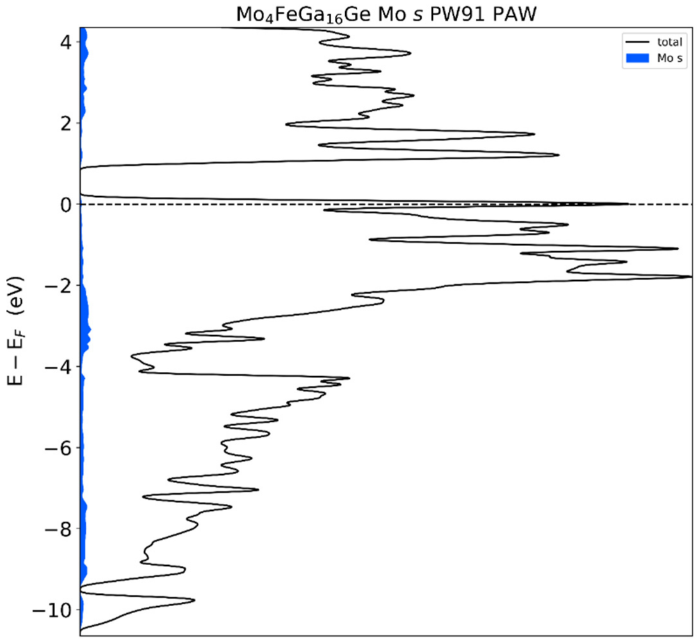


**Figure S32.** Mo *s* projected DOS for dumbbell-free model generated using the VASP package with the PAW approach and PW91 functional. Gaussian broadening with standard deviation of 0.05 eV has been applied.

# S6. Phonon Band Structure for Dumbbell-Free $Mo_4FeGa_{16}Ge$ Model

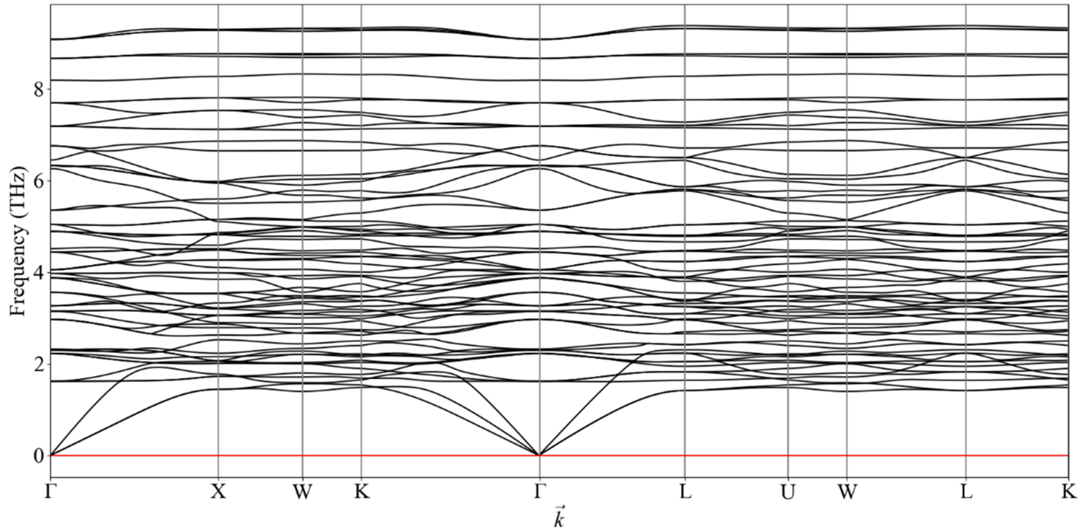


**Figure S33.** Phonon band structure calculated for a dumbbell-free model of $Mo_4Ga_{17.25-x}Ge_x$ with composition $Mo_4FeGa_{16}Ge$.

## S7. Phonon Modes at the Γ point for Dumbbell-Free $Mo_4FeGa_{16}Ge$ Model.

**Figure S34.** Phonon modes at the Γ-point calculated for a dumbbell free model of $Mo_4FeGa_{17.25-x}Ga_x$ with composition of $Mo_4FeGa_{16}Ge$ (below and continued over more than 30 pages).

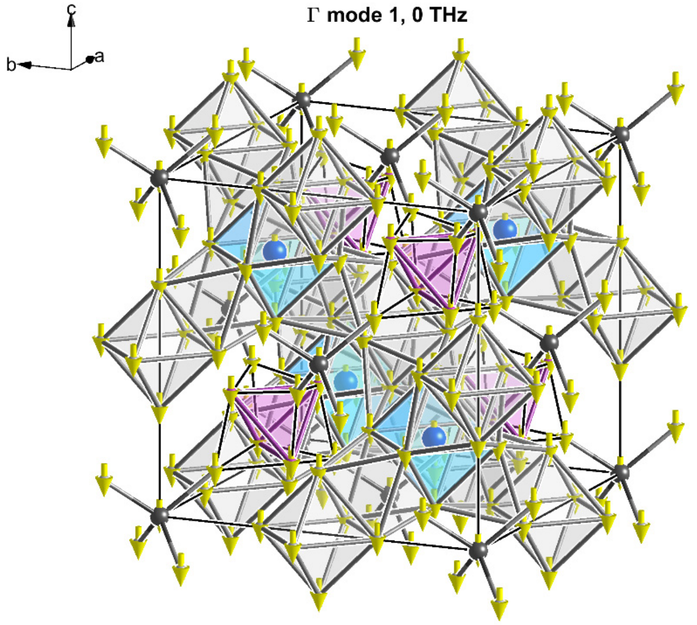


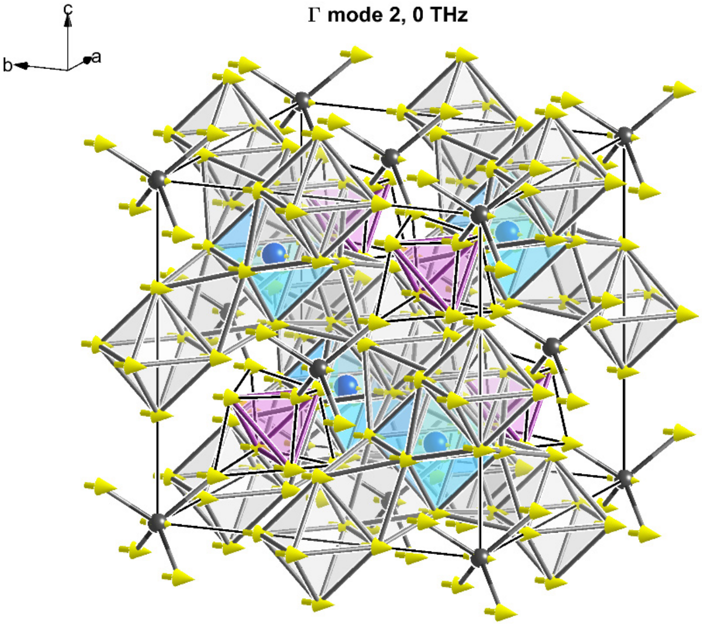

Γ mode 3, 7.2283e-05 THz

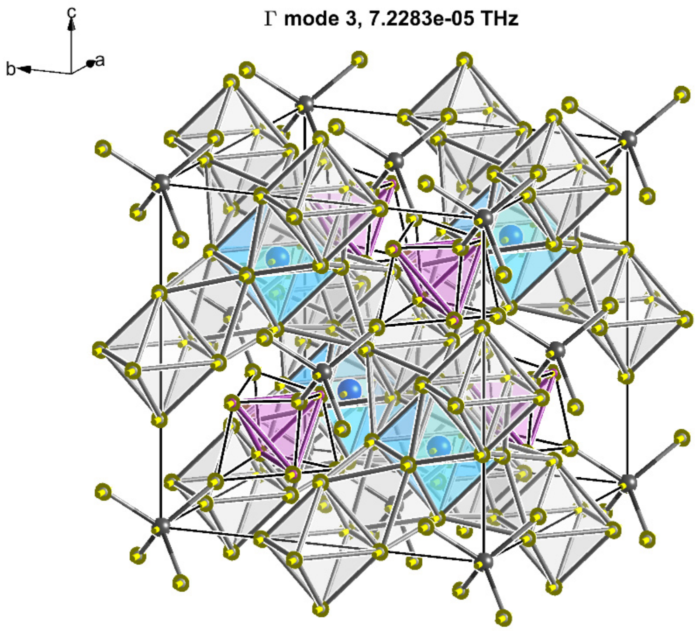


Γ mode 4, 1.6158 THz

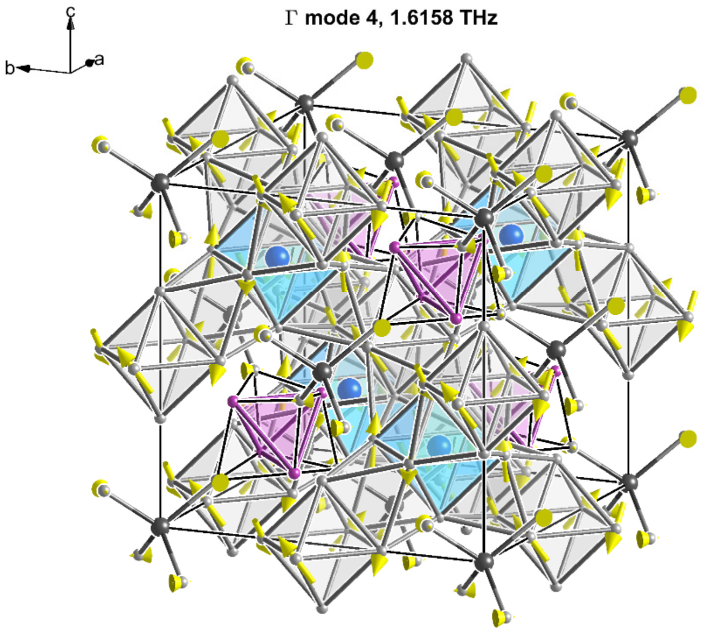

Γ mode 5, 1.6158 THz

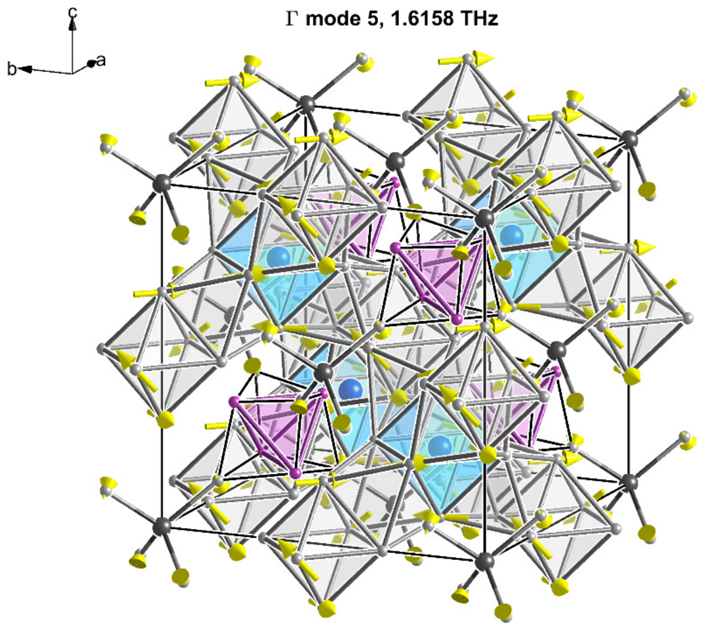


Γ mode 6, 1.6158 THz

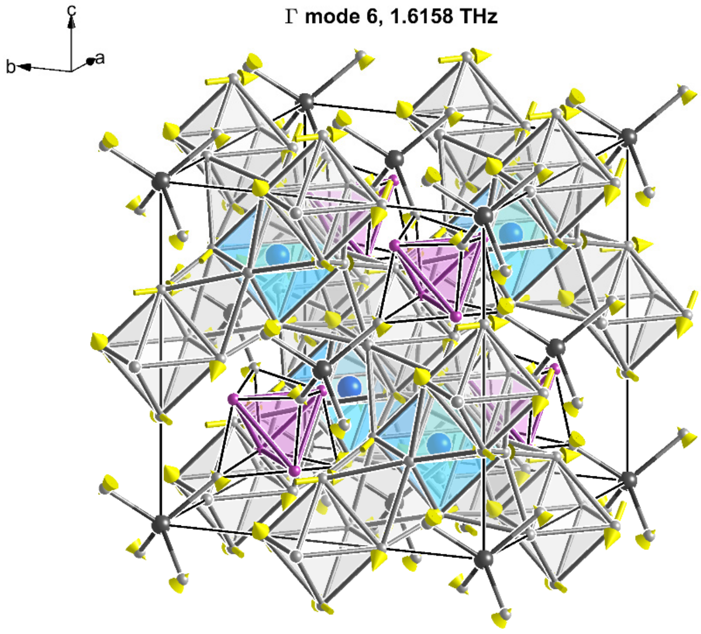

## Γ mode 7, 2.2235 THz

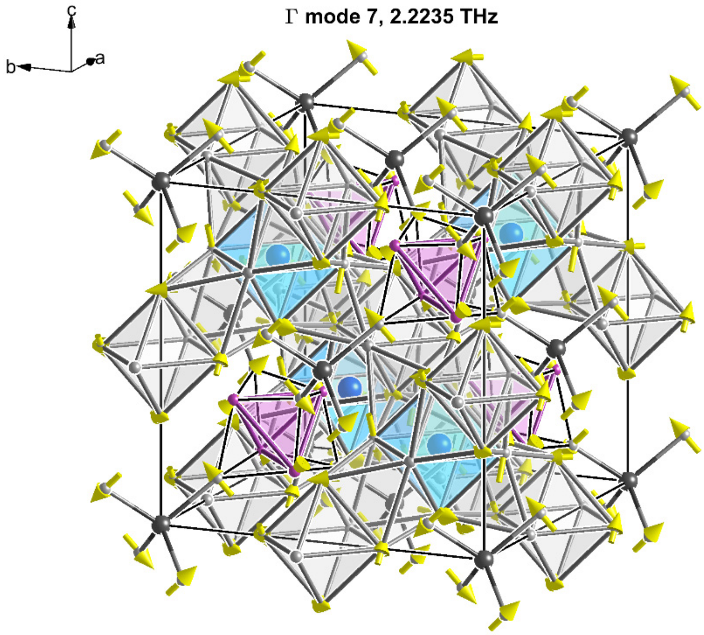


## Γ mode 8, 2.2235 THz

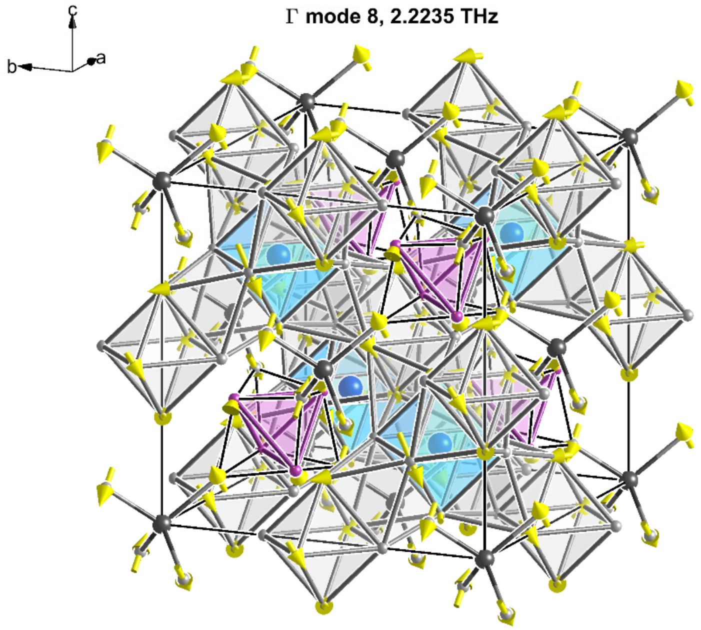

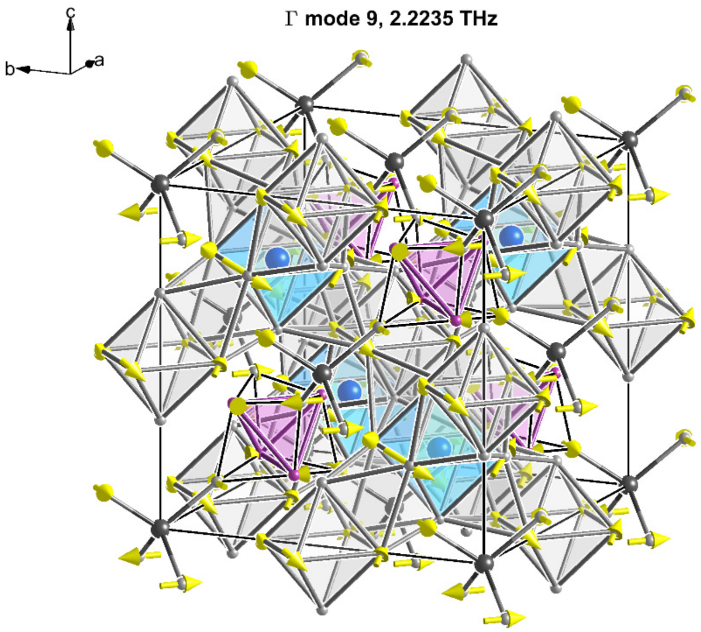
c
b
a
Γ mode 9, 2.2235 THz

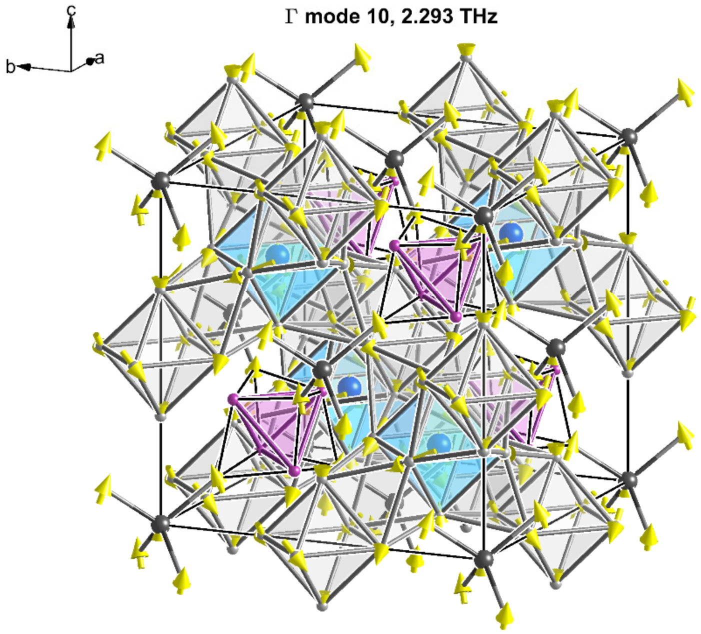
c
b
a
Γ mode 10, 2.293 THz

Γ **mode 11, 2.293 THz**

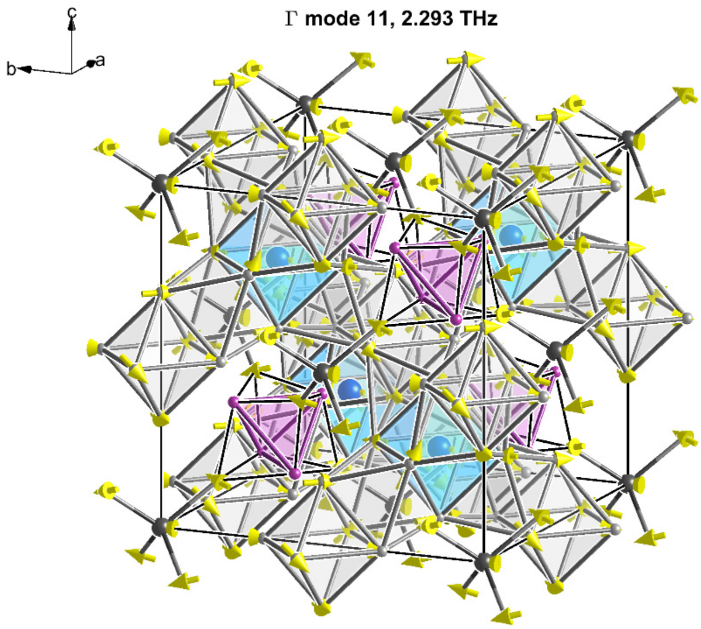


Γ **mode 12, 2.293 THz**

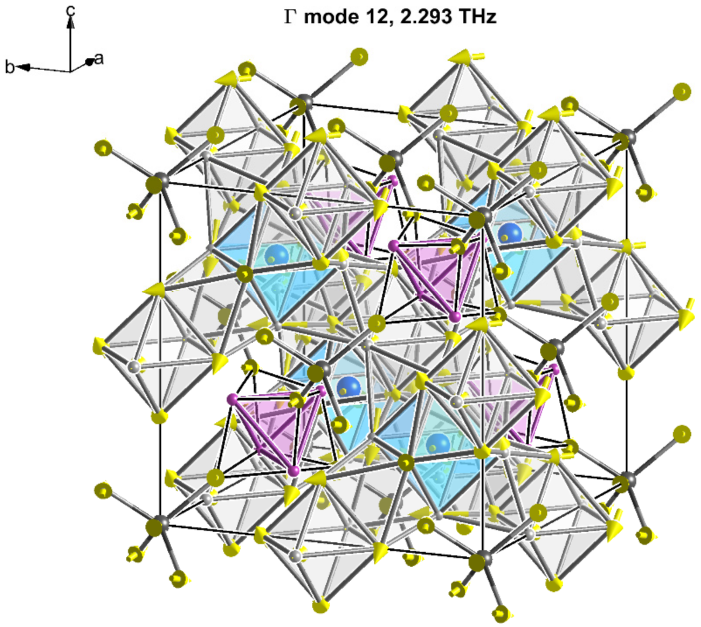

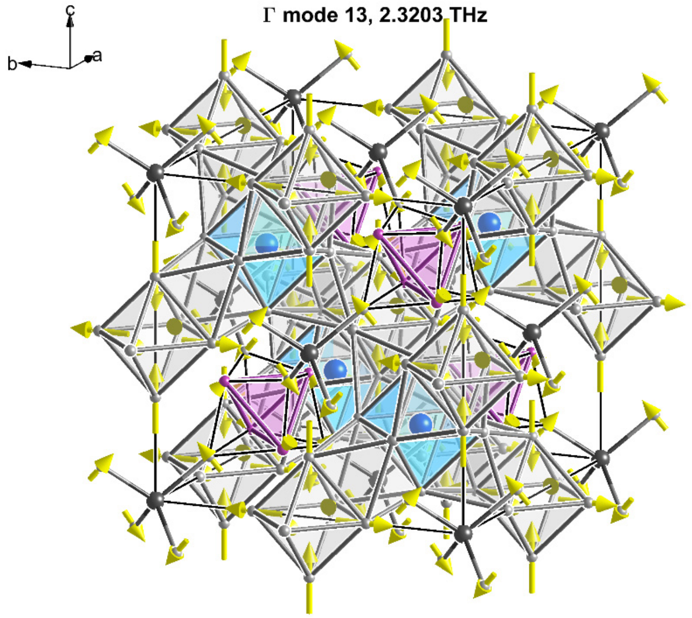
c
b
a
Γ mode 13, 2.3203 THz

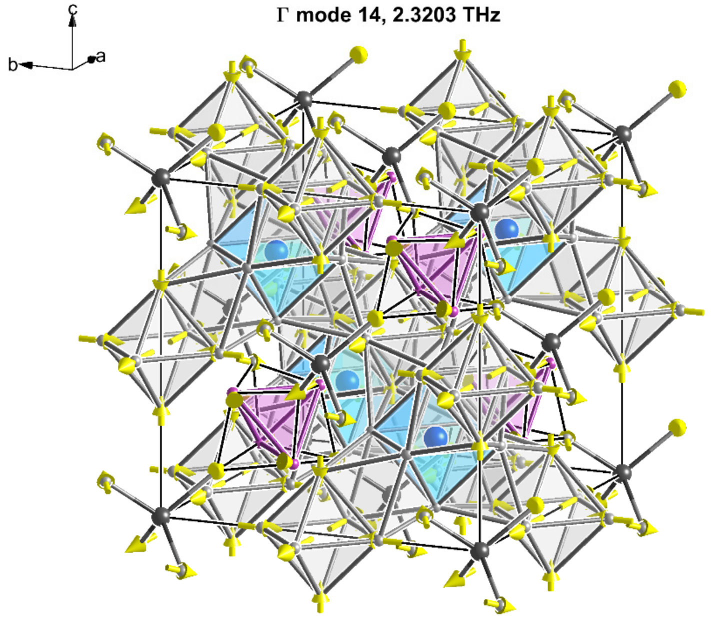
c
b
a
Γ mode 14, 2.3203 THz

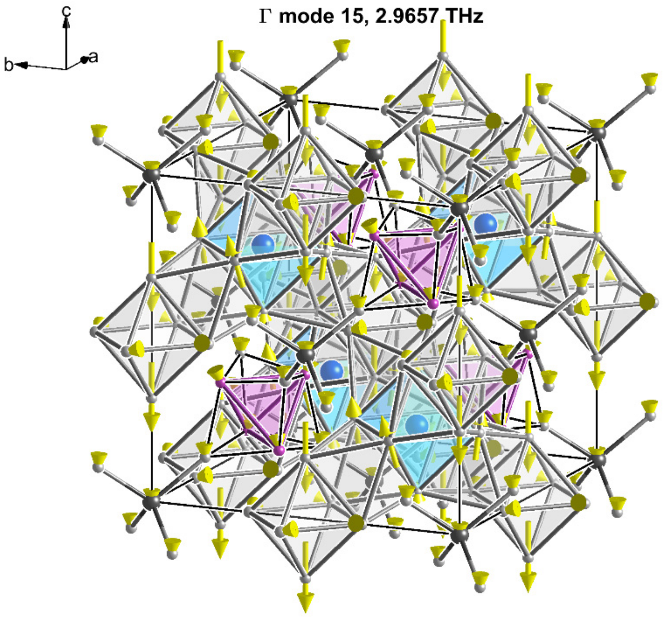
Γ mode 15, 2.9657 THz
c
b
a

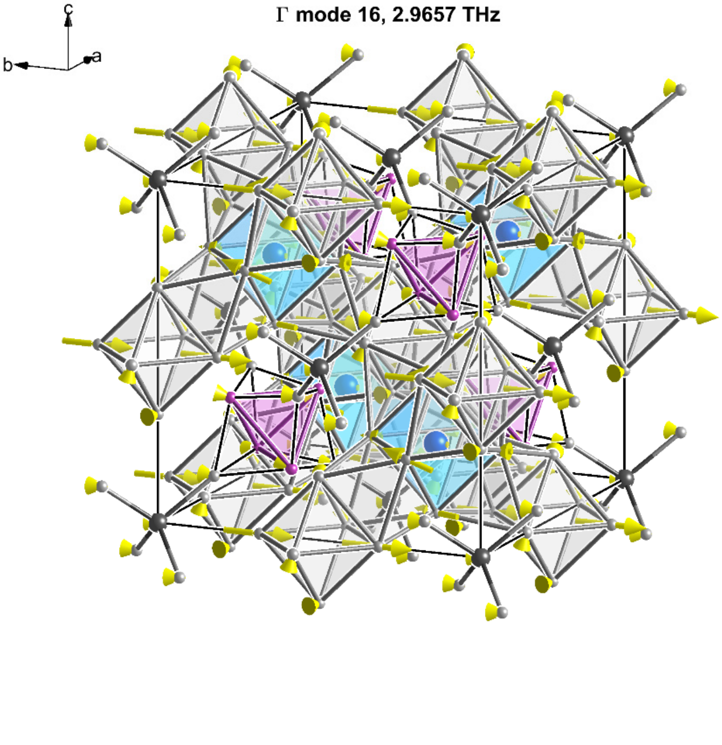
Γ mode 16, 2.9657 THz
c
b
a

Γ mode 17, 2.9657 THz

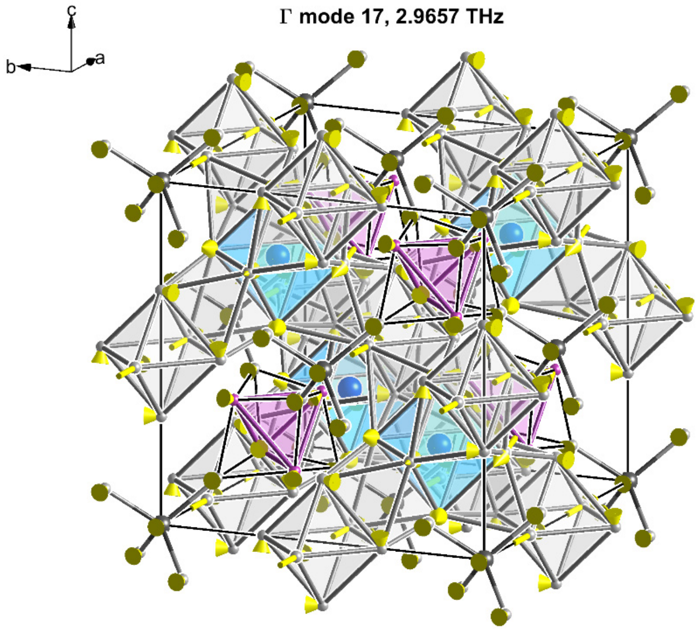


Γ mode 18, 3.1449 THz

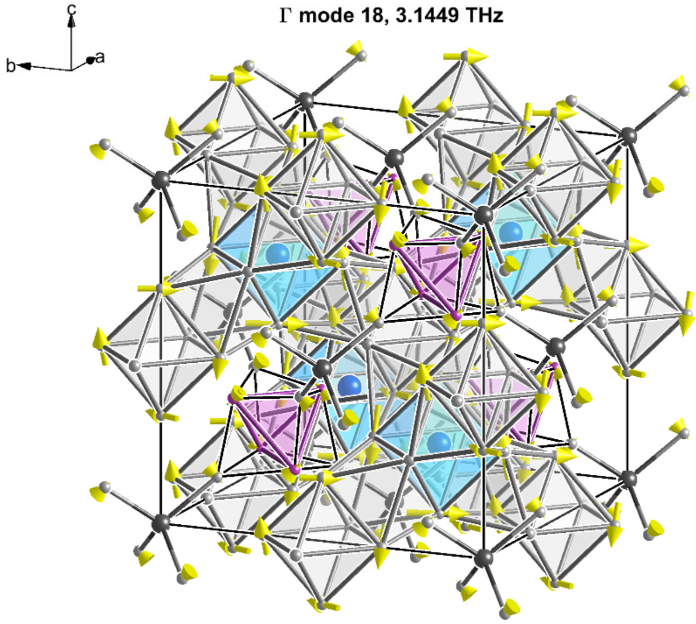

Γ mode 19, 3.1449 THz

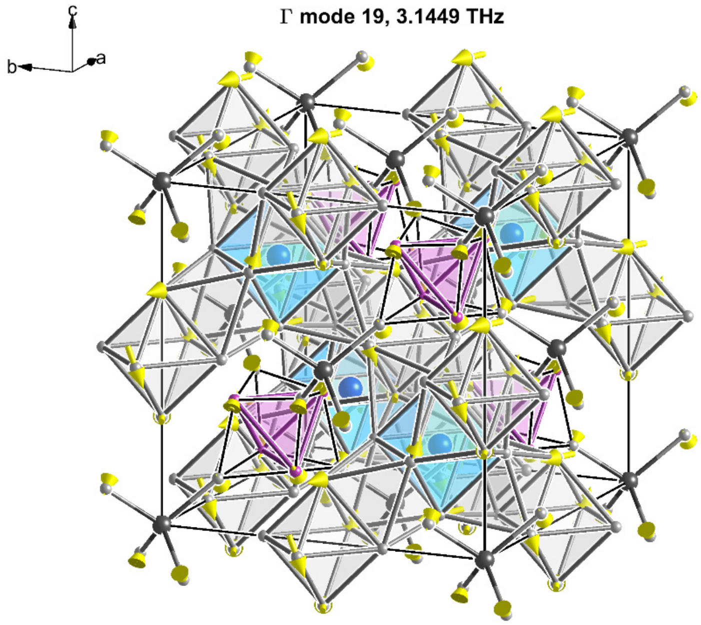


Γ mode 20, 3.1449 THz

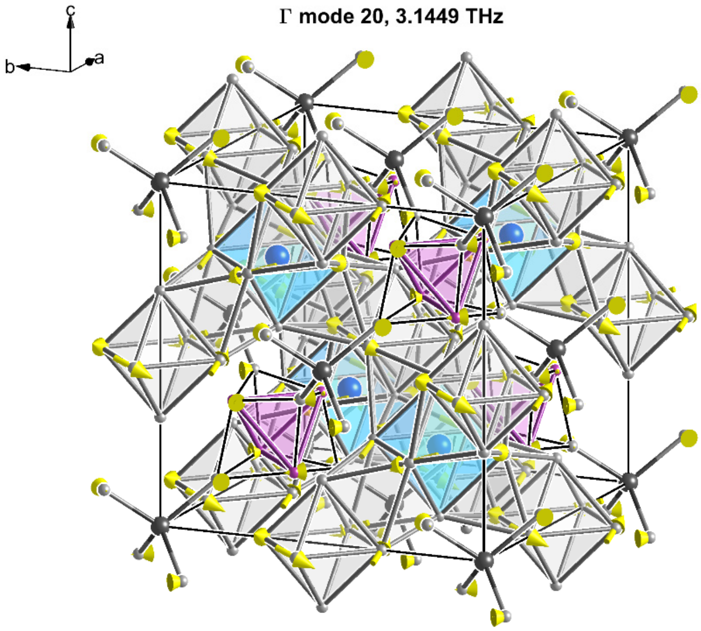

**Γ mode 21, 3.2629 THz**

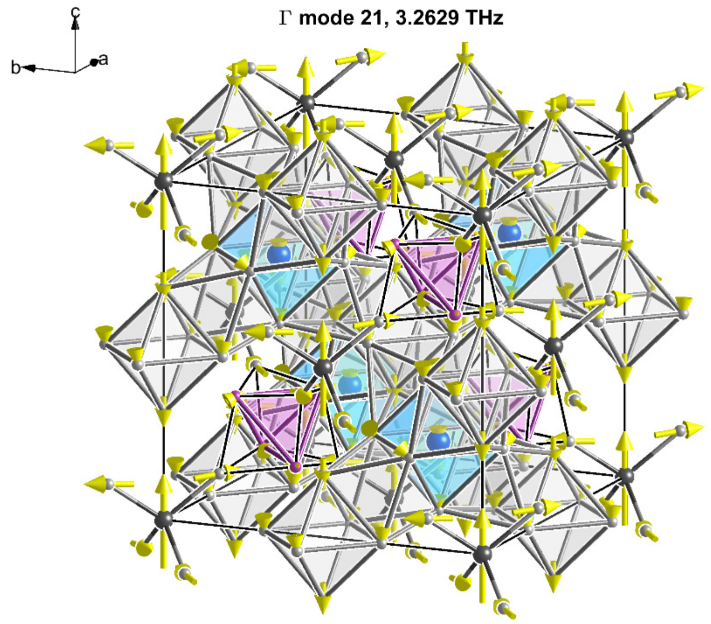


**Γ mode 22, 3.2629 THz**

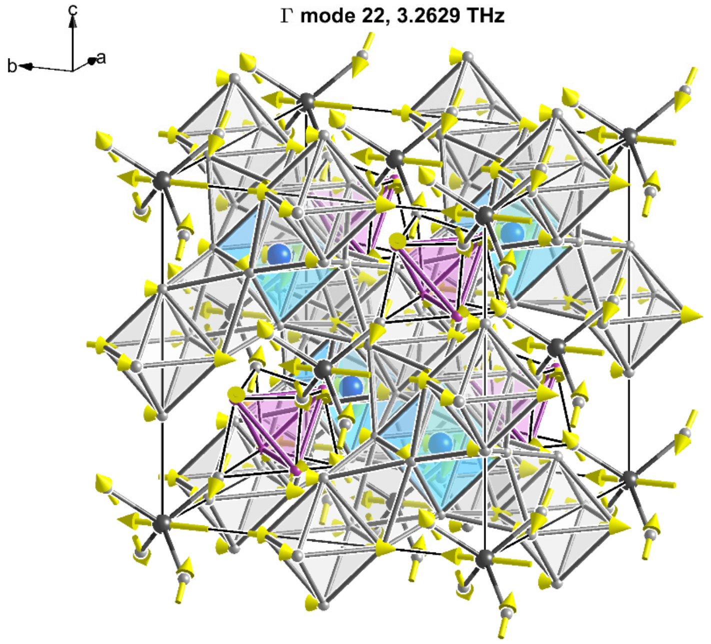

Γ mode 23, 3.2629 THz

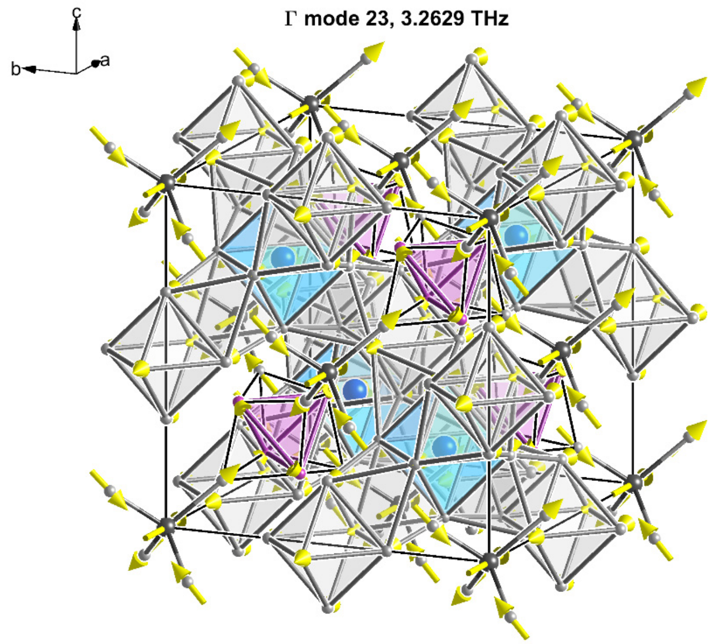


Γ mode 24, 3.5561 THz

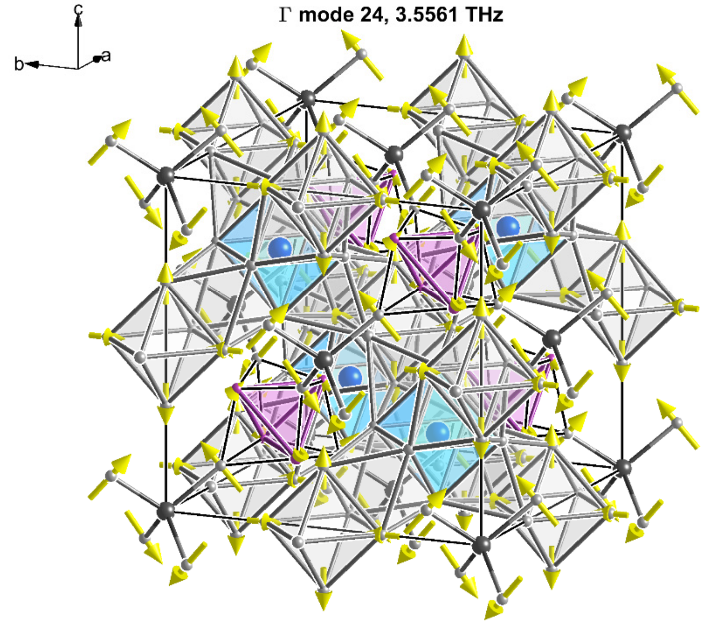

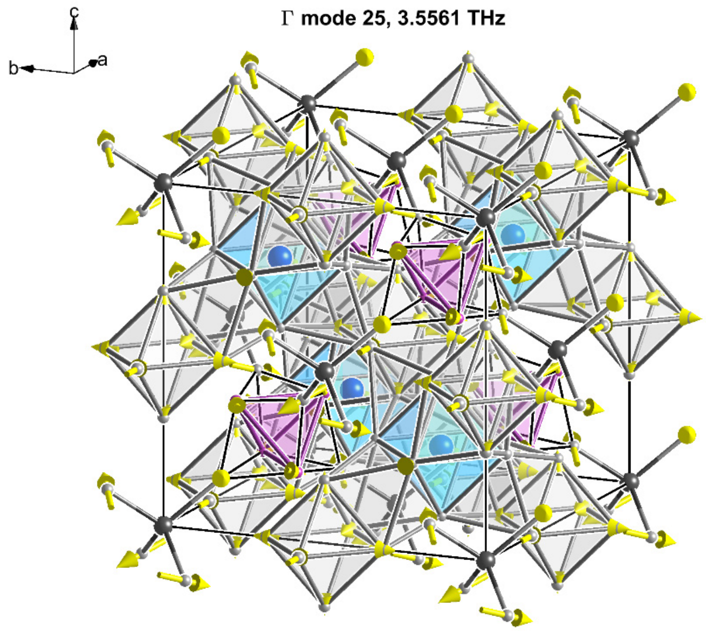
Γ mode 25, 3.5561 THz
c
b
a

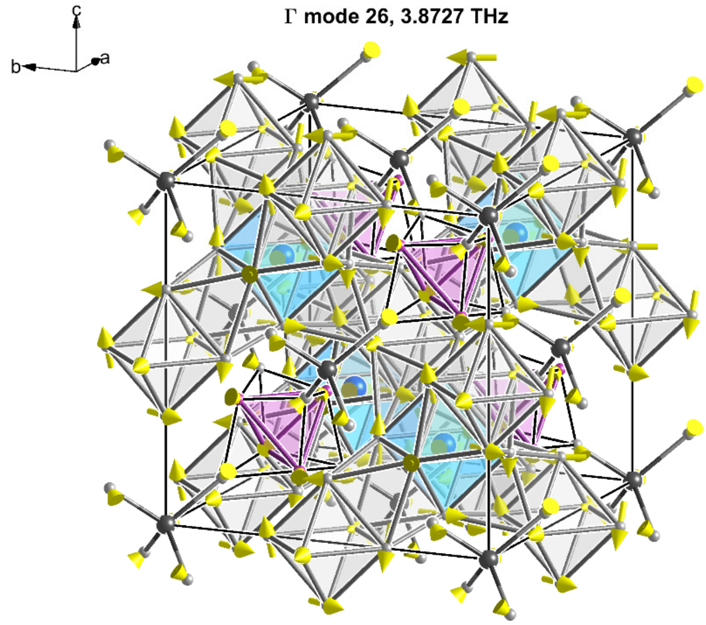
Γ mode 26, 3.8727 THz
c
b
a

Γ mode 27, 3.8727 THz

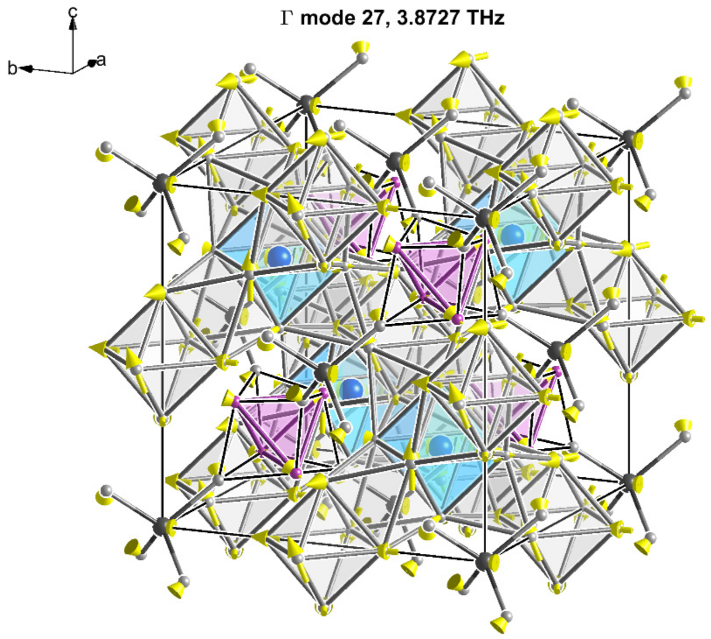


Γ mode 28, 3.8727 THz

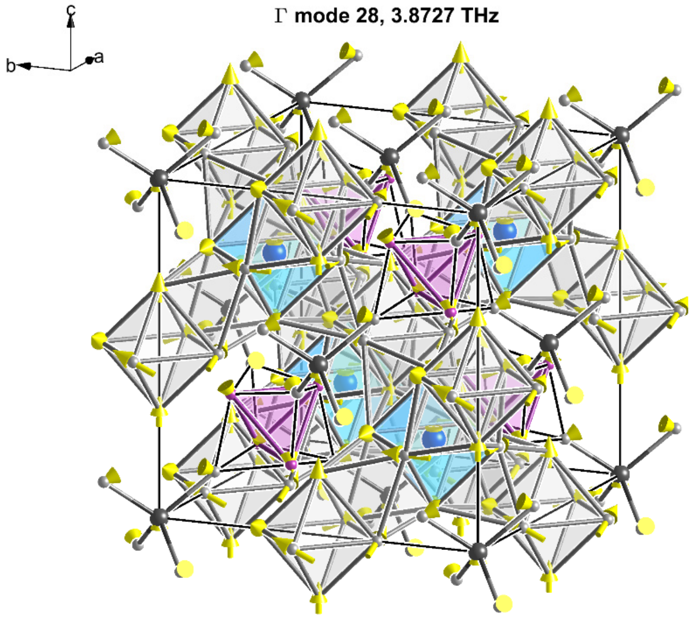

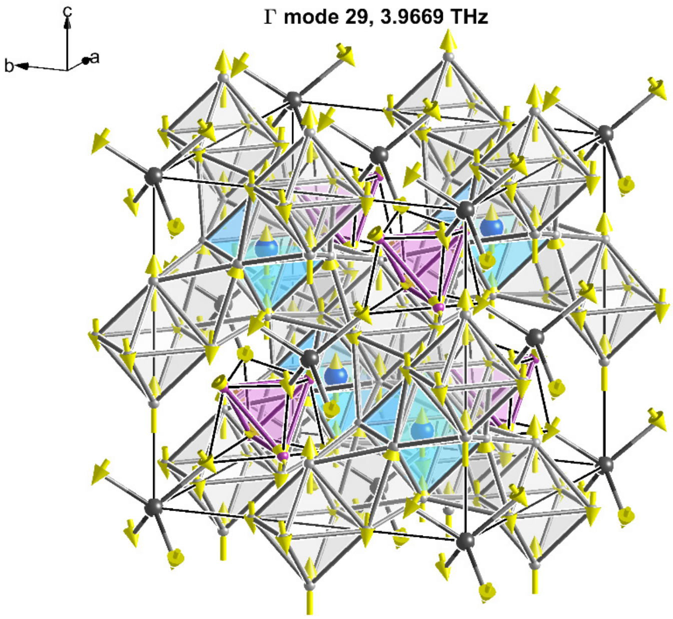
c
b
a
Γ mode 29, 3.9669 THz

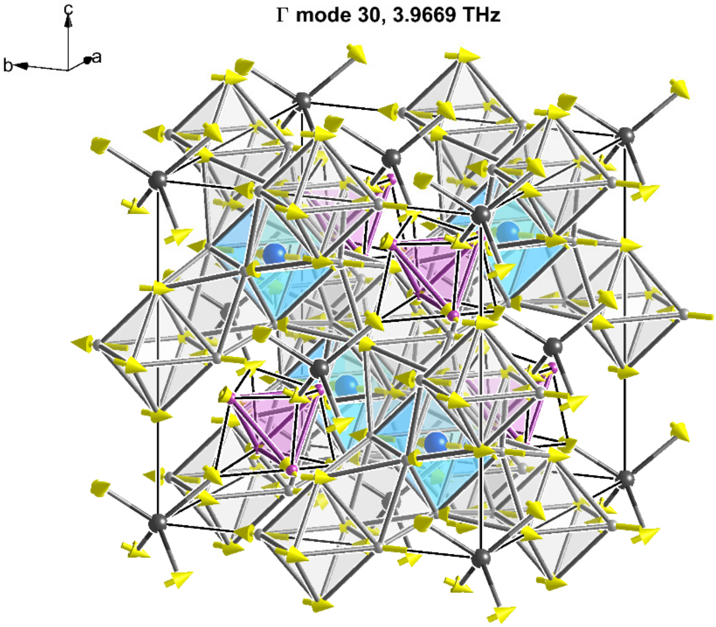
c
b
a
Γ mode 30, 3.9669 THz

**Γ mode 31, 3.9669 THz**

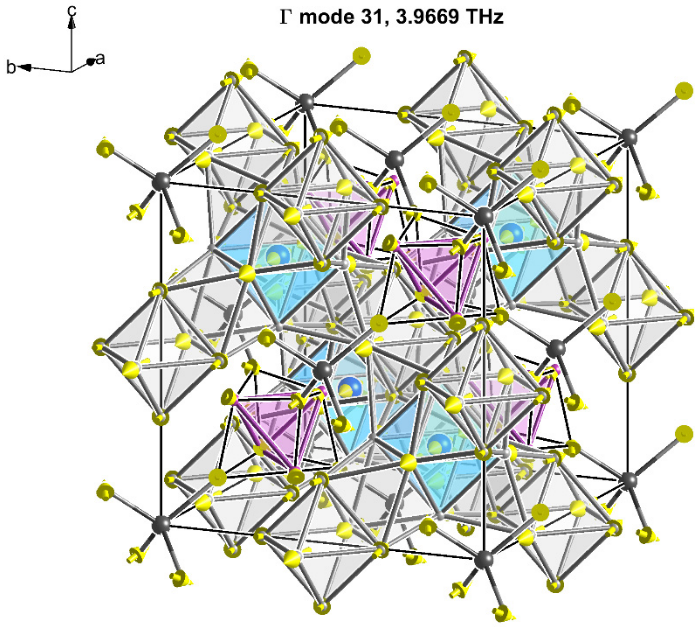


**Γ mode 32, 4.0489 THz**

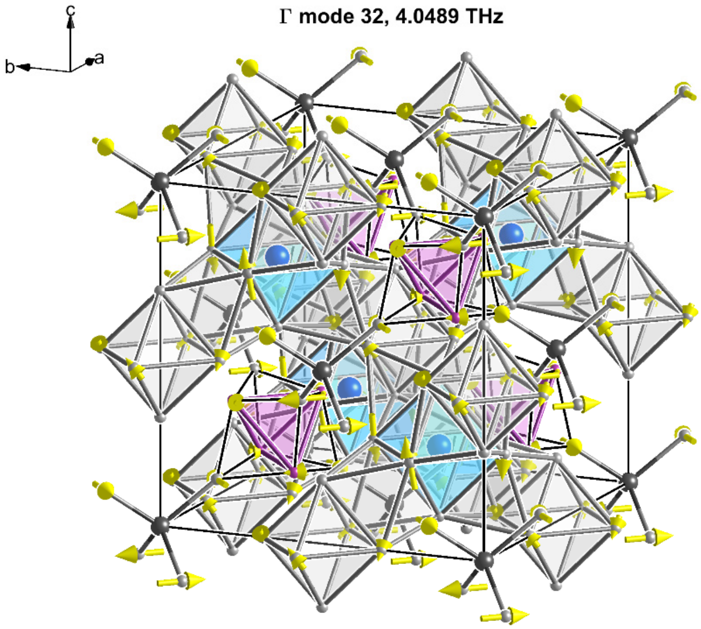

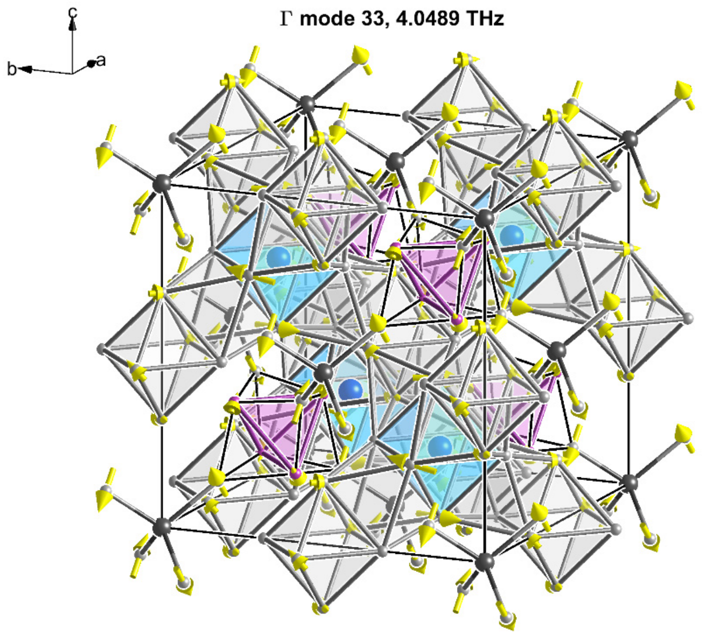

Γ mode 33, 4.0489 THz
c
b
a


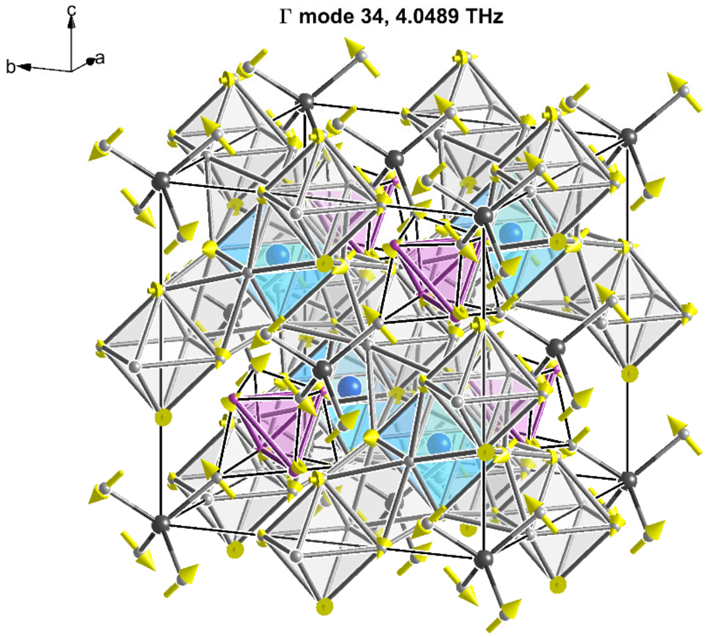

Γ mode 34, 4.0489 THz
c
b
a

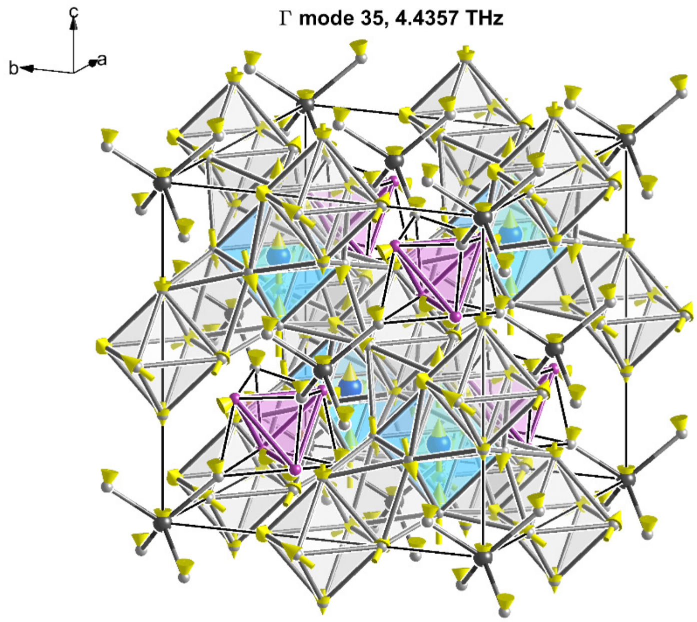
c
b
a
Γ mode 35, 4.4357 THz

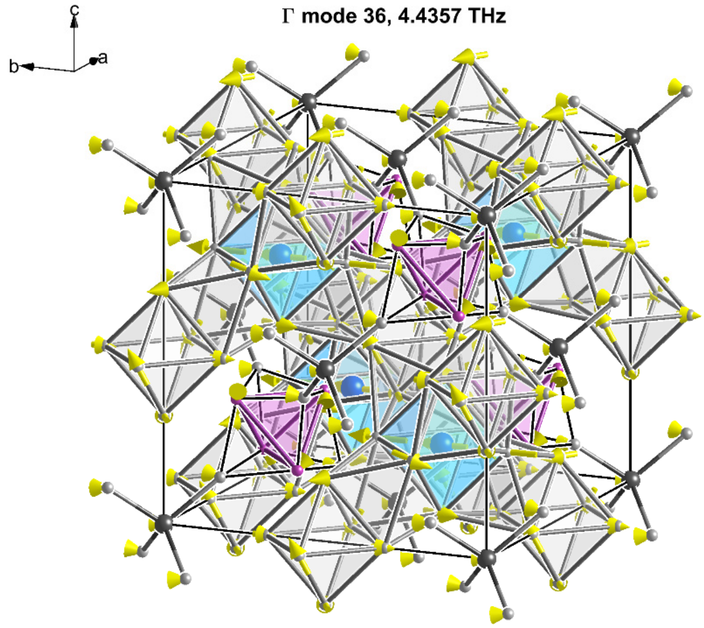
c
b
a
Γ mode 36, 4.4357 THz

**Γ mode 37, 4.4357 THz**

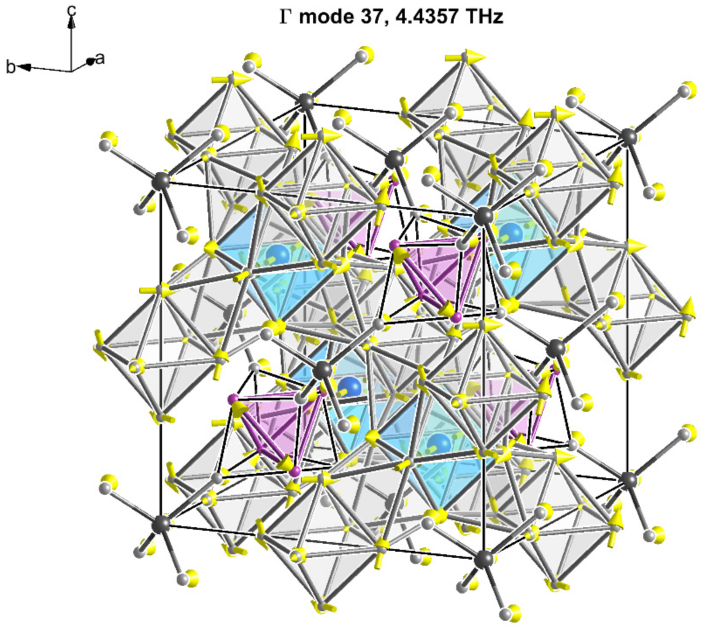


**Γ mode 38, 4.5099 THz**

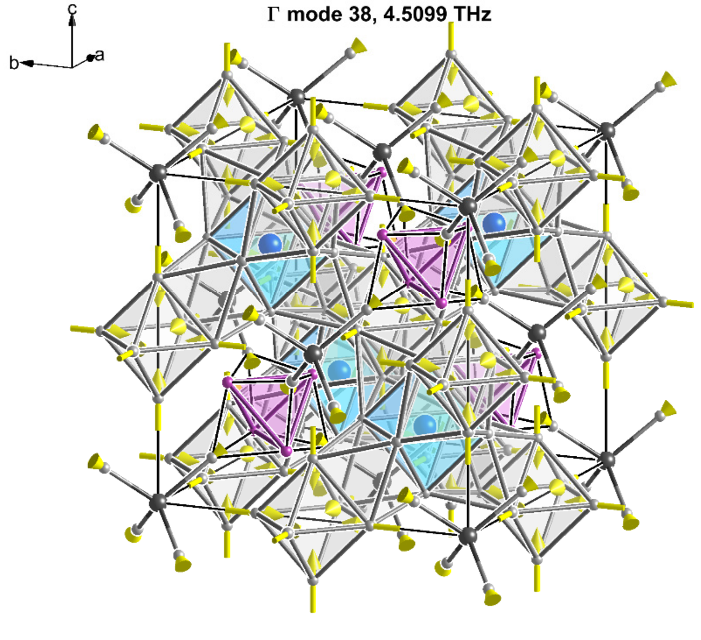

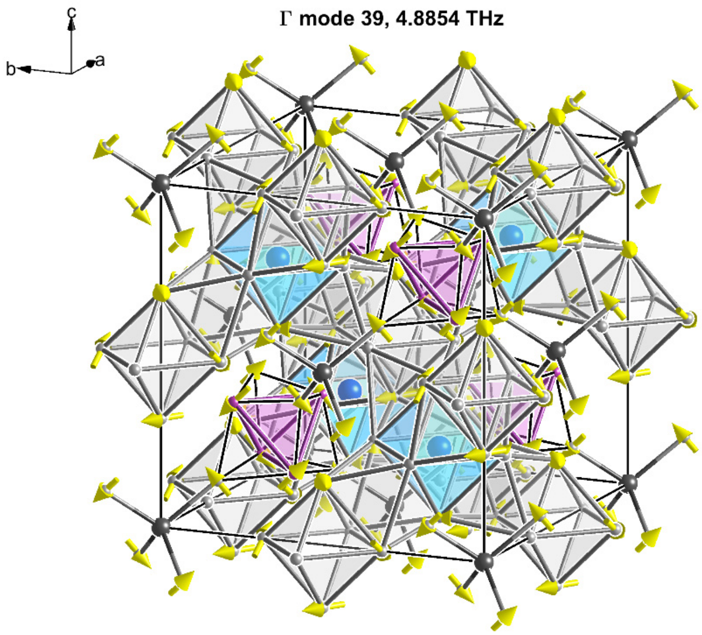
Γ mode 39, 4.8854 THz
c
b
a

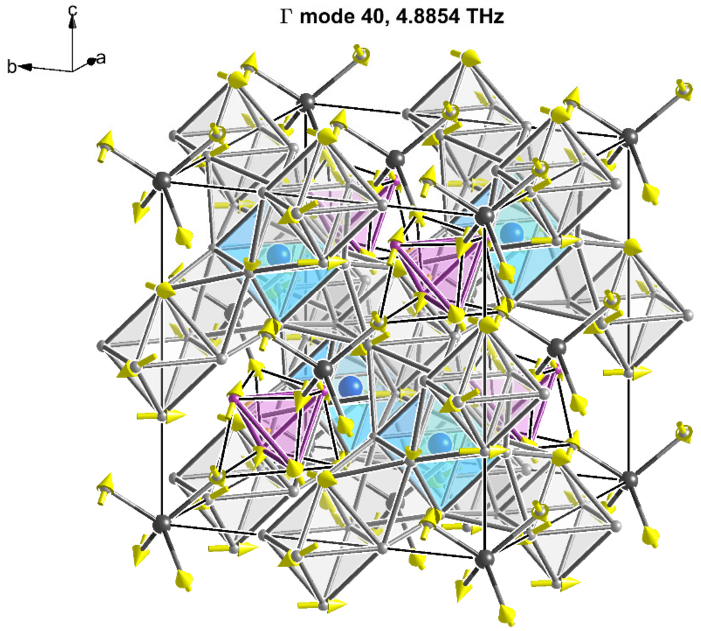
Γ mode 40, 4.8854 THz
c
b
a

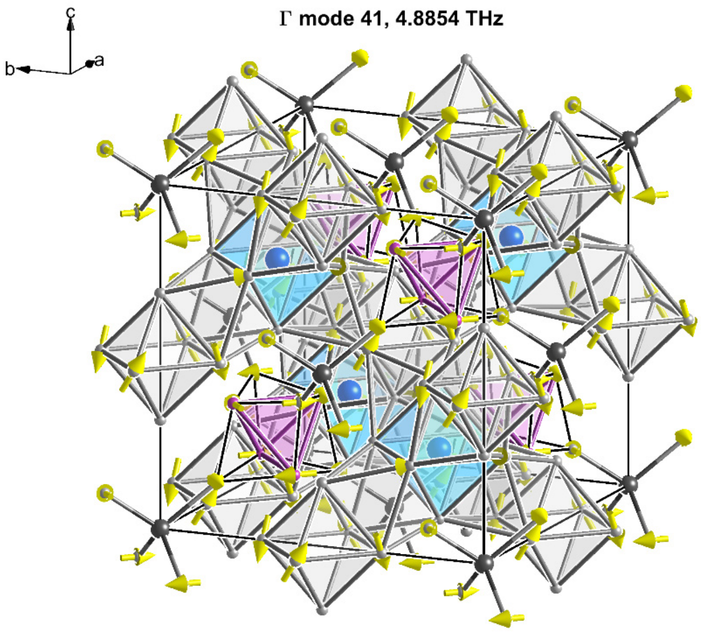
c
b
a
Γ mode 41, 4.8854 THz

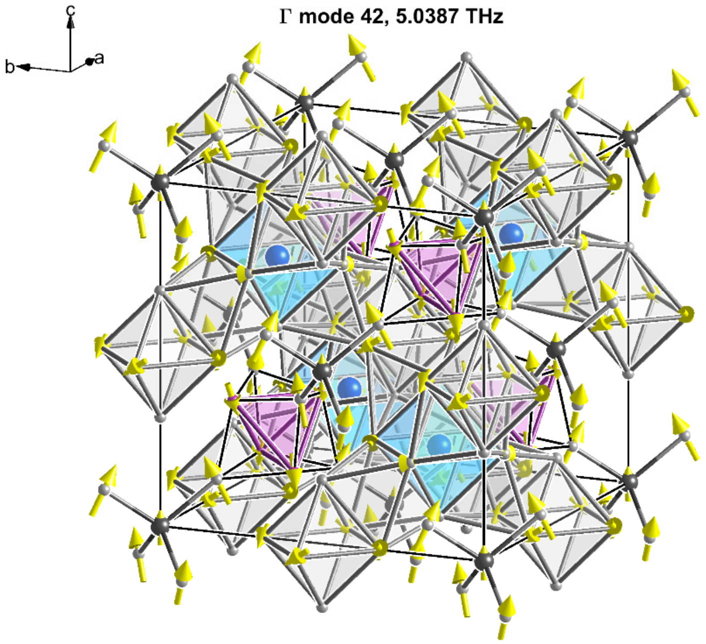
c
b
a
Γ mode 42, 5.0387 THz

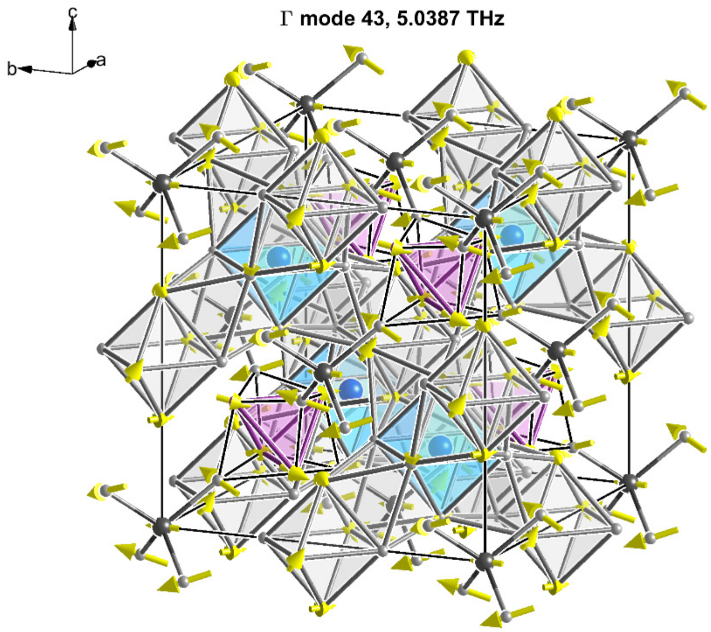
c
b
a
Γ mode 43, 5.0387 THz

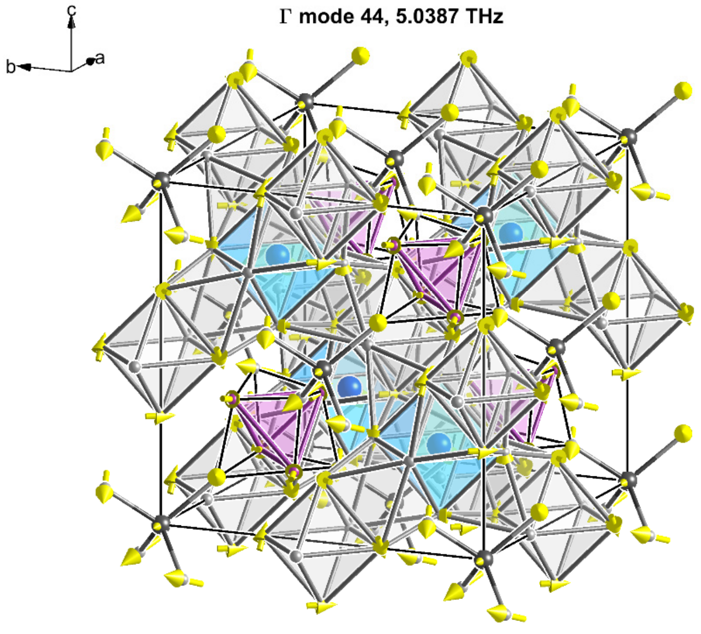
c
b
a
Γ mode 44, 5.0387 THz

Γ mode 45, 5.3502 THz

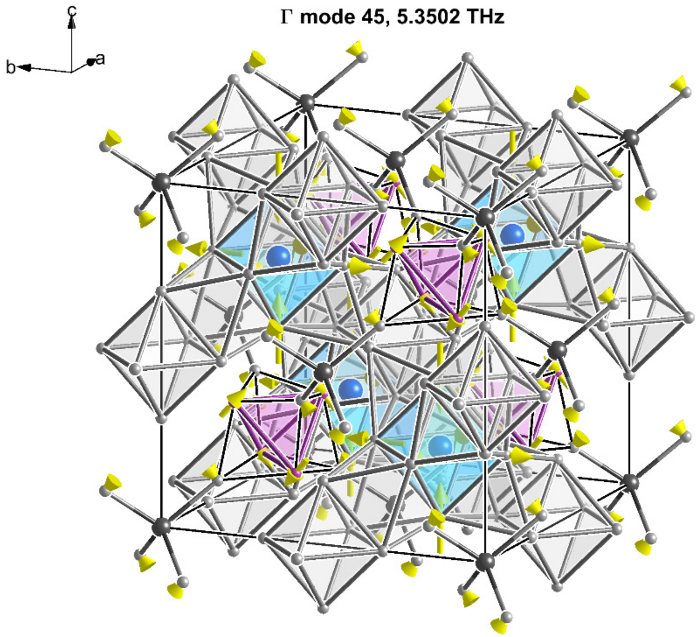


Γ mode 46, 5.3502 THz

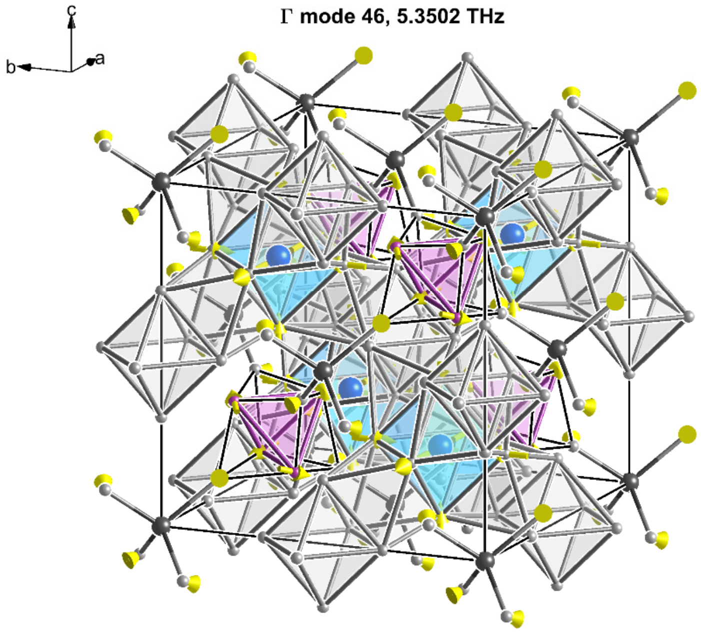

Γ mode 47, 6.2587 THz

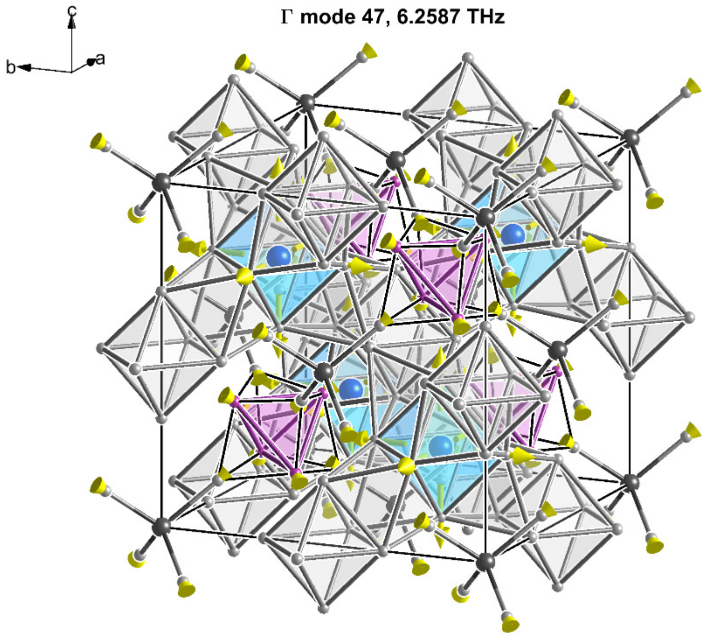


Γ mode 48, 6.3284 THz

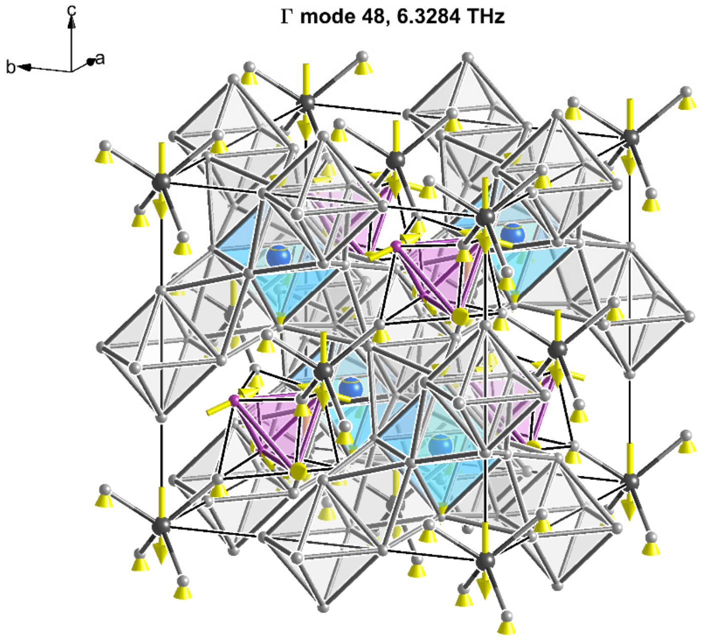

**Γ mode 49, 6.3284 THz**

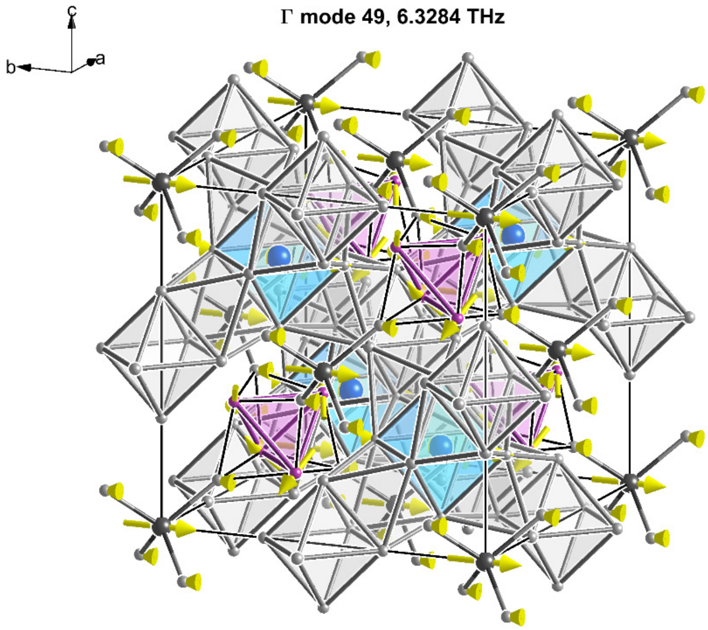


**Γ mode 50, 6.3284 THz**

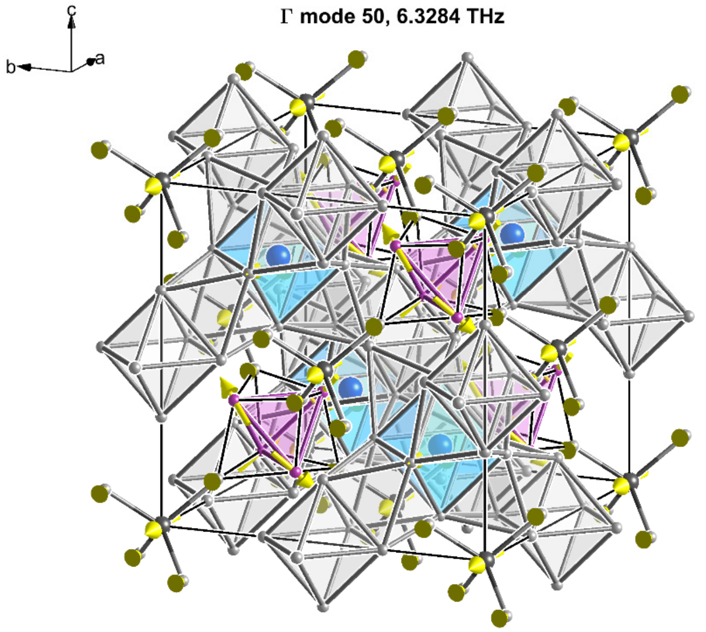

**Γ mode 51, 6.4443 THz**

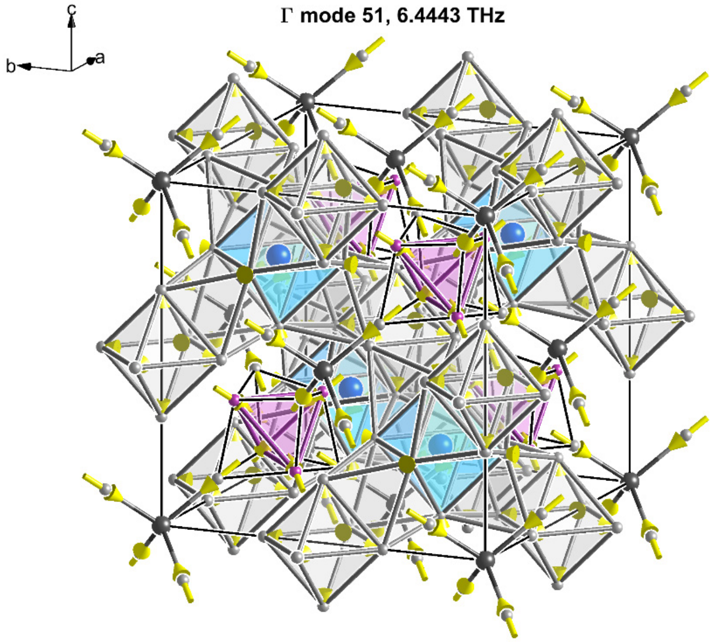


**Γ mode 52, 6.7581 THz**

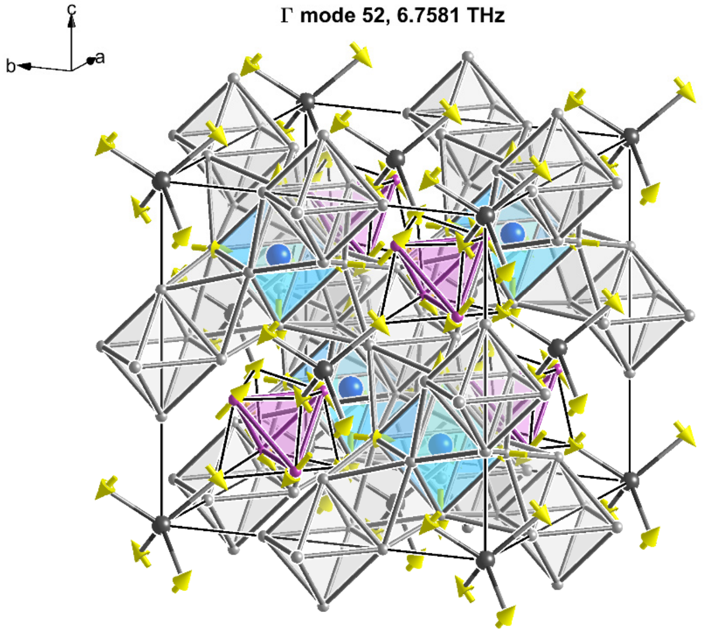

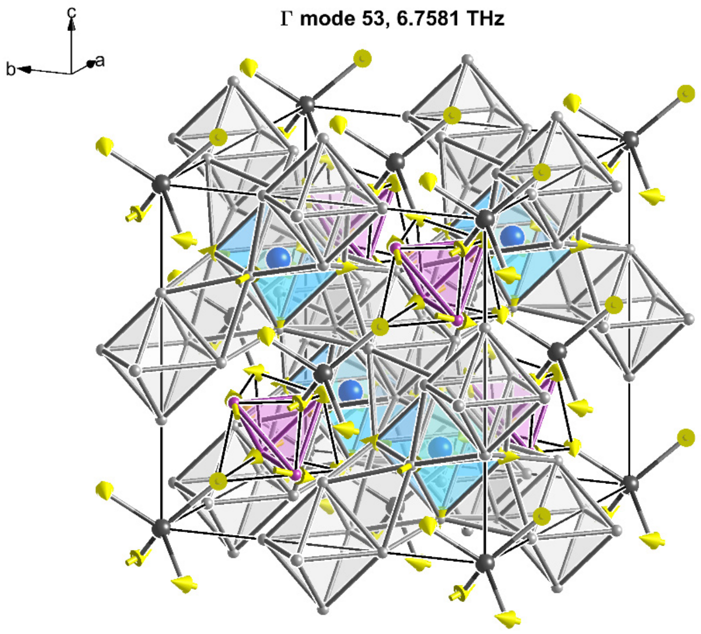
Γ mode 53, 6.7581 THz
c
b
a

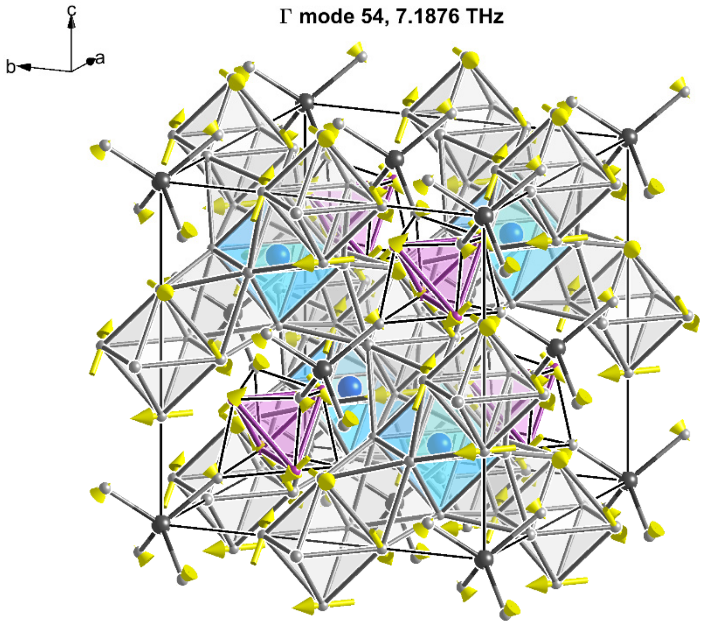
Γ mode 54, 7.1876 THz
c
b
a

Γ mode 55, 7.1876 THz

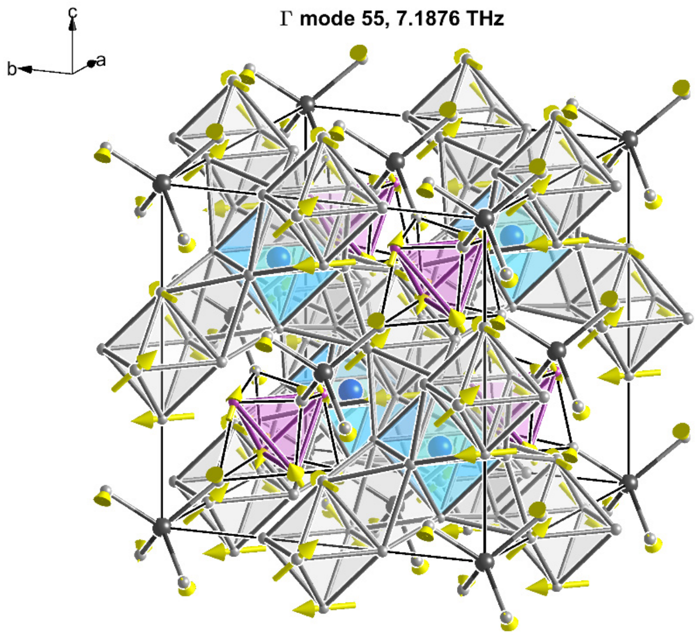


Γ mode 56, 7.1876 THz

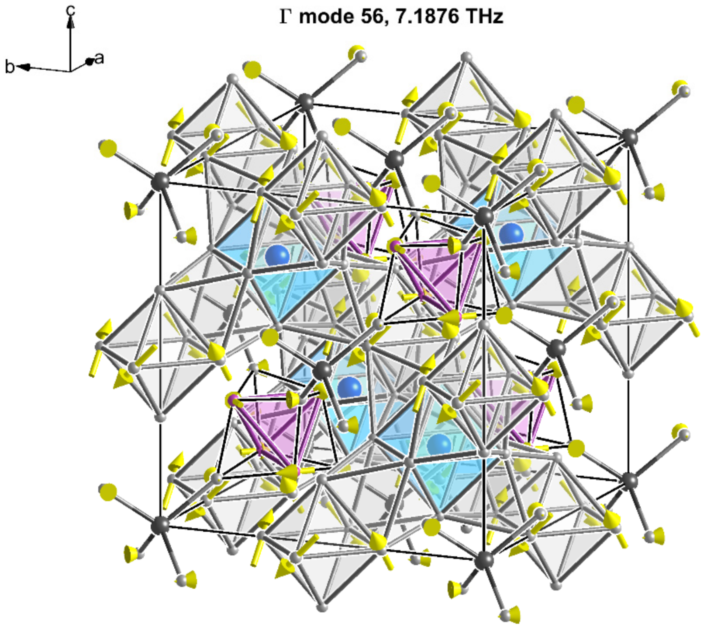

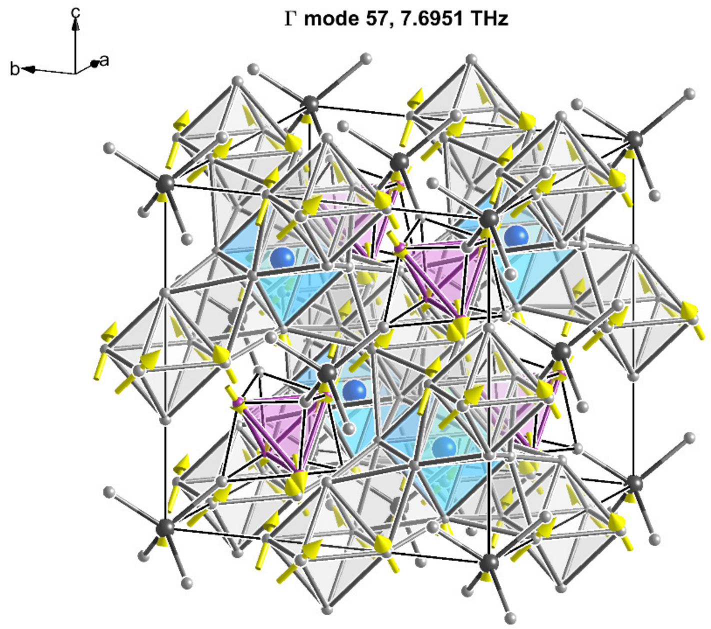
Γ mode 57, 7.6951 THz
c
b
a

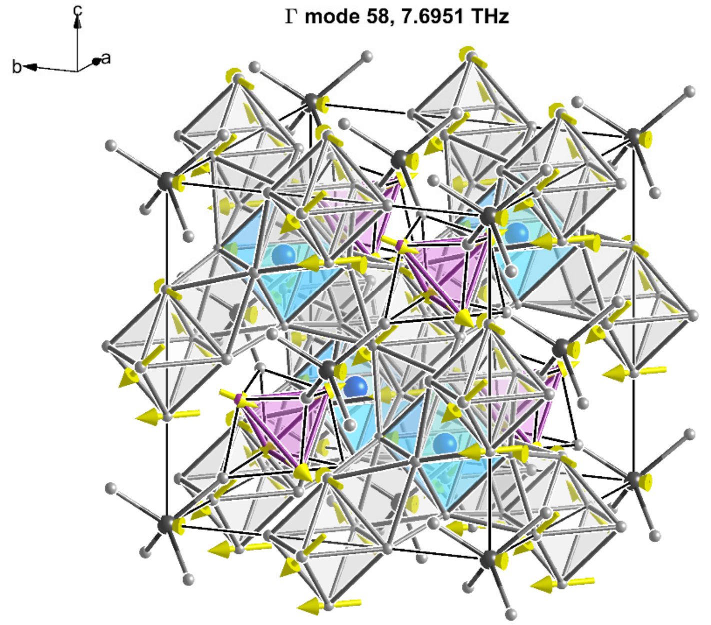
Γ mode 58, 7.6951 THz
c
b
a

Γ mode 59, 7.6951 THz

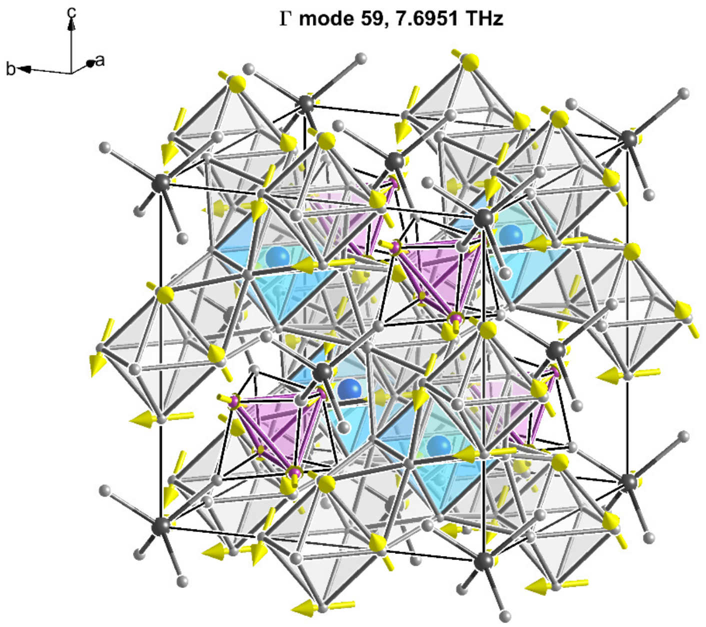


Γ mode 60, 8.1914 THz

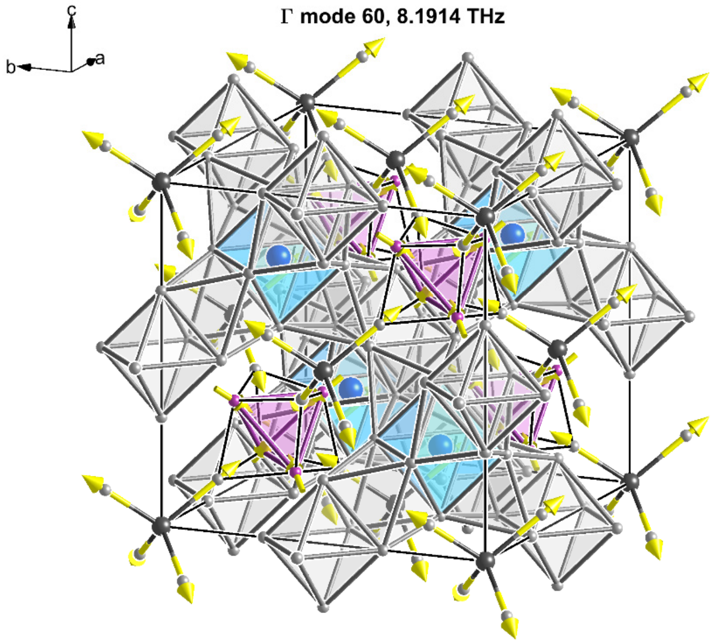

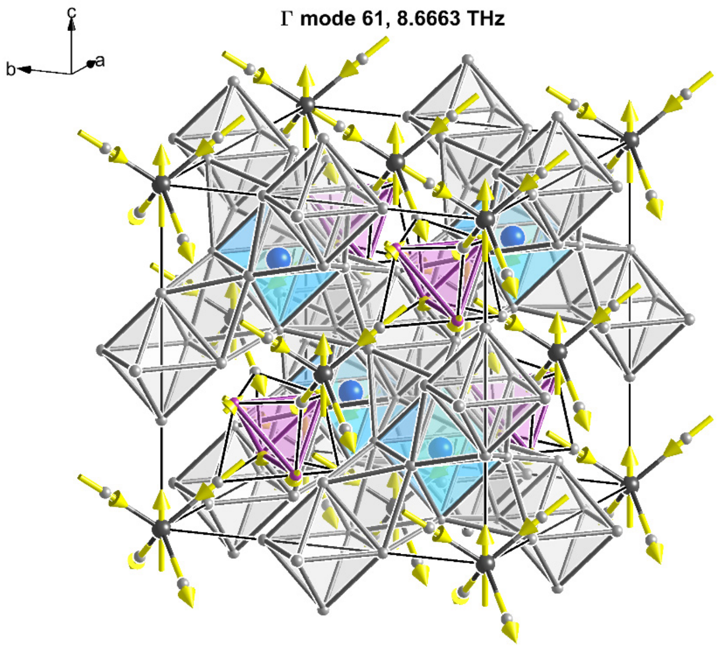
Γ mode 61, 8.6663 THz
c
b
a

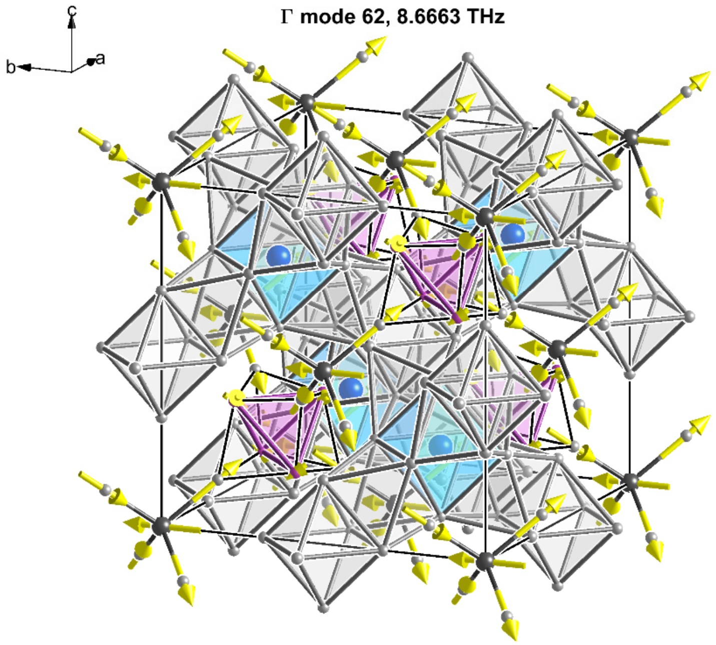
Γ mode 62, 8.6663 THz
c
b
a

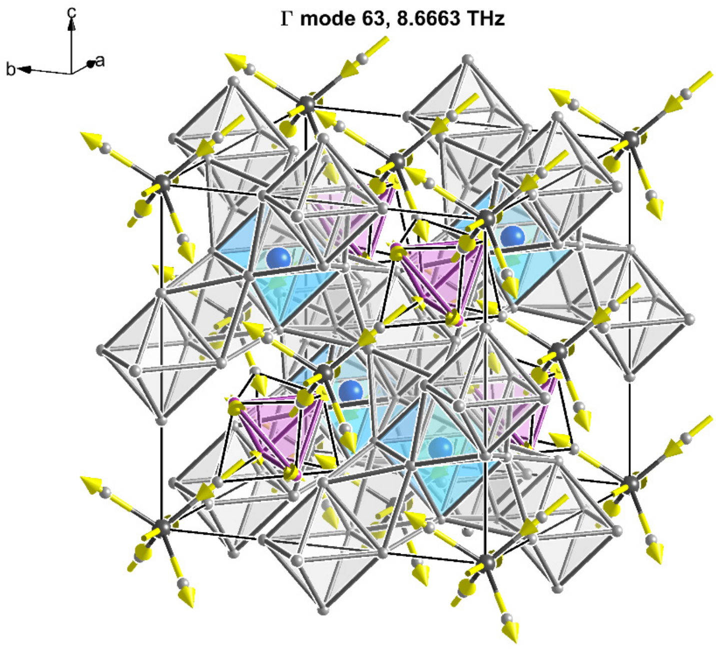
Γ mode 63, 8.6663 THz
c
b
a

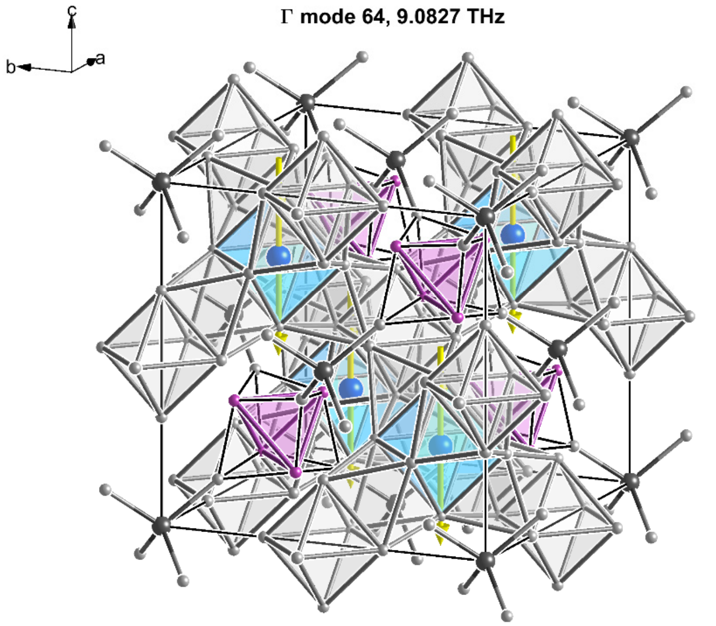
Γ mode 64, 9.0827 THz
c
b
a

**Γ mode 65, 9.0827 THz**

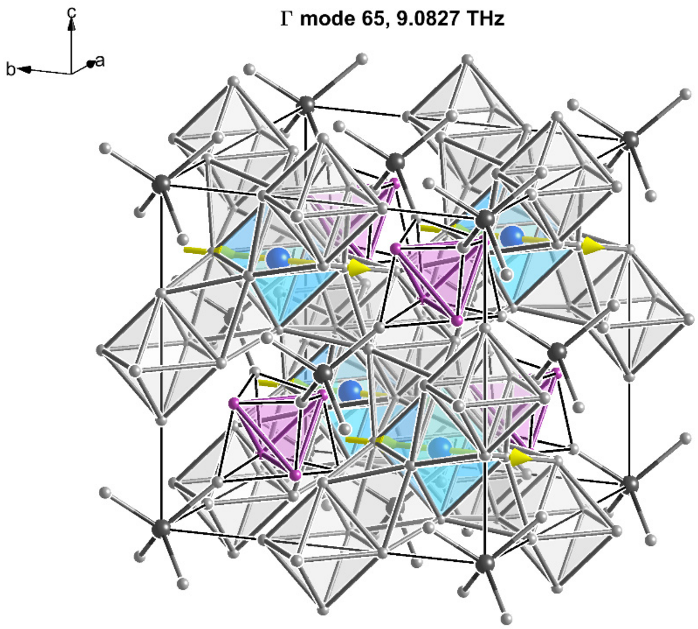


**Γ mode 66, 9.0827 THz**

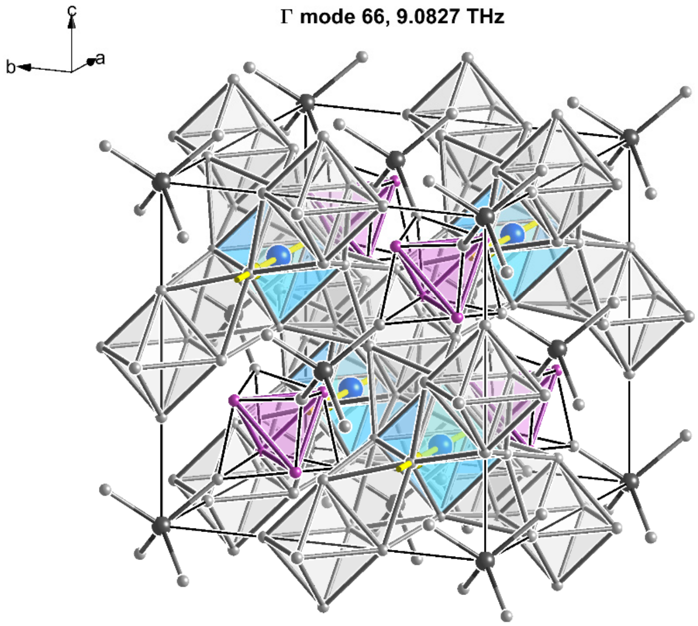

Supplement: Supplementary file 1 [file cm5c03434_si_001.pdf]
